# Synthesis and Evaluation of *N-*(3-Trifluoroacetyl-indol-7-yl) Acetamides for Potential In Vitro Antiplasmodial Properties

**DOI:** 10.3390/molecules22071099

**Published:** 2017-07-02

**Authors:** Malose J. Mphahlele, Mmakwena M. Mmonwa, Yee Siew Choong

**Affiliations:** 1 Department of Chemistry, College of Science, Engineering and Technology, University of South Africa, Private Bag X06, Florida 1710, South Africa; mmonwmm@unisa.ac.za; 2 Institute for Research in Molecular Medicine (INFORMM), Universiti Sains Malaysia, 11800 Minden, Penang, Malaysia; yeesiew@usm.my

**Keywords:** *N*-(2,5-diaryl-1*H*-indol-7-yl)acetamides, *N-*(3-trifluoroacetylindol-7-yl)acetamides, antiplasmodial activity, cytotoxicity, molecular docking

## Abstract

A series of novel *N*-((2,5-diaryl-3-trifluoroacetyl)-1*H*-indol-7-yl)acetamides has been prepared via a successive and one-pot reaction sequence involving initial trifluoroacetic acid-mediated Beckmann rearrangement of the oximes derived from the 1-(2,5-diaryl-1*H*-indol-7-yl)ethanones, followed by trifluoroacetylation of the incipient *N*-(2,5-diaryl-1*H*-indol-7-yl)-acetamides with trifluoroacetic anhydride. The prepared compounds were evaluated for potential in vitro antiplasmodial properties. Preliminary results from antiplasmodial activity against the chloroquine-sensitive 3D7 strain of *Plasmodium falciparum* revealed that a combination of 2-(4-flurophenyl)- and 5-(4-fluorophenyl) or 2-(4-flurophenyl)- and 4-fluorostyryl groups in compounds **3**(**a**,**f**) and **4**(**a**,**g**), for example, is required for biological activity for both series of compounds. Their possible mode of action against the plasmodial parasite is explained theoretically through molecular docking of the most active compounds against the parasite lactate dehydrogenase (pLDH). These compounds were docked at the entrance of NAD+ in pLDH presumably hindering entry of lactate to cause the observed inhibition effect of pLDH. The four compounds were found to exhibit low toxicity against monkey kidney Vero cells at the highest concentrations tested.

## 1. Introduction

**Figure 1:**
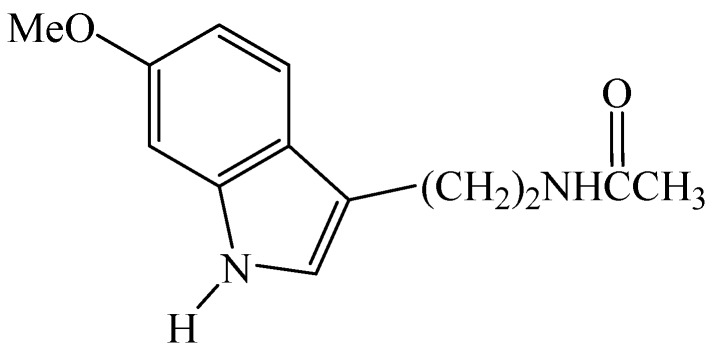
Structure of melatonin (**A**).

There has been growing interest in the development of efficient methods that allow for rapid access to functionalized indoles with different substitution pattern on the heterocyclic and aromatic rings due to their diverse biological properties, which include antimicrobial, antimalarial, anti-oxidant, anticancer and antitubercular activities [1,2,3]. The 7-aminoindole moiety constitutes an important scaffold for the development of compounds with antimalarial and anti-oxidant activities [3,4,5,6,7] and some of the compounds serve as anion receptors especially for dihydrogen phosphate [8]. Melatonin (**A**), which is shown in Figure 1, is an example of a carboxamide-appended indole derivative which plays a central role in the control of *Plasmodium falciparum* replication and establishment of parasitemia [9]. The presence of a hydrogen-bond acceptor at the C-3 position of an indole moiety such as carbonyl group is important for interaction with the biological receptors [10].

In a previous study we evaluated some 1-(2,5-diarylindol-7-yl)ethanones for in vitro cytotoxicity against the human breast adenocarcinoma (MCF-7) and human cervical cancer (HeLa) cell lines [11]. In an effort to synthesize new compounds with antimalarial potential, we decided to transform the 1-(2,5-diarylindol-7-yl)ethanones into analogues of melatonin. Our strategy towards the indole-appended acetamides involved the Beckmann rearrangement of oximes derived from the 1-(2,5-diarylindol-7-yl)ethanones to afford the *N-*(indol-7-yl)acetamides. The latter were, in turn, transformed via C-3 trifluoroacetylation to afford the corresponding *N*-(2,5-diaryl-3-trifluoro-acetylindol-7-yl)acetamides. The prepared *N*-(indol-7-yl)acetamides and their 3-trifluoroacetyl derivatives were evaluated for potential in vitro antiplasmodial properties against the chloroquine-sensitive 3D7 strain of *Plasmodium falciparum* to correlate structural variations to biological activity. The possible mode of action of these compounds against the plasmodium parasite is explained theoretically through molecular docking of the most active compounds against the parasite lactate dehydrogenase (pLDH).

## 2. Results and Discussion

### 2.1. Chemistry

**Scheme 1:**
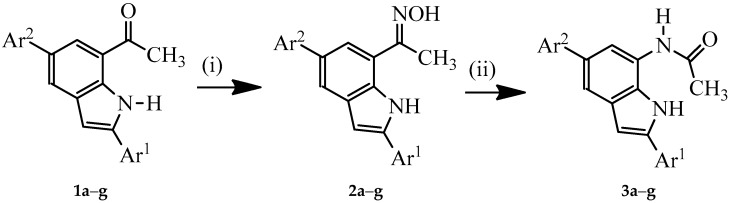
Beckmann rearrangement of the 1-(2,5-diarylindol-7-yl)ethanone oximes **2a**–**g**. *Reagents and conditions*: (**i**) NH_2_OH·HCl, pyridine, EtOH, reflux, 12 h; (**ii**) TFA, CH_3_CN, reflux, 2 h.

In order to prepare the *N*-(2,5-diarylindol-7-yl)acetamides, we required the oxime derivatives of compounds **1** for participation in the Beckmann rearrangement. We reacted the known 1-(2,5-diarylindol-7-yl)ethanones **1a**–**g** [11] with hydroxylamine hydrochloride in the presence of pyridine in ethanol under reflux and isolated the corresponding oximes **2a**–**g** by column chromatography on silica gel (Scheme 1). The oxime derivatives were characterised using a combination of NMR (^1^H and ^13^C) and IR spectroscopic techniques, complemented with mass spectrometry. Attempted Beckmann rearrangement of the keto-oximes **2** using molecular iodine in anhydrous acetonitrile [12] or triphenylphosphine-iodine catalyst complex in acetonitrile under reflux [13], led to the recovery of the starting material in both cases. We then decided to make use of a strong acid to promote the Beckmann rearrangement of compounds **2**. In this case we opted for the low boiling and water miscible trifluoroacetic acid (TFA) which has been used extensively in organic synthesis to promote other types of rearrangement reactions, functional group deprotections, oxidations, reductions, condensations, hydroarylations and trifluoromethylations [14]. TFA in aprotic solvents was previously found to promote the Beckmann rearrangement of keto-oximes into amides or lactams [15]. Based on this literature precedent, we subjected the oxime derivatives **2a**–**g** to TFA in acetonitrile under reflux for 2 h (Scheme 1). After aqueous workup and purification by silica gel column chromatography, we isolated compounds characterised using a combination of spectroscopic techniques as the desired novel *N*-(2,5-diarylindol-7-yl) acetamides **3a**–**g**. The proton NMR spectra of these amide derivatives revealed the presence of a singlet at about δ 9.70 ppm corresponding to NH of the amide group. Formation of these compounds through aryl migration, on the other hand, was confirmed by a significant up-field shift of the methyl protons to around δ 2.19 ppm compared to those in the ^1^H-NMR spectra of **1** (around δ 2.79 ppm) and **2** (around δ 2.38 ppm). Moreover, their carbonyl carbon resonates significantly up-field (δ_C=O_ 170 ppm) than that of the corresponding precursors **1** (δ_C=O_ 200 ppm) and more downfield than substrates **2** (δ_C=N_ 155 ppm). The methodology described in this investigation for the synthesis of C-7 aminated NH-free indoles is complementary to the method developed by Cacchi et al. [16], which makes use of the Buchwald-Hartwig C–N bond formation of the incipient 7-bromoindoles with primary and secondary amines. Series of 2,5- and 3,5-disubstituted 7-aminoindoles have also been prepared before *via* a three component Wittig reaction of pyrrole-3-carboxaldehydes with fumaronitrile and PEt_3_ followed by chemoselective C-6 alkylation of the incipient *cis*-allylic nitriles and subsequent cyclization through an intramolecular Houben−Hoesch reaction [17].

**Scheme 2:**
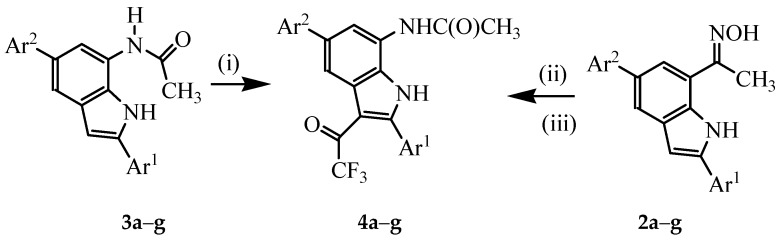
Synthesis of the *N*-(3-trifluoroacetylindol-7-yl) acetamides **4a**–**g**. *Reagents and conditions*: (**i**) TFAA, THF, reflux, 5 h; (**ii**) TFA, CH_3_CN, reflux, 2 h; (**iii**) TFAA, reflux, 5 h.

**Figure 2:**
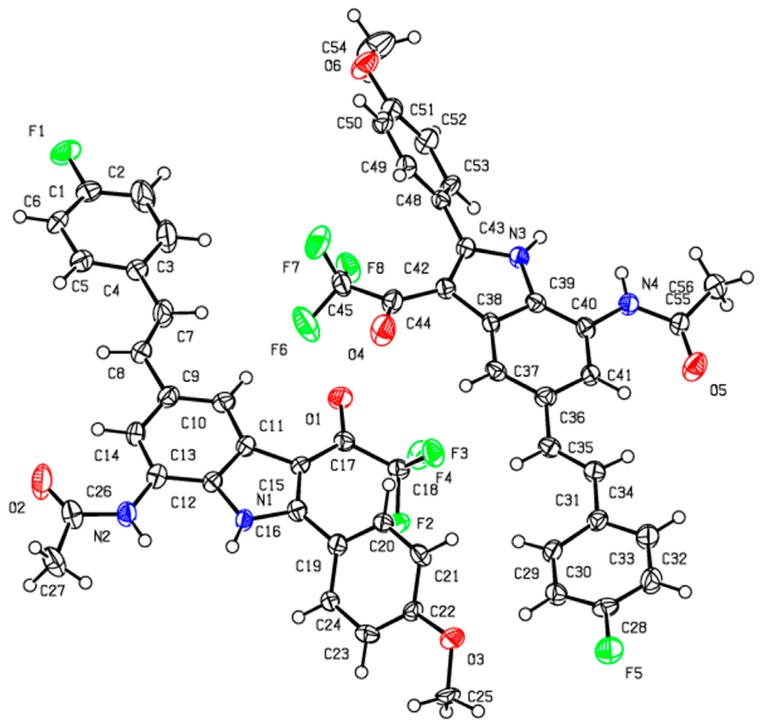
Oak Ridge Thermal Ellipsoid Plot (ORTEP) diagram of **4g**. Displacement ellipsoids are drawn at the 50% probability level and H atoms are shown as small spheres of arbitrary radii.

Since the presence of a hydrogen-bond acceptor at the C-3 position of an indole moiety such as carbonyl group is important for interaction with the biological receptors [10], we decided to incorporate a trifluoroacetyl group at the 3-position of the ambident nucleophilic indole framework of the *N*-(2,5-diarylindol-7-yl)acetamides **3a**–**g** to evaluate the resultant products for potential biological activity. We employed the literature conditions [18] on compounds **3a**–**g** and subjected them to TFAA (1.5 equiv.) in THF under reflux for 5 h (Scheme 2). Aqueous work-up and column chromatography on silica gel afforded the corresponding *N*-(3-trifluoroacetylindol-7-yl)acetamides **4a**–**g** in appreciable yields without traces of the *N*-substituted products. Incorporation of the trifluoroacetyl group was confirmed by the absence of a singlet for 3-H in the region δ 6.77–6.92 ppm found in the ^1^H-NMR spectra of the corresponding substrates and the presence of two set of quartets at around δ 116.0 ppm and δ 176 ppm in the ^13^C-NMR spectra of **4**, which correspond to the trifluoromethyl and carbonyl carbon signals, respectively. The presence of trifluoroacetyl group in compounds **4** was further confirmed by an intense peak at around δ −71.0 ppm in their ^19^F-NMR corresponding to -CF_3_ in analogy with the literature precedents for the analogous 3-trifluoroacetylindoles [19]. We explored the possibility to effect sequential Beckmann rearrangement and C-3 trifluoroacetylation of the oximes **2a**–**g** to afford compounds **4** in a one-pot operation. In this case, we subjected the oxime derivatives **2a**–**g** to TFA (1.2 equiv.) in acetonitrile under reflux for 2 h followed by addition of TFAA (2 equiv.) and further heating under reflux for additional 5 h. We isolated by column chromatography on silica gel compounds **4a**–**g** in high yield. Attempted one-pot sequential trifluoroacetylation of **2a**–**g** with TFAA in THF or acetonitrile under reflux followed by Beckmann rearrangement, on the other hand, led to incomplete conversion of the incipient 3-trifluoroacetyl–substituted oximes after 12 h with traces of the 3-trifluoroacetylindoleacetamides detected in the crude mixture by thin layer chromatography and ^1^H-NMR spectroscopy. We were also able to obtain single crystals of **4g** suitable for X-ray diffraction studies by slow evaporation of a DMSO solution [20]. Crystal data and structure refinement, bond lengths and torsion angles are enclosed as part of the Supplementary Material. The crystal studied was an inversion twin (Figure 2). Thus, X-ray crystallography provided an unambiguous retrospective proof that the Beckmann rearrangement of the oximes derivatives **2** occurred via aryl migration.

As a guide to 7-aminoindoles with potential biological properties, we decided to evaluate compounds **3a**–**g** and **4a**–**g** for in vitro antiplasmodial activity against the chloroquine-sensitive strain of malaria parasite *P. falciparum* (3D7).

### 2.2. Biology

#### 2.2.1. Antimalarial Activity Assay

The *P. falciparum* lactate dehydrogenase enzyme (pLDH) is considered as a potential molecular target for antimalarial agents due to this parasite’s dependence on glycolysis for energy production. The in vitro antiplasmodial activity of compounds **3a**–**g** and **4a**–**g** was measured by parasite survival using parasite lactate dehydrogenase (pLDH) assay with 100% DMSO and chloroquine as the negative and positive controls, respectively. Lactate dehydrogenase is an enzyme found in all cells that catalyzes the formation of pyruvate from lactate reducing the co-enzyme nicotinamide adenine dinucleotide (NAD) to NADH. In parasites, the NAD analogue 3-acetylpyridine adenine nucleotide (APAD) is reduced to APADH. In contrast, human red blood cell LDH carries out this reaction at a slow rate in the presence of APAD. The development of APADH is measured, and there is a correlation between levels of parasitemia and the activity of parasite LDH [21]. The IC_50_ values are expressed as the % parasite survival relative to the control, calculated from fitted sigmoidal dose-response curves and are represented in Table 1 (refer to the supporting information for detailed data). 

The selectivity index (SI) was calculated as the ratio of IC_50_ values to the minimal inhibitory concentration (MIC) values and the compound is considered selective if the selectivity index is >10 [22]. The concentration of DMSO from 100 µM–5.13 × 10^−3^ µM had no effect on the parasites (91–109% parasite survival). The antiplasmodial activity of these novel compounds is between 1400 and 14,000 nM, which is very low compared to chloroquine activity (10 nM) and they have low selectivity index which ranges between 0.13–0.66. The MICs for compounds **3a**, **3b**, **3f**, **4a**–**c** and **4e**–**g** was 11 µM while those for **3c**, **3e**, **3g** and **4d** was found to be 33 µM and **3d** was found to have MIC of >100 µM. Nevertheless, some of the test compounds **3a**–**g** and **4a**–**g** were found to exhibit moderate to significant antiplasmodial activity against the chloroquine-sensitive strain of *P. falciparum* (3D7). Within series of the 3-unsubstituted *N-*(indol-7-yl)acetamides **3a**–**g**, for example, the 2-(4-fluoro-phenyl) derivatives **3a** and **3f** substituted with 4-fluorophenyl or 4-fluorostyryl group at the 5-position showed moderate in vitro antiplasmodial activity against this chloroquine-sensitive strain of *P. falciparum* with IC_50_ values of 1.43 µM (SI = 0.13) and 2.14 µM (SI = 0.20), respectively. Compound **4a** substituted with a trifluoroacetyl group at the 3-position was found to exhibit moderate antiplasmodial activity (IC_50_ = 2.52 µM, SI = 0.23) compared to the corresponding C-3 unsubstituted precursor **3a** (IC_50_ = 1.43 µM). The presence of a 3-trifluoroacetyl group in **4f**, on the other hand, resulted in moderate activity (IC_50_ = 5.15 µM, SI = 0.47) compared to the corresponding C-3 unsubstituted precursor **3f** (IC_50_ = 2.14 µM). A combination of 2-(4-methoxyphenyl)-, 7-(4-fluorostyryl)- and 3-trifluoroacetyl groups in **4g** resulted in appreciable antiplasmodial activity (IC_50_ = 3.39 µM, SI = 0.31) compared to corresponding substrate **3g**, which showed no activity against the parasite (IC_50_ = 14.03 µM) with the selectivity index of 0.42. The observed increased activity of the 3-trifluoroacetyl derivatives **4** may be due to the electron-withdrawing effect of the trifluoromethyl group (-CF_3_) on an indole framework, which has been found to generally increase the lipophilicity, metabolic stability and activity profile compared to that of the 3-unsubstituted or 3-acetyl analogues [1]. These preliminary antiplasmodial activity results also revealed the 4-fluorophenyl or 4-fluorostyryl group as the optimal aryl substituent at the 5-position of the indole framework of compounds **3** and **4**. It has been demonstrated that a fluorine atom on the aromatic ring generally increase biological activity of the molecule due to its enhanced lipophilicity [23] and its strong polar interaction with the protein cavity [24]. We took into account that some of the most active antimalarial drugs are nephrotoxic [25] and then screened the four most active compounds **3a**, **3f**, **4a** and **4g** for toxicity against monkey kidney Vero cells. 

#### 2.2.2. Evaluation of Cytotoxicity against Vero Cells

The four compounds **3a**, **3f**, **4a** and **4g** were evaluated for their in vitro cytotoxic effects against monkey kidney Vero cells using the well-established 3-(4,5-dimethylthiazole-2-yl)-2,5-diphenyl tetrazolium bromide-based colorimetric cell viability (MTT) assay. The LC_50_ values (lethal concentration at which 50% of the cells are killed) of the four compounds (average from three independent experiments) are listed in Table 2. The LC_50_ values of the four compounds ranged from more than 100 µM to 13.78 µM. Overall, all four compounds had low toxicity at the highest concentrations tested.

The development of a new class of drugs that acts through different mechanism of action is of great importance to circumvent the increasing resistance of *P. falciparum* to existing antimalarial agents. To help us understand the observed antiplasmodial activity of compounds **3** and **4** and guide further structure activity relationship (SAR) studies we conducted molecular docking of **3a**, **3f**, **4a** and **4g** against lactate dehydrogenase (pLDH).

### 2.3. Molecular Docking Studies of Compounds ***3**(**a**,**f**)* and ***4**(**a**,**g**)*

**Figure 3:**
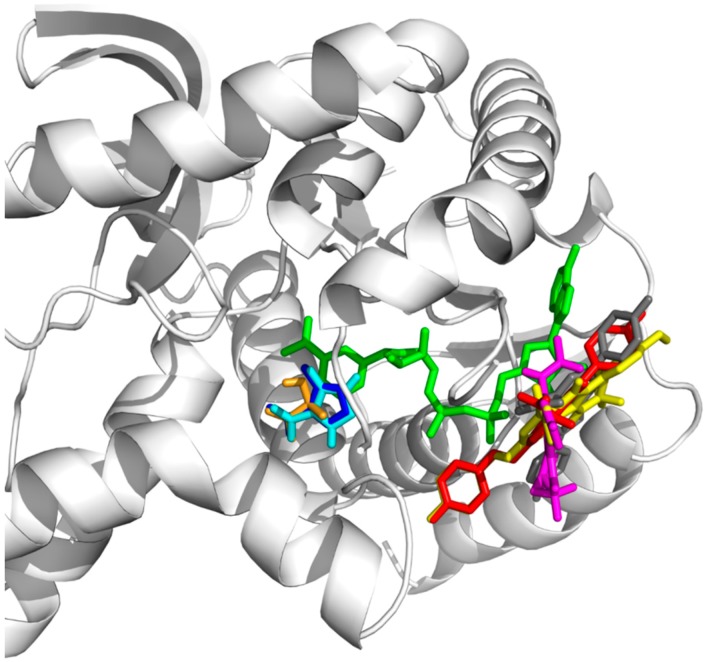
The conformation of OXQ crystal structure (dark blue stick presentation), docked OXQ (light blue stick presentation), lactate (brown stick presentation) and test compound **3a**, **3f**, **4a** and **4g** (grey, red, pink and yellow stick presentation, respectively) in parasite lactase dehydrogenase (pLDH). Green stick is the NAD+ as the co-factor of pLDH.

4-Hydroxy-1,2,5-oxazole-3-carboxylic acid (OXQ) is the inhibitor for pLDH and co-crystalized with pLDH [26]. The control docking of OXQ has root mean square deviation (RMSD) of 0.4 Å (Table 3) with the crystal structure showing the appropriateness of the parameters used in this docking simulation. Table 3 also shows that the calculated binding free energy for OXQ and lactate (the substrate pLDH) was −7.11 and −5.58 kcal/mol, respectively. This could be due to the fact that OXQ formed 9 hydrogen bonds, but lactate has only 7 hydrogen bond interaction with pLDH/NAD+ (Table 3). In addition, lactate was docked at the same binding site with OXQ, which was near to the nicotinamide region of NAD+ (Figure 3). This has further demonstrated that OXQ is able to compete with lactate for the binding site and thus inhibit pLDH. However, compounds **3**(**a**,**f**) and **4**(**a**,**g**) were docked near to the adenine region of NAD+ (Figure 3). The binding free energy of **3f** and **4**(**a,g**) were comparable (~−6.4 kcal/mol) while **3a** has the weakest binding free energy (−5.95 kcal/mol) (Table 3). Two hydrogen bonds were detected in **3**(**a**,**f**) while **4**(**a**,**g**) have only one hydrogen bond. NAD+ is the co-factor of pLDH and the nicotinamide region was buried deep into the core of pLDH with relatively narrow channel. Crystal structure of the complex between chloroquine and *P. falciparum* lactate dehydrogenase has previously shown this drug within the NADH binding pocket of the enzyme occupying a position similar to that of the adenyl ring of the cofactor [27]. Molecular docking of chloroquine and quinoline-based derivatives into the glycolytic enzyme lactate dehydrogenase, on the other hand, showed that these compounds only bind to this site in the absence of the bound NADH during docking and prefer adenine region of NAD+ which is near to the pLDH surface [28]. Compounds **3**(**a**,**f**) and **4**(**a**,**g**) were also unable to access the narrow channel of the NAD+ binding site and resulted to be docked at the adenine region of NAD+ which is near to the pLDH surface. Compound **3a** is the only test compound to form hydrogen bonding with NAD+ but other test compounds interacted and formed hydrogen bonding with pLDH. It is also noted that both fluorophenyl ring ends of **3a**, **3f** and **4g** were in parallel with the phosphate region to adenine region of NAD+. Therefore, ring stacking interactions of fluorophenyl ring in **3**(**a**,**f**) and **4g** with adenine region of NAD+ was noticed. However, only the acetyl region of compound **4a** was positioned towards the adenine region of NAD+ with other region pointed away from NAD+.

## 3. Experimental

### 3.1. General Information

Melting points were recorded on a Thermocouple digital melting point apparatus (Stuart, Staffordshire, UK) and are uncorrected. IR spectra were recorded as powders using a Bruker VERTEX 70 FT-IR Spectrometer (Bruker Optics, Billerica, MA, USA) with a diamond ATR (attenuated total reflectance) accessory by using the thin-film method. For column chromatography, Merck kieselgel 60 (0.063–0.200 mm) (Merck KGaA, Frankfurt, Germany) was used as stationary phase. NMR spectra were obtained as DMSO-*d*_6_ solutions using Varian 300 MHz NMR spectrometer (Varian Inc., Palo Alto, CA, USA) and the chemical shifts are quoted relative to the TMS peak. Low- and high-resolution mass spectra were recorded using Waters Synapt G2 Quadrupole Time-of-flight mass spectrometer (Waters Corp., Milford, MA, USA) at the University of Stellenbosch Mass Spectrometry Unit. The synthesis and characterization of compounds **1a**–**g** have been described before [11].

### 3.2. Typical Procedure for the Synthesis of Oxime Derivatives ***2a**–**g***

A stirred mixture of **1** (1 equivalent) and hydroxylamine hydrochloride (1.5 equivalent) and pyridine (1.5 equivalent) in ethanol (10 mL/mmol of **1**) was heated at 80 °C overnight. The mixture was cooled to room temperature and quenched with an ice-cold water and the product was extracted into chloroform. The combined organic phases were washed with water and dried over anhyd. MgSO_4_. The salt was filtered off and the solvent was evaporated under reduced pressure. The residue was purified by column chromatography on silica gel (20% EtOAc–hexane as eluent) to afford the oxime derivative **2**. The following compounds were prepared in this fashion:

*1-(2,5-bis(4-Fluorophenyl)-1H-indol-7-yl) Ethanone Oxime* (**2a**). A mixture of **1a** (0.15 g, 0.43 mmol), hydroxylamine hydrochloride (0.05 g, 0.65 mmol) and pyridine (0.05 g, 0.65 mmol) in ethanol (10 mL) afforded **2a** as a solid (0.15 g, 94%), *R_f_* 0.67, mp. 238–241 °C; ν_max_ (ATR) 462, 509, 539, 612, 663, 753, 785, 813, 826, 867, 921, 990, 1099, 1159, 1221, 1232, 1262, 1290, 1373, 1433, 1469, 1514, 3215, 3422 cm^−1^; ^1^H-NMR (DMSO-*d*_6_) 2.38 (3H, s, CH_3_), 6.99 (1H, s, 3-H), 7.26 (2H, t, *J* = 8.7 Hz, 3″,5″-H), 7.33 (2H, t, *J =* 9.0 Hz, 3′,5′-H), 7.58 (1H, d, *J* = 1.2 Hz, 4-H), 7.74 (2H, dd, *J* = 5.7 and 8.7 Hz, 2″,6″-H), 7.81–7.87 (3H, m, 6-H and 2′,6′-H), 10.85 (1H, s, NH), 11.43 (1H, s, OH); ^13^C-NMR (DMSO-*d*_6_) 11.3, 100.1, 116.0 (d, ^2^*J*_CF_ = 21.8 Hz), 116.5 (d, ^2^*J*_CF_ = 21.8 Hz), 119.8, 120.3, 120.6, 127.4 (d, ^3^*J*_CF_ = 8.0 Hz), 128.6 (d, ^4^*J*_CF_ = 3.5 Hz), 129.2 (d, ^3^*J*_CF_ = 8.0 Hz), 130.2, 131.8, 133.4, 137.7, 138.3 (d, ^4^*J*_CF_ = 3.5 Hz), 155.0, 161.9 (d, ^1^*J*_CF_ = 241.7 Hz), 162.3 (d, ^1^*J*_CF_ = 243.9 Hz); HRMS (ES): MH^+^, found 363.1309. C_22_H_17_N_2_F_2_O^+^ requires: 363.1309.

*1-(5-(4-Fluorophenyl)-2-(4-methoxyphenyl)-1H-indol-7-yl) Ethanone Oxime* (**2b**). A mixture of **1b** (0.15 g, 0.42 mmol), hydroxylamine hydrochloride (0.04 g, 0.62 mmol) and pyridine (0.05 g, 0.62 mmol) in ethanol (10 mL) afforded **2b** as a solid (0.14 g, 87%), *R_f_* 0.47, mp. 212–214 °C; ν_max_ (ATR) 433, 518, 541, 554, 615, 664, 692, 752, 784, 827, 921, 986, 1021, 1158, 1182, 1217, 1240, 1315, 1371, 1436, 1464, 1513, 3226, 3405 cm^−1^; ^1^H-NMR (DMSO-*d*_6_) 2.39 (3H, s, CH_3_), 3.80 (3H, s, CH_3_), 6.88 (1H, s, 3-H), 7.05 (2H, d, *J* = 8.7 Hz, 3′,5′-H), 7.27 (2H, t, *J* = 8.7 Hz, 3″,5″-H), 7.57 (1H, d, *J* = 1.2 Hz, 4-H), 7.72–7.89 (5H, m, 6-H, 2′,6′-H and 2″,6″-H), 10.76 (1H, s, NH), 11.43 (1H, s, OH); ^13^C-NMR (DMSO-*d*_6_) 11.3, 55.7, 98.8, 115.0, 116.0 (d, ^2^*J*_CF_ = 20.6 Hz), 119.5, 119.9, 120.2, 124.6, 126.7, 129.1 (d, ^3^*J*_CF_ = 8.0 Hz), 130.4, 131.6, 133.1, 138.4 (d, ^4^*J*_CF_ = 3.4 Hz), 138.7, 155.1, 159.6, 161.8 (d, ^1^*J*_CF_ = 241.7 Hz); HRMS (ES): MH^+^, found: 375.1507. C_23_H_20_N_2_FO_2_^+^ requires: 375.1509.

*1-(2-(4-Fluorophenyl)-5-(4-methoxyphenyl)-1H-indol-7-yl)ethanone Oxime* (**2c**). A mixture of **1c** (0.15 g, 0.43 mmol), hydroxylamine hydrochloride (0.04 g, 0.62 mmol) and pyridine (0.05 g, 0.62 mmol) in ethanol (10 mL) afforded **2c** as a solid (0.14 g, 88%), *R_f_* 0.58, mp. 219–222 °C; ν_max_ (ATR) 512, 561, 613, 664, 692, 753, 789, 826, 921, 989, 1023, 1099, 1156, 1182, 1240, 1272, 1371, 1427, 1466, 1520, 3369, 3406 cm^−1^; ^1^H-NMR (DMSO-*d*_6_) 2.37 (3H, s, CH_3_), 3.77 (3H, s, OCH_3_), 6.96 (1H, s, 3-H), 7.00 (2H, d, *J* = 8.7 Hz, 3″,5″-H), 7.33 (2H, t, *J* = 8.7 Hz, 3′,5′-H), 7.56 (1H, d, *J* = 1.2 Hz, 4-H), 7.63 (2H, d, *J* = 8.1 Hz, 2″,6″-H), 7.76 (1H, d, *J* = 1.2 Hz, 6-H), 7.84 (2H, d, *J* = 8.7 Hz, 2′,6′-H), 10.81 (1H, s, NH), 11.41 (1H, s, OH); ^13^C-NMR (DMSO-*d*_6_) 11.3, 55.6, 100.1, 114.7, 116.5 (d, ^2^*J*_CF_ = 21.8 Hz), 119.2, 120.1, 120.5, 127.3 (d, ^3^*J*_CF_ = 9.2 Hz), 128.3, 128.7 (d, ^4^*J*_CF_ = 3.5 Hz), 130.2, 132.5, 133.1, 134.2, 137.5, 155.0, 158.7, 162.2 (d, ^1^*J*_CF_ = 242.8 Hz); HRMS (ES): MH^+^, found: 375.1509. C_23_H_20_N_2_FO_2_^+^ requires: 375.1509.

*1-(2,5-Bis(4-methoxyphenyl)-1H-indol-7-yl)ethanone Oxime* (**2d**). A mixture of **1d** (0.15 g, 0.40 mmol), hydroxylamine hydrochloride (0.04 g, 0.61 mmol) and pyridine (0.05 g, 0.61 mmol) in ethanol (10 mL) afforded **2d** as a solid (0.14 g, 88%), *R_f_* 0.38, mp. 209–212 °C; ν_max_ (ATR) 545, 615, 663, 752, 784, 818, 828, 838, 921, 986, 1028, 1114, 1176, 1236, 1370, 1434, 1466, 1516, 3390, 3416 cm^−1^; ^1^H-NMR (DMSO-*d*_6_) 2.37 (3H, s, CH_3_), 3.78 (3H, s, OCH_3_), 3.80 (3H, s, OCH_3_), 6.87 (1H, d, s, 3-H), 7.00 (2H, d, *J* = 8.7 Hz, 3″,5″-H), 7.05 (2H, d, *J* = 8.7 Hz, 3′,5′-H), 7.53 (1H, d, *J* = 1.2 Hz, 4-H), 7.63 (2H, d, *J* = 8.7 Hz, 2″,6″-H), 7.72 (2H, d, *J* = 8.7 Hz, 2′,6′-H), 7.74 (1H, d, *J* = 1.2 Hz, 6-H), 10.71 (1H, s, NH), 11.40 (1H, s, OH); ^13^C-NMR (DMSO-*d*_6_) 11.2, 55.6, 55.7, 98.7, 114.7, 115.0, 119.0, 119.8, 120.0, 124.7, 126.6, 128.3, 130.4, 132.4, 134.4, 138.5, 155.1, 158.7, 159.5; HRMS (ES): MH^+^, found: 387.1714. C_24_H_23_N_2_O_3_^+^ requires: 387.1709.

*1-(5-(4-Fluorophenyl)-2-phenyl)-1H-indol-7-yl)ethanone Oxime* (**2e**). A mixture of **1e** (0.15 g, 0.42 mmol), hydroxylamine hydrochloride (0.04 g, 0.63 mmol) and pyridine (0.05 g, 0.63 mmol) in ethanol (10 mL) afforded **2e** as a solid (0.12 g, 76%), *R_f_* 0.58, mp. 197–199 °C; ν_max_ (ATR) 484, 508, 533, 605, 684, 735, 785, 841, 885, 952, 989, 1027, 1137, 1153, 1228, 1274, 1360, 1376, 1449, 1507, 3218, 3412 cm^−1^; ^1^H-NMR (DMSO-*d*_6_) 2.39 (3H, s, CH_3_), 6.98 (1H, d, s, 3-H), 7.19 (2H, t, *J* = 8.7 Hz, 3″,5″-H), 7.27–7.37 (3H, m, 2x CH=C and 4′-H), 7.48 (2H, t, *J* = 7.5 Hz, 3′,5′-H), 7.63 (2H, d, *J* = 8.7 Hz, 2″,6″-H), 7.69 (1H, d, *J* = 1.2 Hz, 4-H), 7.78–7.80 (3H, m, 6-H and 2′,6′-H), 10.89 (1H, s, NH), 11.46 (1H, s, OH); ^13^C-NMR (DMSO-*d*_6_) 11.2, 100.0, 116.0 (d, ^2^*J*_CF_ = 21.8 Hz), 120.0, 120.1, 120.2, 125.2 (2 × C), 128.3, 128.4 (d, ^3^*J*_CF_ = 8.0 Hz), 129.4, 129.5, 129.9, 130.0, 131.9, 133.7, 134.7 (d, ^4^*J*_CF_ = 2.3 Hz), 138.4, 155.0, 161.8 (d, ^1^*J*_CF_ = 242.8 Hz); HRMS (ES): MH^+^, found: 371.1568. C_23_H_20_N_2_FO_2_^+^ requires: 371.1560.

*1-(2-(4-Fluorophenyl)-5-(4-fluorostyryl)-1H-indol-7-yl)ethanone Oxime* (**2f**). A mixture of **1f** (0.15 g, 0.40 mmol), hydroxylamine hydrochloride (0.04 g, 0.60 mmol) and pyridine (0.05g, 0.60 mmol) in ethanol (10 mL) afforded **2f** as a solid (0.12 g, 75%), *R_f_* 0.67, mp. 238‒240 °C; ν_max_ (ATR) 480, 509, 540, 578, 667, 718, 745, 772, 784, 805, 832, 881, 949, 988, 1097, 1154, 1223, 1286, 1376, 1431, 1467, 1505, 3234, 3409 cm^−1^; ^1^H-NMR (DMSO-*d*_6_) 2.37 (3H, s, CH_3_), 6.95 (1H, d, s, 3-H), 7.19 (2H, t, *J* = 8.7 Hz, 3″,5″-H), 7.29 (2H, d, *J* = 17.0 Hz, CH=CH), 7.31 (2H, d, *J* = 16.2 Hz, CH=CH), 7.32 (2H, t, *J* = 8.7 Hz, 3′,5′-H), 7.63 (2H, d, *J* = 8.7 Hz, 2″,6″-H), 7.67 (1H, d, *J* = 1.2 Hz, 4-H), 7.77 (1H, d, *J* = 1.2 Hz, 6-H), 7.83 (2H, d, *J* = 8.7 Hz, 2′,6′-H), 10.85 (1H, s, NH), 11.42 (1H, s, OH); ^13^C-NMR (DMSO-*d*_6_) 11.3, 100.0, 116.0 (d, ^2^*J*_CF_ 21.8 Hz), 116.5 (d, ^2^*J*_CF_ = 21.8 Hz), 120.1 (2 × C), 120.2, 125.2, 127.3 (d, ^3^*J*_CF_ = 8.0 Hz), 128.3 (d, ^3^*J*_CF_ = 8.0 Hz), 128.6 (d, ^4^*J*_CF_ = 3.4 Hz), 129.5, 129.9, 130.0, 133.7, 134.7 (d, ^4^*J*_CF_ = 3.5 Hz), 137.6, 154.9, 161.8 (d, ^1^*J*_CF_ = 242.8 Hz), 162.2 (d, ^1^*J*_CF_ = 243.9 Hz); HRMS (ES): MH^+^, found: 389.1465. C_24_H_19_N_2_F_2_O^+^ requires: 389.1465.

*1-(5-(4-Fluorostyryl)-2-(4-methoxyphenyl)-1H-indol-7-yl)ethanone Oxime* (**2g**). A mixture of **1f** (0.15 g, 0.39 mmol), hydroxylamine hydrochloride (0.04 g, 0.59 mmol) and pyridine (0.05 g, 0.59 mmol) in ethanol (10 mL) afforded **2f** as a solid (0.11 g, 76%), *R_f_* 0.47, mp. 220–222 °C; ν_max_ (ATR) 462, 507, 584, 606, 666, 715, 745, 786, 830, 838, 881, 960, 989, 1020, 1179, 1224, 1249, 1355, 1435, 1467, 1499, 3423, 3461 cm^−1^; ^1^H-NMR (DMSO-*d*_6_) 2.37 (3H, s, CH_3_), 3.80 (3H, s, OCH_3_), 6.85 (1H, s, 3-H), 7.04 (2H, d, *J* = 8.7 Hz, 3′,5′-H), 7.19 (2H, t, *J* = 8.7 Hz, 3″,5″-H), 7.23 (2H, d, *J* = 16.5 Hz, CH=CH), 7.32 (2H, d, *J* = 16.5 Hz, CH=CH), 7.61‒7.66 (3H, m, 4-H and 2″,6″-H), 7.71 (2H, d, *J* = 8.7 Hz, 2′,6′-H), 7.75 (1H, d, *J* = 1.2 Hz, 6-H), 10.75 (1H, s, NH), 11.41 (1H, s, OH); ^13^C-NMR (DMSO-*d*_6_) 11.3, 55.7, 98.7, 115.0 (2 × C), 116.0 (d, ^2^*J*_CF_ = 21.8 Hz), 119.8, 119.9, 124.6, 125.0, 126.6, 128.3 (d, ^3^*J*_CF_ = 8.0 Hz), 129.3, 130.0, 130.2, 133.5, 134.7 (d, ^4^*J*_CF_ = 3.4 Hz), 138.6, 155.0, 159.6, 161.8 (d, ^1^*J*_CF_ = 241.6 Hz); HRMS (ES): MH^+^, found: 401.1664. C_25_H_22_N_2_FO_2_^+^ requires: 401.1665.

### 3.3. Typical Procedure for the Beckmann Rearrangement of ***3a**–**g***

A stirred mixture of **2** (1 equivalent) and TFA (1 equivalent) in acetonitrile (20 mL/mmol of **2**) was heated at 80 °C for 2 h. The mixture was cooled to room temperature and quenched with ice-cold water. The product was extracted with chloroform (3 × 20 mL) and the combined organic layers were dried over anhydrous MgSO_4_ and the salt was filtered off. The solvent was evaporated under reduced pressure and the residue was purified by column chromatography on a silica gel (60% EtOAc–hexane as eluent) to afford **3** as a solid. Products **3a**–**f** were prepared in this fashion:

*N-(2,5-Bis(4-fluorophenyl)-1H-indol-7-yl) Acetamide* (**3a**). A mixture of **2a** (0.10 g, 0.28 mmol) and TFA (0.03 g, 0.28 mmol) in acetonitrile (5 mL) afforded **3a** as solid (0.08 g, 80%); *R_f_* 0.51, mp. 272–275 °C; ν_max_ (ATR) 459, 509, 538, 595, 653, 753, 795, 810, 828, 1010, 1102, 1159, 1218, 1230, 1280, 1433, 1474, 1513, 1549, 1630, 1653, 3243, 3339 cm^−1^; ^1^H-NMR (DMSO-*d*_6_) 2.19 (3H, s, CH_3_), 6.92 (1H, s, 3-H), 7.25 (2H, t, *J* = 8.7 Hz, 3′,5′-H), 7.34 (2H, t, *J* = 8.7 Hz, 3″,5″-H), 7.53 (1H, d, *J* = 1.2 Hz, 4-H), 7.63 (2H, d, *J* = 8.7 Hz, 2″,6″-H), 7.85–7.90 (3H, m, 6-H and 2′,6′-H), 9.73 (1H, s, NH), 11.10 (1H, s, NH); ^13^C-NMR (DMSO-*d*_6_) 24.5, 100.3, 113.3, 114.6, 116.0 (d, ^2^*J*_CF_ = 21.8 Hz), 116.1, 116.4 (d, ^2^*J*_CF_ = 20.6 Hz), 124.4, 127.6 (d, ^3^*J*_CF_ = 8.0 Hz), 128.8 (d, ^4^*J*_CF_ = 3.4 Hz), 128.9 (d, ^3^*J*_CF_ = 8.0 Hz), 130.9, 131.8, 137.6, 138.5 (d, ^4^*J*_CF_ = 2.3 Hz), 161.8 (d, ^1^*J*_CF_ = 241.6 Hz), 162.2 (d, ^1^*J*_CF_ = 242.8 Hz), 169.0; HRMS (ES): MH^+^, found: 363.1311. C_22_H_17_N_2_F_2_O^+^ requires: 363.1231.

*N-(5-(4-Fluorophenyl)-2-(4-methoxyphenyl)-1H-indol-7-yl) Acetamide* (**3b**). A mixture of **2b** (0.10 g, 0.27 mmol) and TFA (0.03 g, 0.27 mmol) in acetonitrile (5 mL) afforded **3b** as a solid (0.08 g, 75%); *R_f_* 0.37, mp. 213–215 °C; ν_max_ (ATR) 518, 533, 595, 745, 774, 811, 831, 1025, 1101, 1177, 1226, 1253, 1367, 1390, 1426, 1472, 1516, 1553, 1609, 1654, 3316 cm^−1^; ^1^H-NMR (DMSO-*d*_6_) 2.20 (3H, s, CH_3_), 3.80 (3H, s, OCH_3_), 6.80 (1H, s, 3-H), 7.06 (2H, d, *J* = 8.7 Hz, 3′,5′-H), 7.25 (2H, t, *J* = 8.7 Hz, 3″,5″-H), 7.49 (1H, d, *J* = 1.2 Hz, 4-H), 7.62 (2H, t, = 8.7 Hz, 2″,6″-H), 7.76 (2H, d, *J* = 8.7 Hz 2′,6′-H), 7.87 (1H, d, *J* = 1.2 Hz, 6-H), 9.72 (1H, s, NH), 11.01 (1H, s, NH); ^13^C-NMR (DMSO-*d*_6_) 24.5, 55.7, 99.0, 112.7, 114.2, 114.9, 116.0 (d, ^2^*J*_CF_ 20.6 Hz), 124.3, 124.9, 126.9, 128.4, 128.8 (d, ^3^*J*_CF_ 8.0 Hz), 131.1, 131.6, 138.6 (d, ^4^*J*_CF_ 3.5 Hz), 159.5, 161.7 (d, ^1^*J*_CF_ 241.7 Hz), 169.0; HRMS (ES): MH^+^, found: 375.1510. C_23_H_20_N_2_FO_2_^+^ requires: 375.1509.

*N-(2-(4-Fluorophenyl)-5-(4-methoxyphenyl)-1H-indol-7-yl) Acetamide* (**3c**). A mixture of **2c** (0.10 g, 0.27 mmol) and TFA (0.03 g, 0.27 mmol) in acetonitrile (5 mL) afforded **3c** as a solid (0.07 g, 73%); *R_f_* 0.47, mp. 216–218 °C; ν_max_ (ATR) 505, 523, 550, 600, 719, 749, 786, 805, 826, 1032, 1159, 1178, 1231, 1371, 1427, 1480, 1516, 1566, 1631, 1654, 3258, 3320 cm^−1^; ^1^H-NMR (DMSO-*d*_6_) 2.19 (3H, s, CH_3_), 3.77 (3H, s, OCH_3_), 6.90 (1H, s, 3-H), 6.99 (2H, d, *J* = 8.4 Hz, 3″,5″-H), 7.34 (2H, t, *J* = 8.7 Hz, 3′,5′-H), 7.48 (1H, d, *J* = 1.2 Hz, 4-H), 7.53 (2H, t, *J* = 8.4 Hz, 2″,6″-H), 7.84–7.89 (3H, m, 6-H and 2′,6′-H), 9.71 (1H, s, NH), 11.04 (1H, s, NH); ^13^C-NMR (DMSO-*d*_6_) 24.5, 55.6, 100.2, 113.2, 114.0, 114.7, 116.4 (d, ^2^*J*_CF_ = 20.6 Hz), 124.3, 127.5 (d, ^3^*J*_CF_ = 8.0 Hz), 128.1, 128.5, 129.1 (d, ^4^*J*_CF_ = 3.5 Hz), 130.9, 132.6, 134.5, 137.4, 158.6, 162.1 (d, ^1^*J*_CF_ = 242.8 Hz), 169.0; HRMS (ES): MH^+^, found: 375.1508. C_23_H_20_N_2_FO_2_^+^ requires: 375.1509.

*N-(2,5-Bis(4-methoxyphenyl)-1H-indol-7-yl) Acetamide* (**3d**). A mixture of **2d** (0.10 g, 0.26 mmol) and TFA (0.03 g, 0.26 mmol) in acetonitrile (5 mL) afforded **3d** as a solid (0.07 g, 70%); *R_f_* 0.31, mp. 236–239 °C; ν_max_ (ATR) 529, 564, 600, 723, 775, 830, 1030, 1112, 1176, 1244, 1280, 1372, 1432, 1440, 1514, 1550, 1605, 1632, 1656, 3269, 3321 cm^−1^; ^1^H-NMR (DMSO-*d*_6_) 2.19 (3H, s, CH_3_), 3.77 (3H, s, OCH_3_), 3.80 (3H, s, OCH_3_), 6.79 (1H, s, 3-H), 6.99 (2H, d, *J* = 9.0 Hz, 3″,5″-H), 7.06 (2H, d, *J =* 8.1 Hz, 3′,5′-H), 7.45 (1H, d, *J* = 1.2 Hz, 4-H), 7.53 (2H, d, *J* = 8.4 Hz, 2″,6″-H), 7.76 (2H, d, *J* = 8.7 Hz 2′,6′-H), 7.83 (1H, d, *J* = 1.2 Hz, 6-H), 9.70 (1H, s, NH), 10.96 (1H, s, NH); ^13^C-NMR (DMSO-*d*_6_) 24.5, 55.6, 55.7, 98.9, 112.6, 113.7, 114.7, 114.9 (2 × C), 124.2, 125.0, 126.9, 128.0, 131.1, 132.4, 134.6, 138.4, 158.6, 159.4, 168.9; HRMS (ES): MH^+^, found: 387.1708. C_24_H_23_N_2_O_3_^+^ requires: 387.1709.

*(E)-N-(5-(4-Fluorostyryl)-2-phenyl-1H-indol-7-yl) Acetamide* (**3e**). A mixture of **2e** (0.10 g, 0.27 mmol) and TFA (0.03 g, 0.27 mmol) in acetonitrile (5 mL) afforded **3e** as a solid (0.08 g, 78%); *R_f_* 0.51, mp. 262–264 °C; ν_max_ (ATR) 481, 512, 564, 598, 689, 738, 756, 799, 814, 847, 957, 1158, 1204, 1223, 1286, 1353, 1455, 1507, 1646, 3267, 3299 cm^−1^; ^1^H-NMR (DMSO-*d*_6_) 2.18 (3H, s, CH_3_), 6.90 (1H, s, 3-H), 7.03 (1H, d, *J* = 16.5 Hz, CH=CH), 7.16 (2H, t, *J* = 8.4 Hz, 3″,5″-H), 7.22 (1H, d, *J* = 16.5 Hz, CH=CH), 7.33–7.35 (1H, m, 4′-H), 7.45–7.48 (3H, m, 4-H and 2″,6″-H), 7.46–7.59 (2H, m, 3′,5′-H), 7.82 (2H, d, *J* = 7.8 Hz, 2′,6′-H) 7.88 (1H, d, *J* = 1.2 Hz, 6-H), 9.70 (1H, s, NH), 11.12 (1H, s, NH); ^13^C-NMR (DMSO-*d*_6_) 24.5, 100.2, 112.4, 115.7, 115.9 (d, ^2^*J*_CF_ = 20.6 Hz), 124.3, 124.7, 125.5, 128.3, 128.2 (d, ^3^*J*_CF_ = 8.0 Hz), 128.4, 129.3, 129.4, 129.5, 130.4, 130.5, 132.3, 134.6 (d, ^4^*J*_CF_ = 2.3 Hz), 138.4, 161.7 (d, ^1^*J*_CF_ = 242.8 Hz), 170.0; HRMS (ES): MH^+^, found: 371.1554. C_24_H_20_N_2_FO^+^ requires: 371.1560.

*(E)-N-(2-(4-Fluorophenyl)-5-(4-fluorostyryl)-1H-indol-7-yl) Acetamide* (**3f**). A mixture of **2f** (0.10 g, 0.28 mmol) and TFA (0.03 g, 0.28 mmol) in acetonitrile (5 mL) afforded **3f** as a solid (0.08 g, 77%), *R_f_* 0.51, mp. 278–280 °C; ν_max_ (ATR) 467, 509, 552, 581, 609, 761, 789, 831, 848, 957, 1012, 1099, 1159, 1221, 1232, 1352, 1441, 1477, 1502, 1545, 1652, 3257, 3301 cm^−1^; ^1^H-NMR (DMSO-*d*_6_) 2.19 (3H, s, CH_3_), 6.88 (1H, s, 3-H), 7.04 (2H, d, *J* = 16.5 Hz, CH=CH), 7.18 (2H, t, *J* = 8.7 Hz, 3′,5′-H), 7.23 (2H, d, *J* = 16.5 Hz, CH=CH), 7.34 (2H, t, *J* = 8.7 Hz, 3″,5″-H), 7.49 (1H, d, *J* = 1.2 Hz, 4-H), 7.63 (2H, t, *J* = 8.7 Hz, 2″,6″-H), 7.84–7.89 (3H, m, 6-H and 2′,6′-H), 9.68 (1H, s, NH), 11.08 (1H, s, NH); ^13^C-NMR (DMSO-*d*_6_) 24.4, 100.2, 110.0, 112.7, 115.9 (d, ^2^*J*_CF_ = 21.8 Hz), 116.4 (d, ^2^*J*_CF_ = 20.6 Hz), 124.2, 124.7, 127.6 (d, ^3^*J*_CF_ = 9.1 Hz), 128.3 (d, ^3^*J*_CF_ = 8.0 Hz), 128.9 (d, ^4^*J*_CF_ = 2.3 Hz), 129.5, 129.6, 130.3, 130.6, 134.6 (d, ^4^*J*_CF_ = 2.3 Hz), 137.5, 161.7 (d, ^1^*J*_CF_ = 241.6 Hz), 162.2 (d, ^1^*J*_CF_ = 243.9 Hz), 169.0; HRMS (ES): MH^+^, found: 389.1465. C_24_H_19_N_2_F_2_O^+^ requires: 389.1465.

*(E)-N-(5-(4-Fluorostyryl)-2-(4-methoxyphenyl)-1H-indol-7-yl) Acetamide* (**3g**). A mixture of **2g** (0.10 g, 0.25 mmol) and TFA (0.03 g, 0.25 mmol) in acetonitrile (5 mL) afforded **3g** as a solid (0.07 g, 69%); *R_f_* 0.33, mp. 233–235 °C; ν_max_ (ATR) 488, 516, 549, 584, 614, 746, 786, 809, 830, 843, 954, 1025, 1095, 1159, 1181, 1223, 1253, 1276, 1346, 1371, 1460, 1502, 1554, 1609, 1646, 3298 cm^−1^; ^1^H-NMR (DMSO-*d*_6_) 2.18 (3H, s, CH_3_), 3.80 (3H, s, OCH_3_), 6.77 (1H, d, s, 3-H), 7.02 (1H, d, *J* = 15.9 Hz, CH=CH) 7.05 (2H, d, *J* = 8.1 Hz, 3′,5′-H), 7.17 (2H, t, *J* = 8.7 Hz, 3″,5″-H), 7.22 (2H, d, *J* = 15.9 Hz, CH=CH), 7.45 (1H, d, *J* = 1.2 Hz, 4-H), 7.62 (2H, d, *J* = 8.7 Hz, 2″,6″-H), 7.75 (2H, d, *J* = 8.7 Hz, 2′,6′-H), 7.82 (1H, d, *J* = 1.2 Hz, 6-H), 9.66 (1H, s, NH), 10.99 (1H, s, NH); ^13^C-NMR (DMSO-*d*_6_) 24.5, 55.7, 98.9, 112.1, 114.9, 115.4, 115.9 (d, ^2^*J*_CF_ = 21.8 Hz), 124.1, 124.6, 124.9, 126.9, 128.3 (d, ^3^*J*_CF_ = 8.0 Hz), 129.1, 129.3, 130.5 130.8, 134.6 (d, ^4^*J*_CF_ = 3.4 Hz), 138.6, 159.5, 161.7 (d, ^1^*J*_CF_ = 242.8 Hz), 170.0; HRMS (ES): MH^+^, found: 401.1658. C_25_H_22_N_2_FO_2_^+^ requires: 401.1665.

### 3.4. Typical Procedure for the Trifluoroacetylation of ***3a**–**g***

A mixture of **3** (1 equivalent) and TFAA (1.5 equivalent) in THF (25 mL/mmol of **3**) was heated at 60 °C for 5 h. The mixture was cooled to room temperature quenched with saturated sodium hydrogen carbonate solution. The mixture was extracted with chloroform (3 × 20 mL) and the combined organic layers were dried with anhydrous MgSO_4_ and the salt was filtered off. The solvent was evaporated under reduced pressure and the residue was purified by column chromatography on a silica gel (60% EtOAc–hexane as eluent) to afford **4** as a solid. Compounds **4a**–**g** were prepared in this fashion.

*N-(2,5-Bis(4-fluorophenyl)-3-(2,2,2-trifluoroacetyl)-1H-indol-7-yl) Acetamide* (**4a**). A mixture of **3a** (0.15 g, 0.41 mmol) and TFAA (0.13 g, 0.62 mmol) in THF (10 mL) afforded **4a** as a solid (0.15 g, 79%), *R_f_* 0.51, mp. 215–218 °C; ν_max_ (ATR) 436, 514, 565, 589, 644, 721, 744, 814, 832, 900, 927, 1013, 1041, 1098, 1143, 1186, 1202, 1225, 1374, 1436, 1513, 1605, 1634, 1653, 3251, 3312 cm^−1^; ^1^H-NMR (DMSO-*d*_6_) 2.13 (3H, s, CH_3_), 7.30 (2H, t, *J* = 8.7 Hz, 3″,5″-H), 7.40 (2H, t, *J* = 8.7 Hz, 3′,5′-H), 7.61–7.72 (4H, m, 2′,6′-H and 2″,6″-H), 7.91 (1H, d, *J* = 1.2 Hz, 4-H), 8.02 (1H, d, *J* = 1.2 Hz, 6-H), 9.83 (1H, s, NH), 12.47 (1H, s, NH); ^13^C-NMR (DMSO-*d*_6_) 24.2, 108 .1, 115.6 (d, ^2^*J*_CF_ = 21.8 Hz), 116.2 (d, ^2^*J*_CF_ = 21.8 Hz), 116.3, 116.7 (q, ^1^*J*_CF_ = 288.6 Hz), 125.2, 127.9 (d, ^4^*J*_CF_ = 3.5 Hz), 128.2, 128.7, 129.2 (d, ^3^*J*_CF_ = 8.0 Hz), 132.8 (d, ^3^*J*_CF_ = 8.0 Hz), 135.6, 137.9 (d, ^4^*J*_CF_ = 2.3 Hz), 148.2, 162.2 (d, ^1^*J*_CF_ = 242.8 Hz), 163.6 (d, ^1^*J*_CF_ = 246.2 Hz), 169.2, 170.8, 175.7 (q, ^2^*J*_CF_ = 34.4 Hz); ^19^F-NMR (DMSO-*d_6_*) -115.9 (1F, ddd, *J* = 5.9, 8.7 and 14.7 Hz, 4′′-CF), −110.5 (1F, ddd, *J* = 4.8, 8.7 and 13.8 Hz, 4′-CF), −71.2 (3F, s, COCF_3_); HRMS (ES): MH^+^, found 459.1131. C_24_H_16_ N_2_F_5_O_2_^+^ requires: 459.1132.

*N-(5-(4-Fluorophenyl)-2-(4-methoxyphenyl)-3-(2,2,2-trifluoroacetyl)-1H-indol-7-yl) Acetamide* (**4b**). A mixture of **3b** (0.15 g, 0.40 mmol) and TFAA (0.13 g, 0.60 mmol) in THF (10 mL) afforded **4b** as a solid (0.15 g, 86%), *R_f_* 0.34, mp. 222–224 °C; ν_max_ (ATR) 417, 517, 539, 569, 594, 713, 722, 834, 912, 963, 1014, 1038, 1144, 1180, 1206, 1249, 1291, 1435, 1473, 1513, 1612, 1634, 1654, 1676, 3209, 3276 cm^−1^; ^1^H-NMR (DMSO-*d*_6_) 2.13 (3H, s, CH_3_), 3.84 (3H, s, OCH_3_), 7.11 (2H, d, *J* = 8.4 Hz, 3′,5′-H), 7.30 (2H, t, *J* = 8.7 Hz, 3″,5″-H), 7.57 (2H, d, *J* = 8.4 Hz, 2′,6′-H), 7.63 (2H, t, *J* = 8.7 Hz, 2″,6″-H), 7.91 (1H, d, *J* = 1.2 Hz, 4-H), 7.99 (1H, d, *J* = 1.2 Hz, 6-H), 9.80 (1H, s, NH), 12.33 (1H, s, NH); ^13^C-NMR (DMSO-*d*_6_) 24.2, 55.8, 107.7, 114.1 (2 × C), 115.0, 115.9, 116.2 (d, ^2^*J*_CF_ = 20.6 Hz), 116.8 (q, ^1^*J*_CF_ *J* = 288.5 Hz), 123.7, 125.1, 127.9, 129.0, 129.2 (d, ^3^*J*_CF_ = 8.0 Hz), 131.8, 135.4, 138.0 (d, ^4^*J*_CF_ = 3.4 Hz), 149.4, 161.1, 162.1 (d, ^1^*J*_CF_ = 242.8 Hz), 169.2, 176.0 (q, ^2^*J*_CF =_ 35.5 Hz); ^19^F-NMR (DMSO-*d*_6_) −116.0 (1F, ddd, *J* = 5.9, 9.0 and 14.9 Hz, 4″-CF), −71.0 (3F, s, COCF_3_); HRMS (ES): MH^+^, found: 471.1333. C_25_H_19_N_2_F_4_O_3_^+^ requires: 471.1332.

*N-(3-(2,2,2-Trifluoroacetyl)-2-(4-fluorophenyl)-5-(4-methoxyphenyl)-1H-indol-7-yl) Acetamide* (**4c**). A mixture of **3c** (0.15 g, 0.40 mmol) and TFAA (0.13 g, 0.60 mmol) in THF (10 mL) afforded **4c** as a solid (0.17 g, 89%), *R_f_* 0.42, mp. 255–257 °C; ν_max_ (ATR) 448, 504, 517, 545, 592, 624, 643, 729, 783, 808, 832, 850, 901, 929, 1019, 1123, 1157, 1180, 1219, 1244, 1302, 1369, 1448, 1515, 1566, 1604, 1632, 1657, 3185, 3245 cm^−1^; ^1^H-NMR (DMSO-*d*_6_) 2.11 (3H, s, CH_3_), 3.78 (3H, s, OCH_3_), 7.04 (2H, d, *J* = 7.5 Hz, 3″,5″-H), 7.39 (2H, t, *J* = 7.5 Hz, 3′,5′-H), 7.55 (2H, d, *J* = 7.5 Hz, 2″,6″-H), 7.65–7.70 (2H, m, 2′,6′-H), 7.87 (1H, d, *J* = 1.2 Hz, 4-H), 7.99 (1H, d, *J* = 1.2 Hz, 6-H), 9.84 (1H, s, NH), 12.44 (1H, s, NH); ^13^C-NMR (DMSO-*d*_6_) 24.1, 55.6, 108.0, 114.5, 114.9, 115.6 (d, ^2^*J*_CF_ = 21.8 Hz), 116.1, 116.8 (q, ^1^*J*_CF_ = 288.5 Hz), 125.2, 127.9, 128.0 (d, ^4^*J*_CF_ = 3.5 Hz), 128.3, 128.7, 132.8 (d, ^3^*J*_CF_ = 9.1 Hz), 133.8, 136.4, 148.1, 159.2, 163.5 (d, ^1^*J*_CF_ = 246.2 Hz), 169.3, 175.7 (q, ^2^*J*_CF_ 35.5 Hz); ^19^F-NMR (282 MHz, DMSO-*d_6_*) −111.9 (1F, ddd, *J* 4.8, 8.7 and 14.7 Hz, 4′ -CF), −71.2 (3F, s, COCF_3_); HRMS (ES): MH^+^, found 471.1334. C_25_H_19_N_2_F_4_O_3_^+^ requires: 471.1332.

*N-(2,5-Bis(4-methoxyphenyl)-3-(2,2,2-trifluoroacetyl)-1H-indol-7-yl) Acetamide* (**4d**). A mixture of **3d** (0.15 g, 0.39 mmol) and TFAA (0.12 g, 0.58 mmol) in THF (10 mL) afforded **4b** as a solid (0.17 g, 90%), *R_f_* 0.28, mp. 254–257 °C; ν_max_ (ATR) 528, 545, 594, 643, 714, 729, 798, 852, 900, 928, 966, 1026, 1137, 1178, 1201, 1235, 1253, 1295, 1436, 1450, 1516, 1566, 1607, 1655, 1673, 3171, 3251 cm^−1^; ^1^H-NMR (DMSO-*d*_6_) 2.13 (3H, s, CH_3_), 3.79 (3H, s, OCH_3_), 3.85 (3H, s, OCH_3_), 7.05 (2H, d, *J* = 8.7 Hz, 3″,5″-H), 7.12 (2H, d, *J* = 8.7 Hz, 3′,5′-H), 7.54 (2H, d, *J* = 8.4 Hz, 2″,6″-H), 7.57 (2H, d, *J* = 8.4 Hz, 2′,6′-H), 7.88 (1H, d, *J* = 1.2 Hz, 4-H), 7.98 (1H, d, *J* = 1.2 Hz, 6-H), 9.78 (1H, s, NH), 12.28 (1H, s, NH); ^13^C-NMR (DMSO-*d*_6_) 24.2, 55.6, 55.8, 107.6, 114.1, 114.5, 114.9, 115.8, 116.8 (q, ^1^*J*_CF_ = 288.6 Hz), 123.7, 125.0, 127.7, 128.1, 128.3, 129.0, 131.8, 133.9, 136.2, 149.2, 159.2, 161.1, 169.2, 175.9 (q, ^2^*J*_CF =_ 34.4 Hz); ^19^F-NMR (DMSO-*d_6_*) −71.0 (3F, s, COCF_3_); HRMS (ES): MH^+^, found: 483.1533. C_26_H_22_N_2_F_3_O_4_^+^ requires: 483.1532.

*(E)-N-[5,[5-(4-Fluorostyryl)-3-(2,2,2-trifluoroacetyl)-2-phenyl-1H-indol-7-yl] Acetamide* (**4e**). A mixture of **3e** (0.15 g, 0.41 mmol) and TFAA (0.13g, 0.61 mmol) in THF (10 mL) afforded **4e** as a solid (0.15 g, 80%), *R_f_* 0.35, mp. 230–233 °C; ν_max_ (ATR) 475, 507, 527, 599, 674, 702, 731, 750, 773, 818, 852, 915, 962, 1012, 1141, 1184, 1224, 1260, 1285, 1370, 1434, 1456, 1508, 1558, 1628, 1657, 1667, 3198, 3331 cm^−1^; ^1^H-NMR (DMSO-*d*_6_) 2.12 (3H, s, CH_3_), 7.13 (1H, d, *J* = 16.5 Hz, CH=CH), 7.19 (2H, t, *J* = 8.1 Hz, 3″,5″-H), 7.37 (1H, d, *J* = 16.5 Hz, CH=CH), 7.57–8.00 (5H, m, 4′-H, 3′,5′-H, and 2″,6″-H), 7.68–7.72 (2H, m, 2′,6′-H), 7.92 (1H, d, *J* = 1.2 Hz, 4-H), 8.02 (1H, d, *J* = 1.2 Hz, 6-H) 9.74 (1H, s, NH), 12.43 (1H, s, NH); ^13^C-NMR (DMSO-*d_6_*) 24.1, 107.1, 115.4, 116.0 (d, ^2^*J*_CF_ = 21.8 Hz), 116.2, 116.7 (q, ^1^*J*_CF_ = 288.5 Hz), 124.8, 125.0, 126.6, 128.6, 128.8 (d, ^3^*J*_CF_ = 8.0 Hz), 129.6, 129.7, 130.3, 130.4, 131.6, 133.3, 134.2 (d, ^4^*J*_CF_ = 2.3 Hz), 149.0, 162.0 (d, ^1^*J*_CF_ = 242.8 Hz), 169.2, 176.0 (q, ^2^*J*_CF_ = 35.5 Hz); ^19^F-NMR (DMSO-*d_6_*) −114.6 (1F, ddd, *J* = 5.9, 8.7 and 14.7 Hz, 4″-CF), −71.02 (3F, s, COCF_3_); HRMS (ES): MH^+^, found: 467.1384. C_26_H_19_N_2_F_4_O_2_^+^ requires: 467.1383.

*N-[5,[5-(4-Fluorostyryl)-3-(2,2,2-trifluoroacetyl)-2-(4-fluorophenyl)-1H-indol-7-yl] Acetamide* (**4f**). A mixture of **3f** (0.15 g, 0.39 mmol) and TFAA (0.12 g, 0.58 mmol) in THF (10 mL) afforded **4f** as a solid (0.15 g, 81%), *R_f_* 0.34, mp. 222–225 °C; ν_max_ (ATR) 478, 514, 524, 578, 624, 733, 785, 816, 845, 920, 964, 1012, 1097, 1156, 1197, 1226, 1301, 1372, 1446, 1507, 1598, 1641, 1657, 3181, 3251 cm^−1^; ^1^H-NMR (DMSO-*d*_6_) 2.12 (3H, s, CH_3_), 7.14 (1H, d, *J* = 16.5 Hz, CH=CH), 7.20 (2H, t, *J* = 9.3 Hz, 3″,5″-H), 7.37 (1H, d, *J* = 16.5 Hz, CH=CH), 7.41 (2H, t, *J* = 8.7 Hz, 3′,5′-H), 7.66–7.73, (4H, m, 2′,6′-H and 2″,6″-H), 7.88 (1H, d, *J* = 1.2 Hz, 4-H), 8.01 (1H, d, *J* = 1.2 Hz, 6-H), 9.77 (1H, s, NH), 12.43 (1H, s, NH); ^13^C-NMR (DMSO-*d*_6_) 24.1, 107.1, 114.8, 115.6 (d, ^2^*J*_CF_ = 21.8 Hz), 116.0 (d, ^2^*J*_CF_ = 20.6 Hz), 116.2, 116.3, 117.0 (q, ^1^*J*_CF_ = 230.2 Hz), 125.0, 126.6, 128.0 (d, ^4^*J*_CF_ 3.5 Hz), 128.6, 128.8 (d, ^3^*J*_CF_ 8.0 Hz), 129.0, 129.6, 132.8 (d, ^3^*J J* 8.0 Hz), 133.3, 134.2 (d, ^4^*J*_CF_ = 3.5 Hz), 147.9, 162.0 (d, ^1^*J*_CF_ = 243.9 Hz), 163.5 (d, ^1^*J*_CF_ = 246.2 Hz), 175.8 (q, ^2^*J*_CF_ = 34.4 Hz); ^19^F-NMR (DMSO-*d_6_*) −114.6 (1F, ddd, *J* = 5.9, 9.0 and 14.9 Hz 4″-CF), −110.9 (1F, ddd, *J* = 5.9, 9.0 and 14.9 Hz, 4′-CF), −71.2 (3F, s, COCF_3_); HRMS (ES): MH^+^, found: 485.1285. C_26_H_18_N_2_F_5_O_2_^+^ requires: 485.1288.

*((E)-N-(5-(4-Fluorostyryl)-2-(4-methoxyphenyl)-3-(2,2,2-trifluoroacetyl)-1H-indol-7-yl) Acetamide* (**4g**). A mixture of **3g** (0.15 g, 0.38 mmol) and TFAA (0.12 g, 0.56 mmol) in THF (10 mL) afforded **4g** as a solid (0.14 g, 74%), *R_f_* 0.20, mp. 264–267 °C; ν_max_ (ATR) 466, 527, 613, 633, 730, 749, 749, 817, 817, 846, 893, 922, 964, 1007, 1030, 1046, 1118, 1133, 1177, 1200, 1212, 1229, 1251, 1292, 1369, 1391, 1442, 1506, 1560, 1606, 1653, 1668, 3179, 3240 cm^−1^; ^1^H-NMR (DMSO-*d_6_*) 2.12 (3H, s, CH_3_), 3.84 (3H, s, OCH_3_), 7.11 (2H, d, *J* = 8.7 Hz, 3′,5′-H), 7.14‒7.22 (3H, m, CH=CH and 3″,5″-H), 7.36 (1H, d, *J* = 16.5 Hz, CH=CH), 7.55 (2H, d, *J =* 8.7 Hz, 2′,6′-H), 7.70 (2H, d, *J* = 8.7 Hz, 2″,6″-H), 7.89 (1H, d, *J* = 1.2 Hz, 4-H), 7.99 (1H, d, *J* = 1.2 Hz, 6-H) 9.74 (1H, s, NH), 12.30 (1H, s, NH); ^13^C-NMR (DMSO-*d_6_*) 24.2, 55.8, 107.7, 114.1 (2 × C), 115.2, 116.0 (d, ^2^*J*_CF_ = 20.6 Hz), 116.7 (q, ^1^*J*_CF_ *J* = 289.7 Hz), 123.7, 124.9, 126.5, 128.7, 128.8 (d, ^3^*J*_CF_ = 8.0 Hz), 129.6, 129.7, 131.8, 133.1, 134.2 (d, ^4^*J*_CF_ = 2.3 Hz), 149.1, 161.1, 162.0 (d, ^1^*J*_CF_ = 242.8 Hz), 169.2, 176.1 (q, ^2^*J*_CF_ = 35.6 Hz); ^19^F-NMR (DMSO-*d_6_*) -114.6 (1F, ddd, *J* = 5.9, 9.0 and 14.9 Hz, 4″-CF), -71.0 (3F, s, COCF_3_); HRMS (ES): MH^+^, found: 497.1488. C_27_H_21_F_4_N_2_O_3_^+^ requires: 497.1488.

### 3.5. Typical Procedure for the One-pot Sequential Beckmann Rearrangement of ***2a**–**g*** and Subsequent Trifluoroacetylation

A mixture of **2** (1 equivalent) and TFA (1.2 equivalent) in acetonitrile (25 mL/mmol of **2**) was refluxed at 80 °C for 2 h. TFAA (2 equiv.) was added to the mixture and heating was continued for 5 h at this temperature. The solvent was evaporated under reduced pressure and the residue was quenched with an ice-cold water. The product was extracted with chloroform and the combined organic layers were dried over anhydrous MgSO_4._ The salt was filtered off and then the solvent was evaporated under reduced pressure on a rotary evaporator. The residue was purified by column chromatography on a silica gel to afford **4** as a solid. Products **4a**–**f** were prepared in this fashion. 

*N-(2,5-Bis(4-fluorophenyl)-3-(2,2,2-trifluoroacetyl)-1H-indol-7-yl) Acetamide* (**4a**). A mixture of **2a** (0.15 g, 0.41 mmol) and TFA (0.06 g, 0.50 mmol) in CH_3_CN (10 mL) followed by treatment with TFAA (0.17 g, 0.83 mmol) afforded **4a** as a solid (0.14 g, 74%).

*N-(5-(4-Fluorophenyl)-2-(4-methoxyphenyl)-3-(2,2,2-trifluoroacetyl)-1H-indol-7-yl) Acetamide* (**4b**). A mixture of **2b** (0.15 g, 0.40 mmol) and TFA (0.05 g, 0.48 mmol) in CH_3_CN (10 mL) followed by treatment with TFAA (0.17 g, 0.80 mmol) afforded **4b** as a solid (0.15 g, 80%).

*N-(3-(2,2,2-Trifluoroacetyl)-2-(4-fluorophenyl)-5-(4-methoxyphenyl)-1H-indol-7-yl) Acetamide* (**4c**). A mixture of **2c** (0.15 g, 0.40 mmol) and TFA (0.05 g, 0.48 mmol) in CH_3_CN (10 mL) followed by treatment with TFAA (0.17 g, 0.80 mmol) afforded **4c** as a solid (0.16 g, 82%).

*N-(3-(2,2,2-Trifluoroacetyl)-2,5-bis(4-methoxyphenyl)-1H-indol-7-yl) Acetamide* (**4d**). A mixture of **2d** (0.15 g, 0.39 mmol) and TFA (0.05 g, 0.47 mmol) in CH_3_CN (10 mL) followed by treatment with TFAA (0.16 g, 0.78 mmol) afforded **4d** as a solid (0.16 g, 84%).

*(E)-N-[5,[5-(4-Fluorostyryl)-3-(2,2,2-trifluoroacetyl)-2-phenyl-1H-indol-7-yl] Acetamide* (**4e**). A mixture of **2e** (0.15 g, 0.41 mmol) and TFA (0.6 g, 0.48 mmol) in CH_3_CN (10 mL) followed by treatment with TFAA (0.17 g, 0.81 mmol) afforded **4e** as a solid (0.14 g, 76%).

*(E)-N-[5,[5-(4-Fluorostyryl)-3-(2,2,2-trifluoroacetyl)-2-(4-fluorophenyl)-1H-indol-7-yl] Acetamide* (**4f**). A mixture of **2f** (0.15 g, 0.39 mmol) and TFA (0.05 g, 0.46 mmol) in CH_3_CN (10 mL) followed by treatment with TFAA (0.16 g, 0.77 mmol) afforded **4f** as a solid (0.15 g, 79%).

*((E)-N-(5-(4-Fluorostyryl)-2-(4-methoxyphenyl)-3-(2,2,2-trifluoroacetyl)-1H-indol-7-yl) Acetamide* (**4g**). A mixture of **2g** (0.15 g, 0.38 mmol) and TFA (0.05 g, 0.46 mmol) in CH_3_CN (10 mL) followed by treatment with TFAA (0.16 g, 0.75 mmol) afforded **4g** as a solid (0.13 g, 72%).

### 3.6. pLDH Asssay

Three-fold serial dilutions of the test compounds were incubated in triplicates with 3D7 strain *P. falciparum* parasites in a transparent 96-well flat bottom plate. DMSO and chloroquine were used as negative and positive controls, respectively. The plate was put in an airtight box, gassed and incubated with complete RPMI 1640 medium for 48 h. At the end of incubation, Malstat reagent was added to the 96-well plate followed by developing with NBT/PES (nitro blue tetrazolium + phenazine ethosulphate) reagent. Parasite growth was determined spectrophotometrically at 620 nm, by measuring the activity of the pLDH in control and drug-treated cultures using an Infinite F500 multiwell plate reader (Tecan Group Ltd., Zürich, Switzerland). The OD values from control wells devoid of drug were referred to as having 100% pLDH activity. The IC_50_ are expressed as the % parasite survival relative to the control, calculated from fitted sigmoidal dose response curves. The dose response curves were obtained by plotting percentage parasite survival against the logarithm of the concentration using the GraphPad Prism software package (GraphPad software Inc., La Jolla, CA, USA). IC_50_ values were calculated graphically by interpolation from these curves.

### 3.7. In Vitro Cytotoxicity Assay

Monkey kidney cells (Vero) used in this experiment were obtained from Cellonex (Johannesburg, South Africa). The cells were maintained in Dulbecco’s Modified Eagle’s (DMEM, HyClone, Thermo Scientific, Aalst, Belgium) supplemented with 0.4 mML-glutamine and sodium pyruvate and 10% foetal bovine serum (FBS, HyClone, Thermo Scientific). The cells of a sub-confluent culture were harvested using trypsin-EDTA (HyClone, Thermo Scientific) and centrifuged at 200× *g* for 5 min and re-suspended in growth medium to 5 × 10^4^ cells/mL. A total of 200 µL of the cell suspension was pipetted into each well of columns 2 to 11 of a 96 well culture plate. The same amount of the growth medium was added to wells of column 1 and 12 to maintain humidity and minimize the edge effect. The plates were incubated at 37 °C in a 5% CO_2_ incubator overnight until the cells were in the exponential phase of growth. After incubation, the DMEM was aspirated from the cells and replaced with 200 µL of different concentrations of the test samples (0.1–100 µM). Each dilution of the test sample was tested in quadruplicate in each experiment and the experiments were repeated three times. The plates were again incubated for 2 days at 37 °C in a 5% incubator. A negative control (untreated cells) and positive control (cells treated with different concentrations of doxorubicin hydrochloride, Sigma, Schnelldorf, Germany) were included. After incubation, 30 µL of 5 mg/mL MTT, (Sigma-Aldrich, GmBH, Schnelldorf, Germany) in phosphate buffered saline PBS was added to each well and the plates were incubated for a further 4 h at 37 °C. After incubation with MTT, the medium in each well was removed and the formazan crystals formed were dissolved by adding 50 µL of DMSO to each well of the plates. The plates were gently shaken until the crystals were dissolved. The amount of MTT reduction was measured immediately by detecting the absorbance using a microplate reader at a wavelength of 570 nm (BioTek Synergy, Analytical and Diagnostic Products, Johannesburg, South Africa). The wells in column 1 and 12, containing medium and MTT but no cells was used to blank the microplate reader. The percentage of cell viability was calculated using the formula below:

$$\%\text{Cell}\;\text{viability}\;=\;\frac{\text{Mean}\;\text{Absorbance}\;\;\text{of}\;\text{sample}\;\times \;100}{\text{Mean}\;\text{Absorbance}\;\text{of}\;\text{control}}$$

The LC_50_ values (lethal concentration at which 50% of the cells are killed) were calculated as the concentration of the test sample that resulted in 50% reduction of absorbance compared to untreated cells. The intensity of the MTT formazan produced by living metabolically active cells is directly proportional to the number of live cells present.

### 3.8. Methodology-Docking Simulation

#### 3.8.1. Protein Structure

The starting structure of parasite lactate dehydrogenase (pLDH) was obtained from PDB (PDB id: 1T24) with 1.7 Å resolution. All heteroatoms and water molecules were removed pLDH. Polar hydrogen atoms, Kollman-Amber united atom partial charges and solvation parameters were added by utilizing AutoDockTools [29].

#### 3.8.2. Ligand Structure

The initial structure of positive control 4-hydroxy-1,2,5-oxazole-3-carboxylic acid (OXD) was obtained from the ligand of pLDH (PDB id: 1T24) while the coordinates for test compound **3a**, **3f**, **4a** and **4g** were generated using Hyperchem 7.0 (Hypercube Inc., Gainesville, FL, USA). All ligands were retained with polar hydrogen atoms. Gasteiger charges and torsional angles were added by utilizing AutoDockTools [29].

#### 3.8.3. Molecular Docking Simulation

Grid maps of 40 × 40 × 40 points with 0.375 Å spacing were centered at OXD binding site in the crystal structure (PDB id: 1T24) for positive control docking and the docking of lactate (the substrate of pLDH). While for compound **3**(**a**,**f**) and **4**(**a**,**g**), the grid maps of 50 × 50 × 50 points with 0.375 Å spacing were centered at NAD+ of the crystal structure (PDB id: 1T24). A total of 200 docking runs by AutoDock4.2.6 [30] were performed by employing Lamarckian genetic algorithm with 2,500,000 energy evaluations per run and maximum number of 27,000 generation. The number of individuals in population was 150 and the crossover rate was 0.8. The results were processed by conformational cluster analysis with 1.0 Å cut-off for positive control and 2.0 Å cut-off for lactate, **3**(**a**,**f**) and **4**(**a**,**g**). The ligand conformation with lowest free energy of binding in the most populated cluster was selected for comparison.

## 4. Conclusions

The oximes derived from the 1-(2,5-diarylindol-7-yl)ethanones undergo sequential or direct one-pot successive TFA-mediated Beckmann rearrangement followed by trifluoroacetylation of the intermediate *N*-(2,5-diarylindol-7-yl)acetamides with TFAA to afford the C-3 trifluoroacetylated derivatives, exclusively. The preliminary in vitro antiplasmodial activity results against the chloroquine-sensitive strain of malaria parasite for both *N*-(2,5-diarylindol-7-yl)acetamides and their 3-trifluoroacetyl–substituted derivatives reveal that the entire 7-aminoindole framework is required for biological activity. Structure-activity relationship and molecular docking indicate that the optimal aryl substituent at the 5-position of the indole derivatives **3** and **4** is the 4-fluorophenyl or 4-fluorostyryl group. Molecular docking of the most active compounds **3a**, **3f**, **4a** and **4g** against the parasite lactate dehydrogenase (pLDH) reveal that the indole derivatives prepared in this investigation presumably act through different mechanism of action to that of chloroquine. They will probably not compete with lactate for its binding site, but might block the entrance of lactate to cause the observed inhibition effects. Future studies should involve an enzyme inhibition assay against lactate dehydrogenase activity to measure parasite viability. Despite the fact that the IC_50_ values of these compounds (1400–14,000 nM) fall much short of the nanomolar activity of chloroquine (10 nM), these preliminary antiplasmodial activity results suggest that the indole derivatives are worthy of further studies to develop new drugs with novel mechanisms of action and reduced toxicity. 

## Figures and Tables

**Table:** 

Compound	Ar^1^	Ar^2^	%Yield of 2	%Yield of 3
**a**	4-FC_6_H_4_-	4-FC_6_H_4_-	94	80
**b**	4-MeOC_6_H_4_-	4-FC_6_H_4_-	87	75
**c**	4-FC_6_H_4_-	4-MeOC_6_H_4_-	88	73
**d**	4-MeOC_6_H_4_-	4-MeOC_6_H_4_-	88	70
**e**	C_6_H_5_-	4-FC_6_H_4_CH=CH-	76	78
**f**	4-FC_6_H_4_-	4-FC_6_H_4_CH=CH-	75	77
**g**	4-MeOC_6_H_4_-	4-FC_6_H_4_CH=CH-	76	69

**Table:** 

Compound	Ar ^1^	Ar ^2^	%Yield from 3	%Yield from 2
**4a**	4-FC_6_H_4_-	4-FC_6_H_4_-	79	74
**4b**	4-MeOC_6_H_4_-	4-FC_6_H_4_-	86	80
**4c**	4-FC_6_H_4_-	4-MeOC_6_H_4_-	89	82
**4d**	4-MeOC_6_H_4_-	4-MeOC_6_H_4_-	90	84
**4e**	C_6_H_5_-	4-FC_6_H_4_CH=CH-	80	76
**4f**	4-FC_6_H_4_-	4-FC_6_H_4_CH=CH-	81	79
**4g**	4-MeOC_6_H_4_-	4-FC_6_H_4_CH=CH-	74	72

**Table 1:**
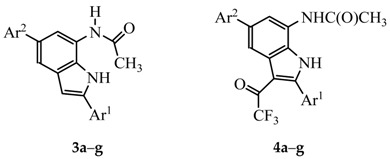
IC_50_ values of **3a**–**g** and **4a**–**g** and chloroquine against 3D7 strain of *P. falciparum.*

Compound	Ar^1^	Ar^2^	IC_50_ (μM)	SI
**3a**	4-FC_6_H_4_-	4-FC_6_H_4_-	1.43 ± 0.21	0.13
**3b**	4-MeOC_6_H_4_-	4-FC_6_H_4_-	6.34 ± 2.26	0.58
**3c**	4-FC_6_H_4_-	4-MeOC_6_H_4_-	6.33 ± 2.25	0.19
**3d**	4-MeOC_6_H_4_-	4-MeOC_6_H_4_-	11.01 ± 2.19	ND ^a^
**3e**	C_6_H_5_-	4-FC_6_H_4_CH=CH-	5.19 ± 1.58	0.16
**3f**	4-FC_6_H_4_-	4-FC_6_H_4_CH=CH-	2.14 ± 0.52	0.20
**3g**	4-MeOC_6_H_4_-	4-FC_6_H_4_CH=CH-	14.03 ± 2.57	0.42
**4a**	4-FC_6_H_4_-	4-FC_6_H_4_-	2.52 ± 0.91	0.23
**4b**	4-MeOC_6_H_4_-	4-FC_6_H_4_-	5.21 ± 1.56	0.48
**4c**	4-FC_6_H_4_-	4-MeOC_6_H_4_-	6.15 ± 2.53	0.56
**4d**	4-MeOC_6_H_4_-	4-MeOC_6_H_4_-	9.31 ± 0.97	0.28
**4e**	C_6_H_5_-	4-FC_6_H_4_CH=CH-	7.25 ± 1.34	0.66
**4f**	4-FC_6_H_4_-	4-FC_6_H_4_CH=CH-	5.15 ± 1.63	0.47
**4g**	4-MeOC_6_H_4_-	4-FC_6_H_4_CH=CH-	3.39 ± 0.86	0.31
Chloroquine			0.01 ± 2.59	

**^a^** Not determined.

**Table 2:** Cytotoxic effects of **3a**, **3f**, **4a** and **4g** against Monkey kidney Vero cell line.

Compound	LC_50_ (µM)
**3a**	13.78 ± 1.29 0.07
**3f**	>100
**4a**	32.69 ± 3.63
**4g**	>100
Doxorubicin hydrochloride	1.53 ± 0.22

**Table 3:** Estimated binding free energy and hydrogen bond of ligands with parasite lactase dehydrogenase (pLDH) calculated from docking simulation.

Ligand	Hb Distance (Å)	Interacting Atom	FEB (kcal/mol)	RMSD (Å)
OXQ (docked)	1.8	ASN140 2H_D2_–OXQ O_7_	−7.11	0.4
	1.8	ARG109 H_E_–OXQ O_7_		
	1.8	ARG171 2H_H1_–OXQ O_9_		
	1.9	ARG109 2H_H2_–OXQ O_8_		
	2.0	HIS195 H_E2_–OXQ O_7_		
	2.2	ARG171 2H_H2_–OXQ O_8_		
	2.3	NAD O_2D_–OXQ H_5_		
	2.5	HIS195 H_E2_–OXQ O_8_		
	2.7	ARG171 _2_H_H2_–OXQ O_9_		
Lactate (LAC)	1.8	ARG171 _2_H_H1_–LAC O_2_	−5.28	-
	1.8	ARG171 _2_H_H2_–LAC O_1_		
	1.8	HIS195 H_E2_–LAC O_3_		
	1.9	ARG109 H_E_–LAC O_3_		
	2.0	ARG109 _2_H_H2_–LAC O_1_		
	2.5	ASN140 _2_H_D2_–LAC O_3_		
	2.7	HIS195 H_E2_–LAC O_1_		
**3a**	2.1	NAD O_1A_–3a H_N1_	−5.95	-
	2.3	NAD O_1A_–3a H_N2_		
**3f**	2.2	LYS102 H_N_–3f O_1_	−6.46	-
	2.7	THR101 H_N_–3f O_1_		
**4a**	2.0	GLY99 O–4a H_N2_	−6.49	-
**4g**	2.2	LYS102 H_Z3_–4g O_2_	−6.40	-

FEB: Free energy of binding.

## Supplementary Materials

The following materials are available online. The % cell viability and IC_50_ values as well graphs for compounds **3a**–**g** and **4a**–**g** as well as X-ray data.
