# Supplementary material for: Synthesis and Evaluation of N-(3-Trifluoroacetyl-indol-7-yl) Acetamides for Potential In Vitro Antiplasmodial Properties
Source: Molecules. 2017 Jul 2;22(7):1099. doi: 10.3390/molecules22071099 (PMC6152062; doi:10.3390/molecules22071099)
Supplement: Supplementary file 1 [file molecules-22-01099-s001.pdf]

molecules

Supplementary information for,

Synthesis and Evaluation of the *N*-(3-trifluoroacetylindol-7-yl)acetamides for potential *in vitro* antiplasmodial properties

M.J. Mphahlele, M.M. Mmonwa and Y.S. Choong

**Supplementary 1:**	**Copies of ^{1}H- and ^{13}C NMR spectra of compounds 2–4 and ^{19}F NMR spectra of compounds 4**
**Supplementary 2:**	**% cell viability and LC_{50} values of chloroquine and compounds 3 and 4**
**Supplementary 3:**	**% cell viability of Vero cells exposed Doxorubicin chloride, 3a, 3f, 4a and 4g**
**Supplementary 4:**	**Crystal data and structure refinement, bond lengths and torsion angles of 4g**

Supplementary 1: ^{1}H- and ^{13}C-NMR Spectra of Compounds 2–4 and ^{19}F NMR spectra of compounds 4

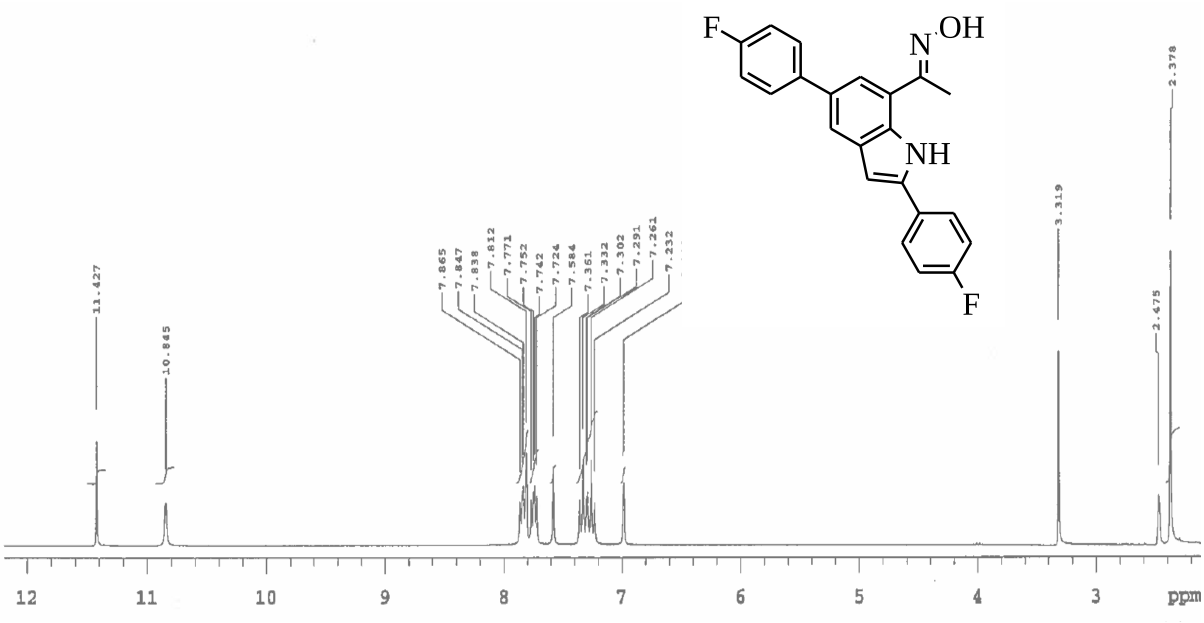

Figure S1. ^{1}H NMR Spectrum of Compound 2a DMSO-*d*$_6$ at 300 MHz

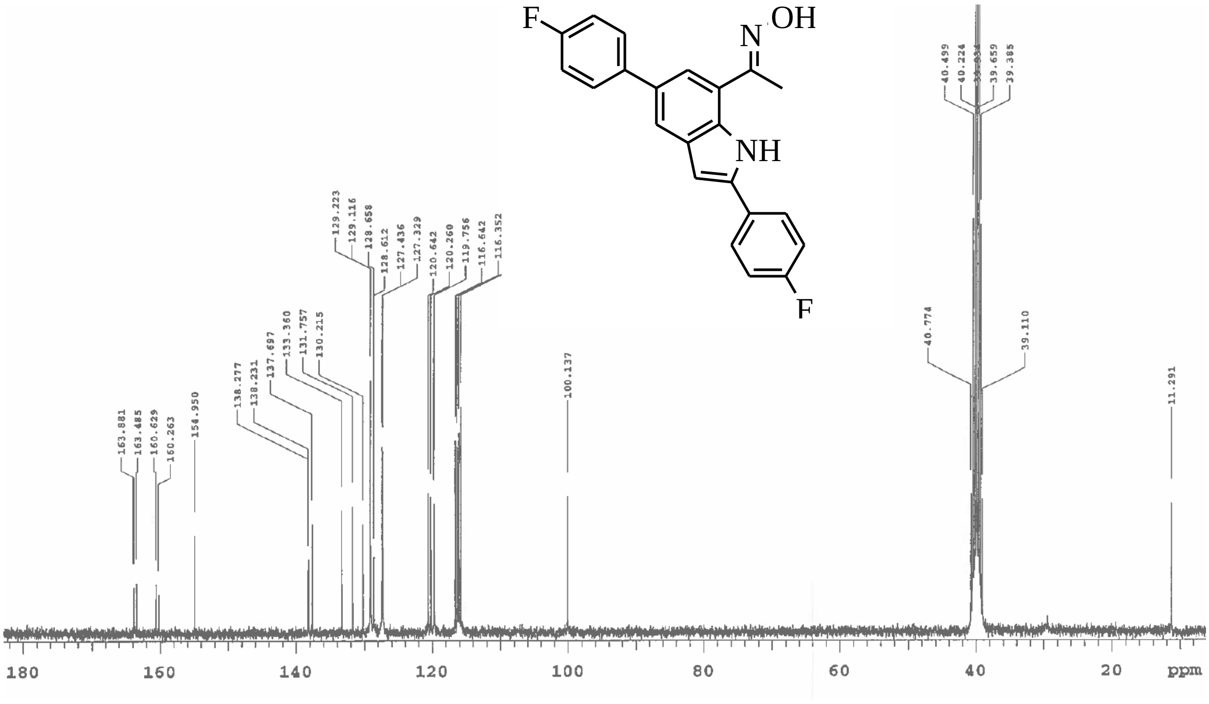

Figure S2. ^{13}C NMR Spectrum of Compound 2a in DMSO-*d*$_6$ at 75 MHz

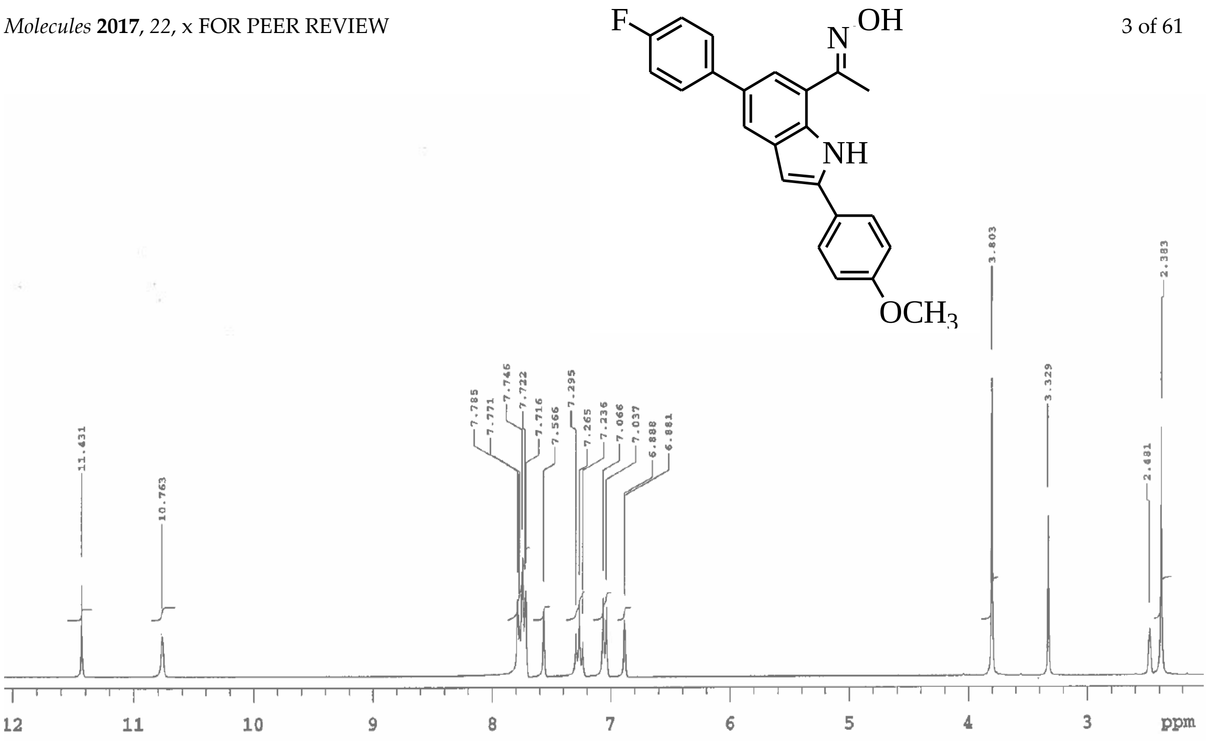

Figure S3. ^{1}H NMR Spectrum of Compound 2b in DMSO-*d*6 at 300 MHz

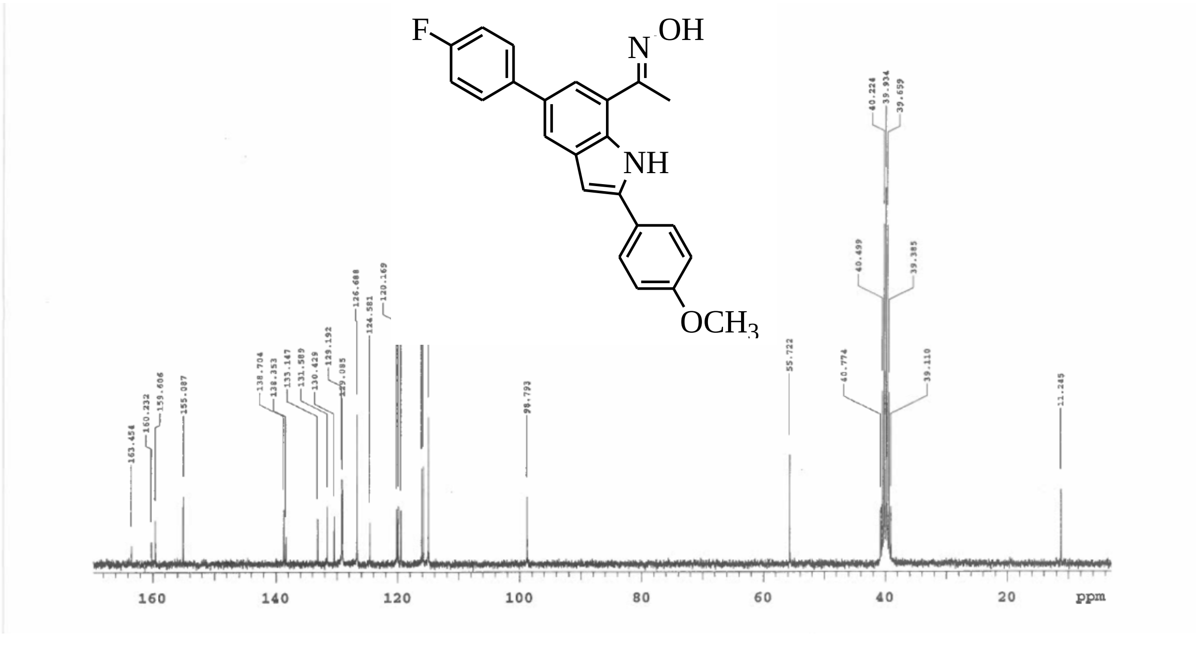

Figure S4. ^{13}C NMR Spectrum of Compound 2b in DMSO-*d*6 at 75 MHz

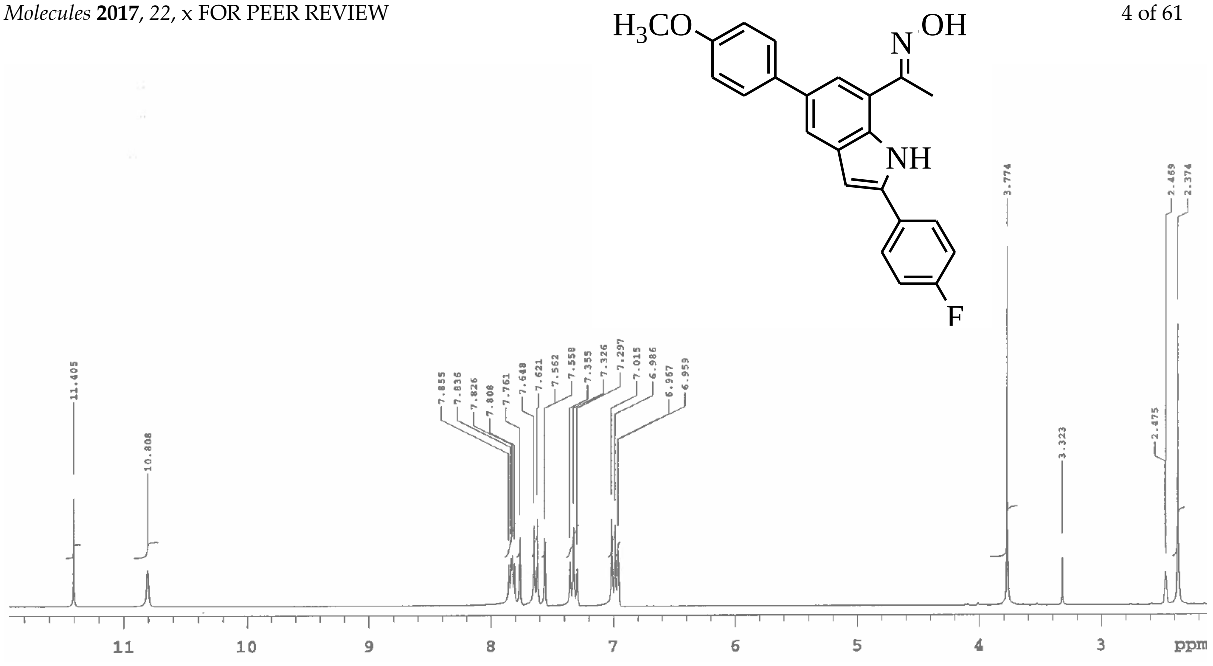

Figure S5. ¹H NMR Spectrum of Compound 2c in DMSO-*d*₆ at 300 MHz

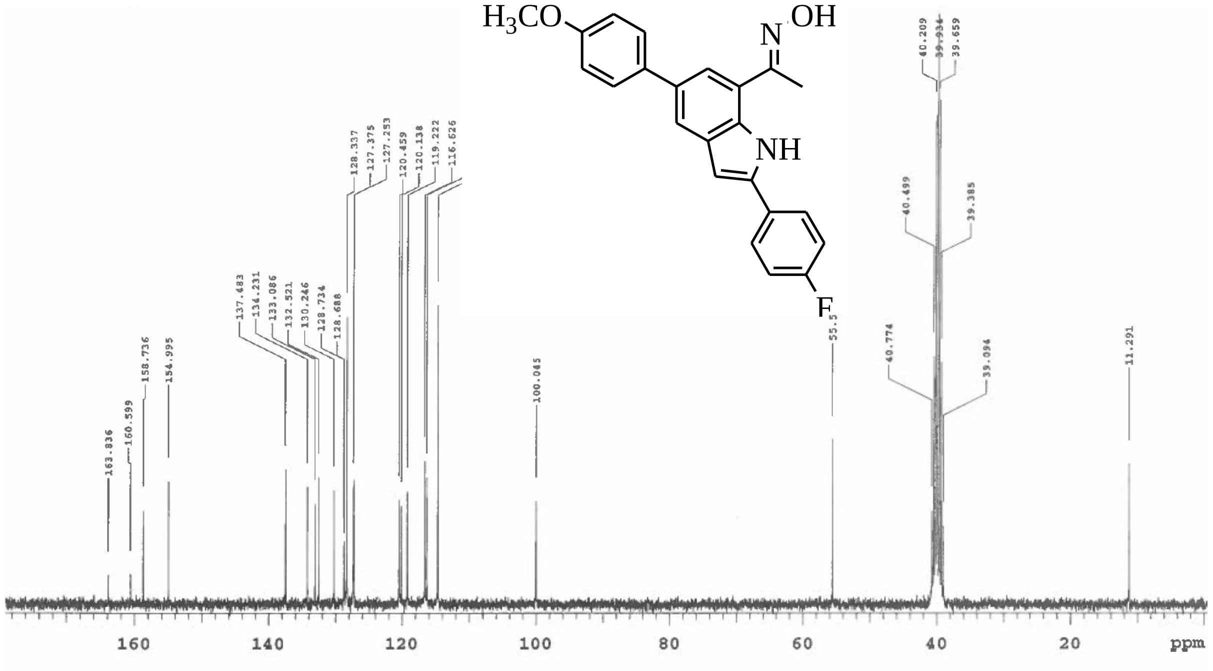

Figure S6. ¹³C NMR Spectrum of Compound 2c in DMSO-*d*₆ at 75 MHz

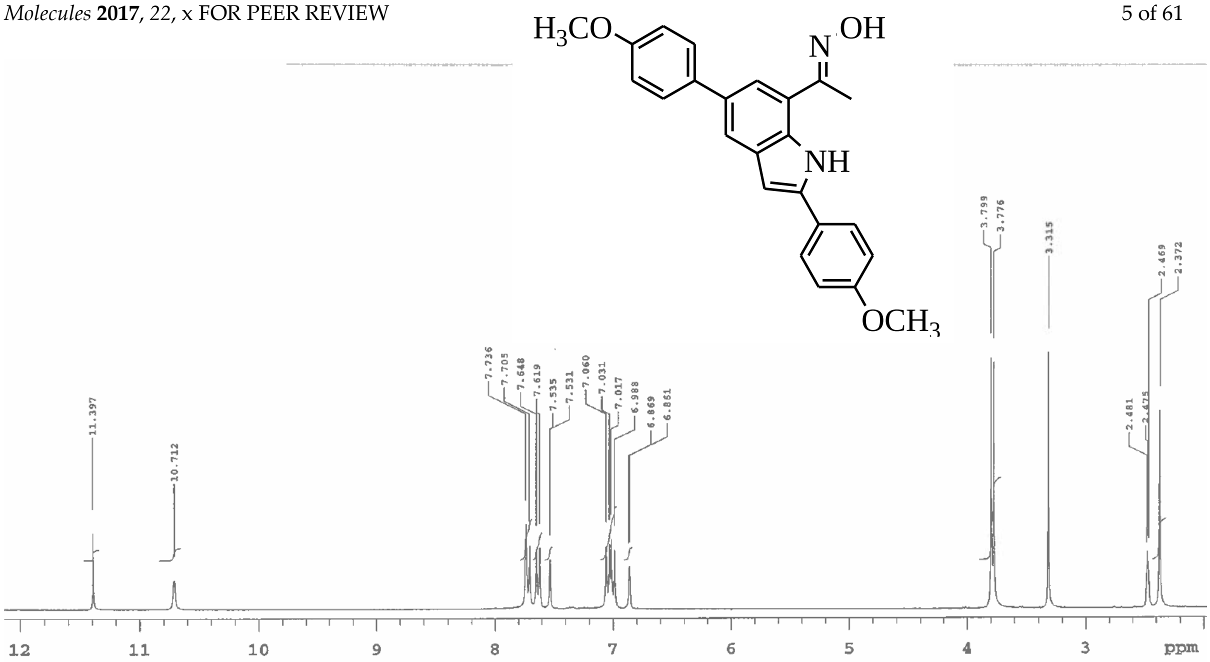

Figure S7. ¹H NMR Spectrum of Compound 2d in DMSO-*d*₆ at 300 MHz

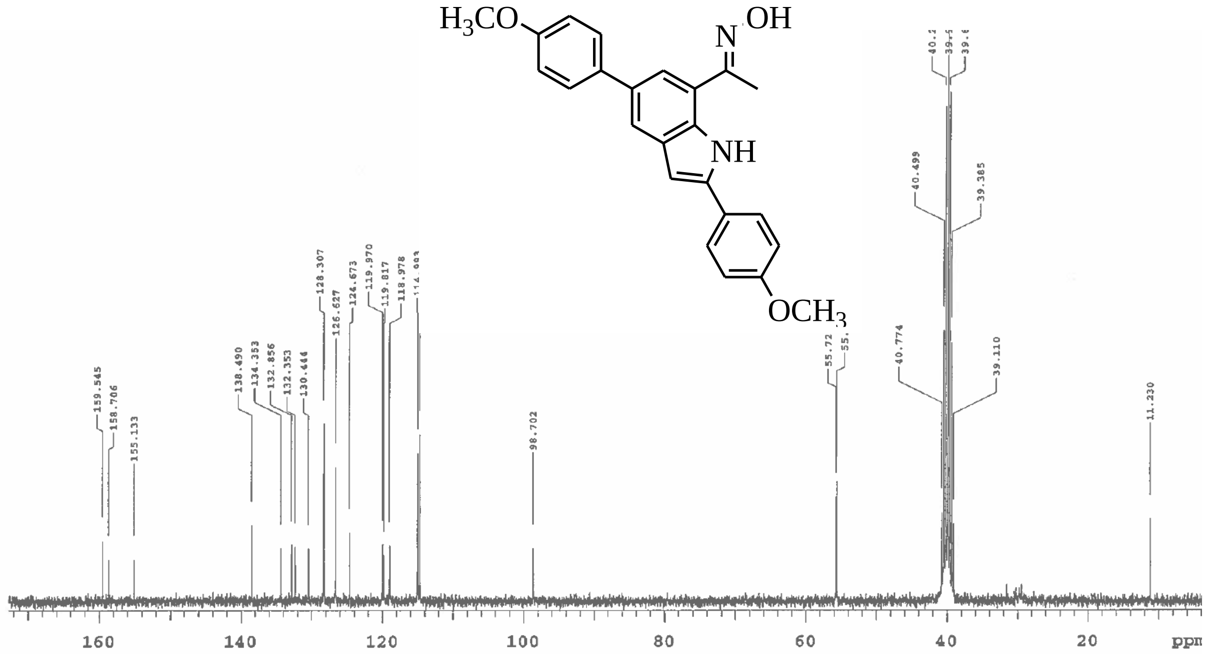

Figure S8. ¹³C NMR Spectrum of Compound 2d in DMSO-*d*₆ at 75 MHz

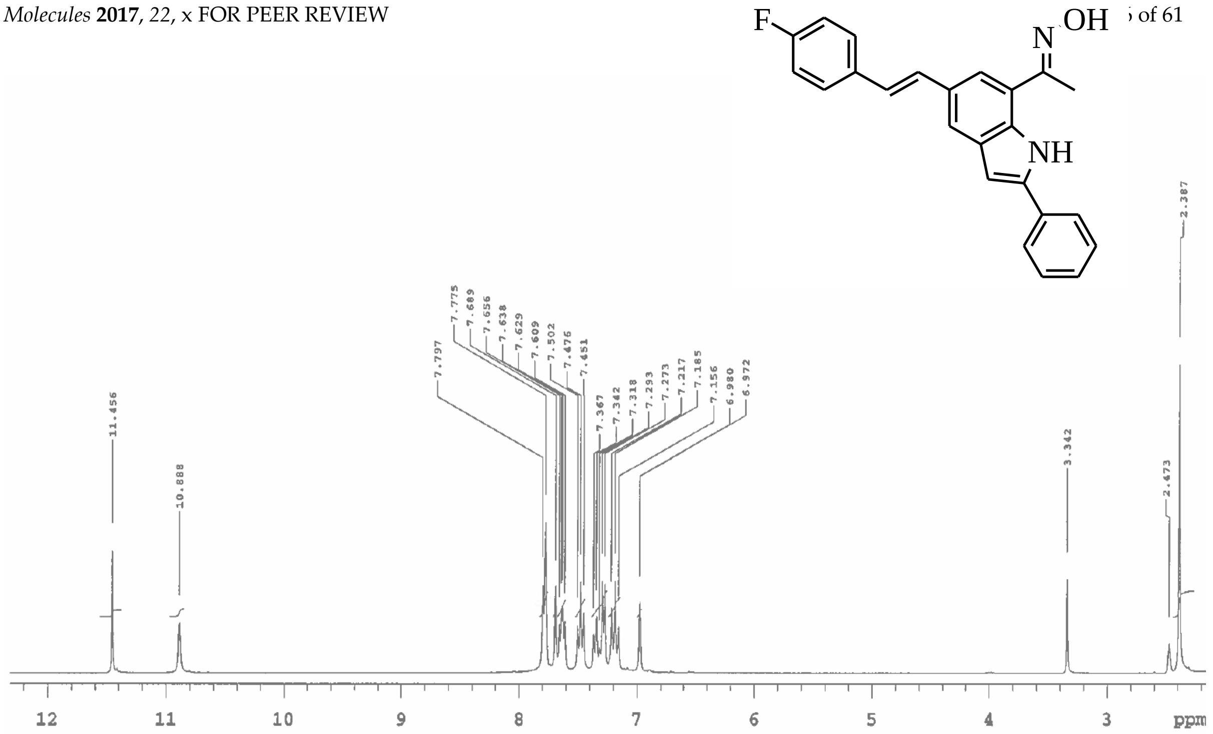

Figure S9. **^{1}H NMR Spectrum of Compound 2e in DMSO-*d*6 at 300 MHz**

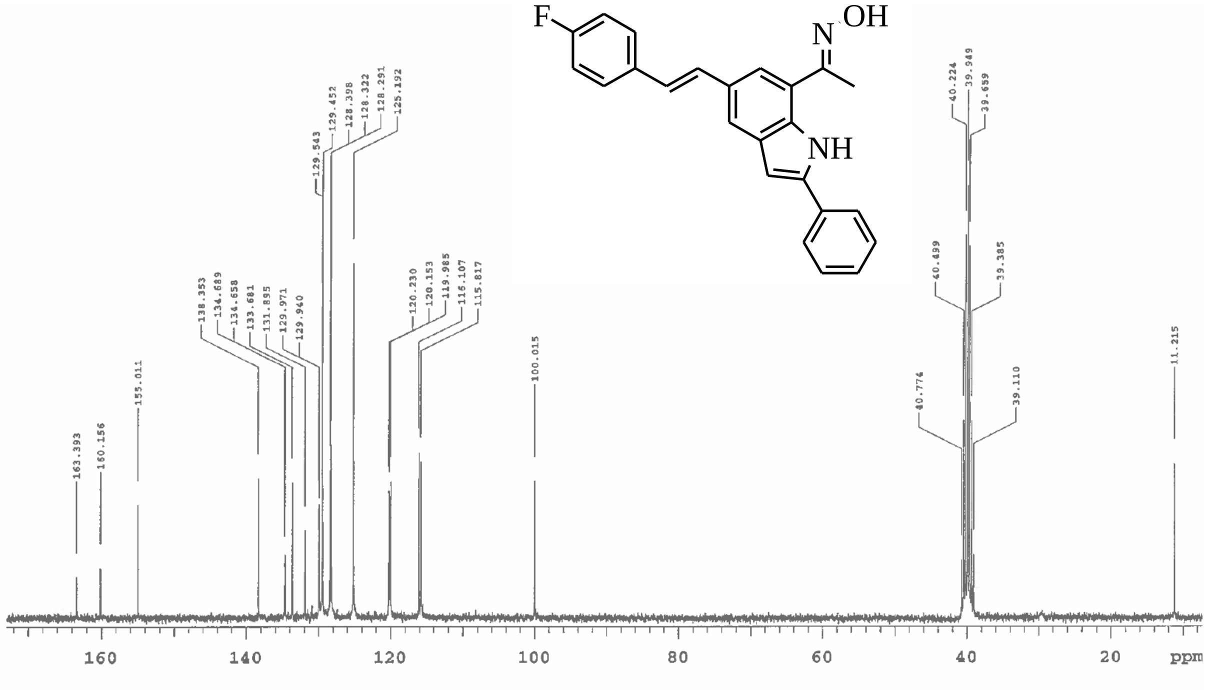

Figure S10. **^{13}C NMR Spectrum of Compound 2e in DMSO-*d*6 at 75 MHz**

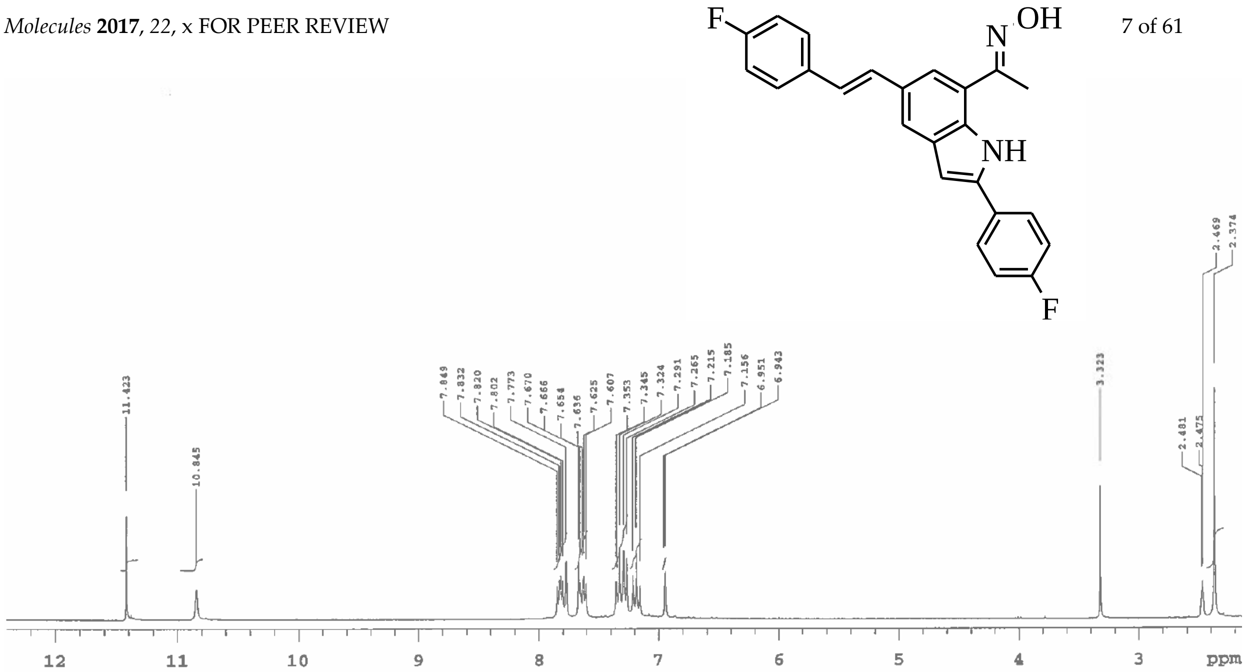

Figure S11. **^{1}H NMR Spectrum of Compound 2f in DMSO-*d*6 at 300 MHz**

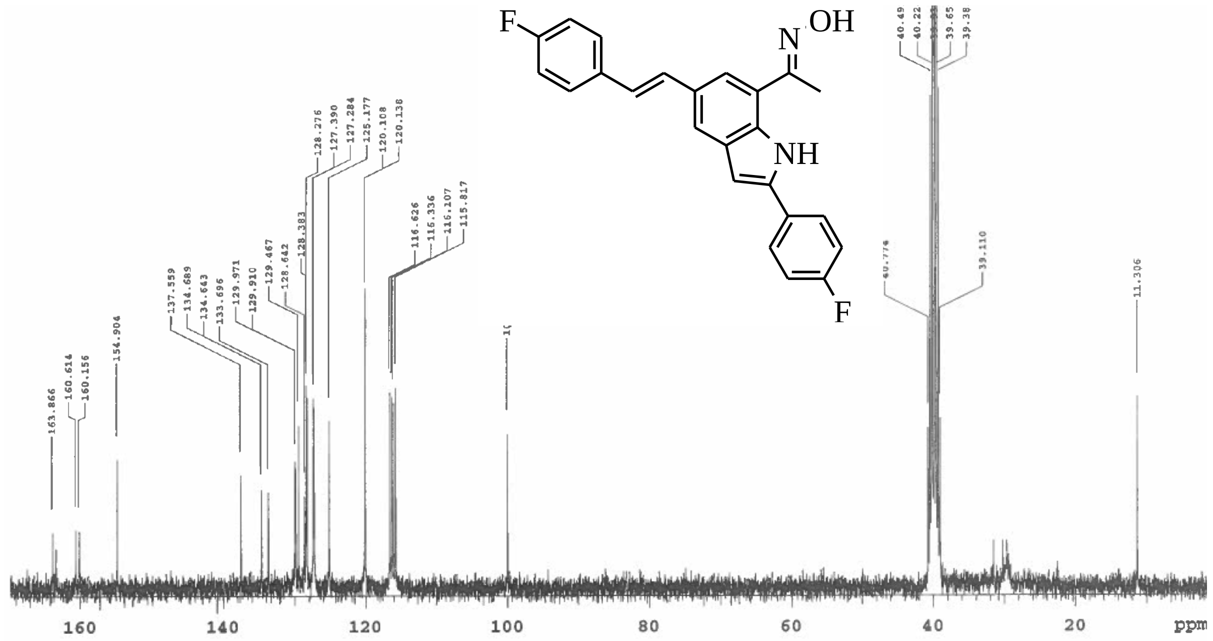

Figure S12. **^{13}C NMR Spectrum of Compound 2f in DMSO-*d*6 at 75 MHz**

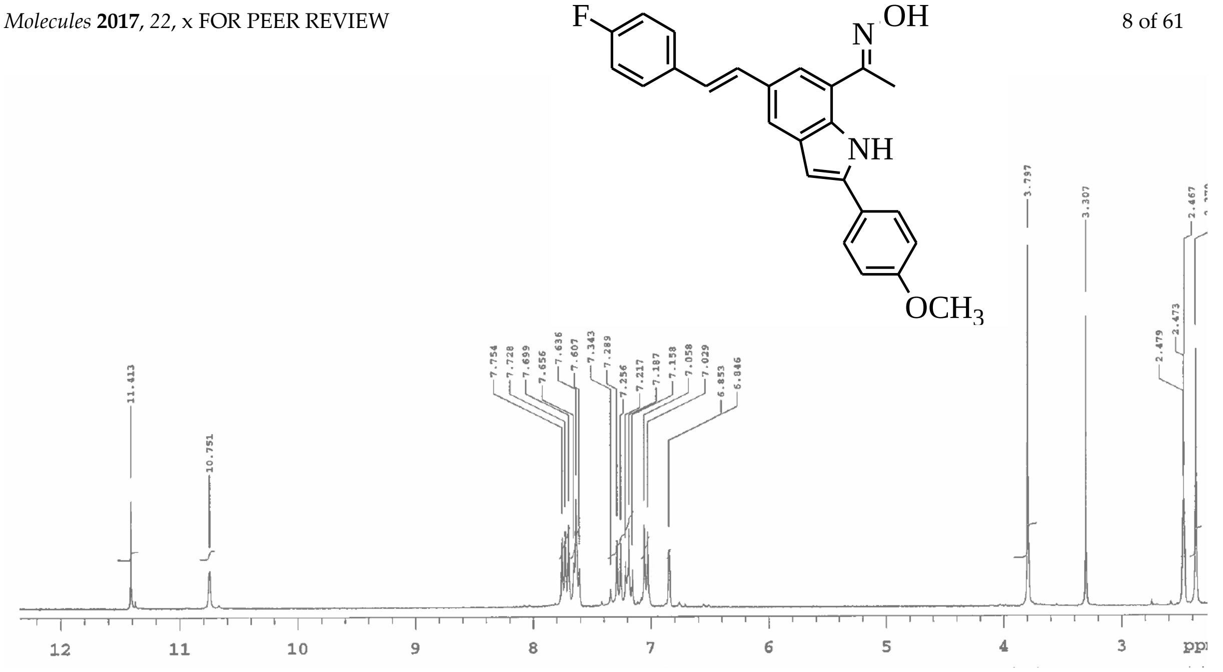

Figure S13. ^{1}H NMR Spectrum of Compound 2g in DMSO-d_6 at 300 MHz

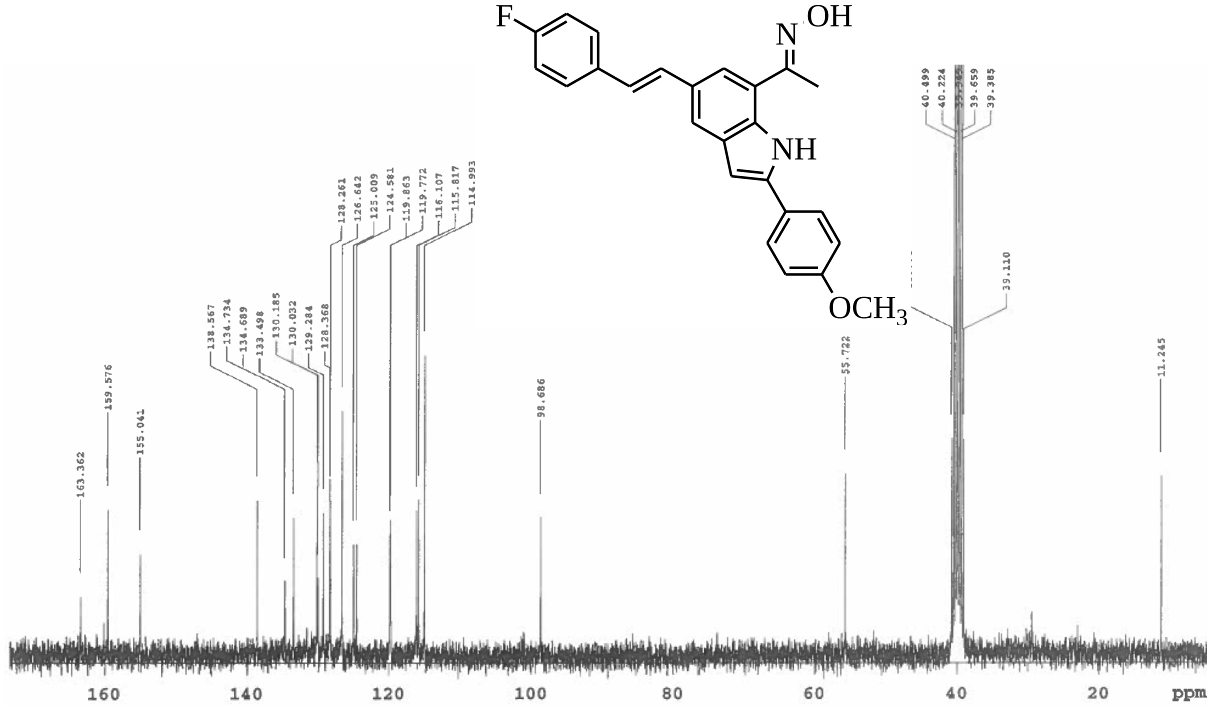

Figure S14. ^{13}C NMR Spectrum of Compound 2g in DMSO-d_6 at 75 MHz

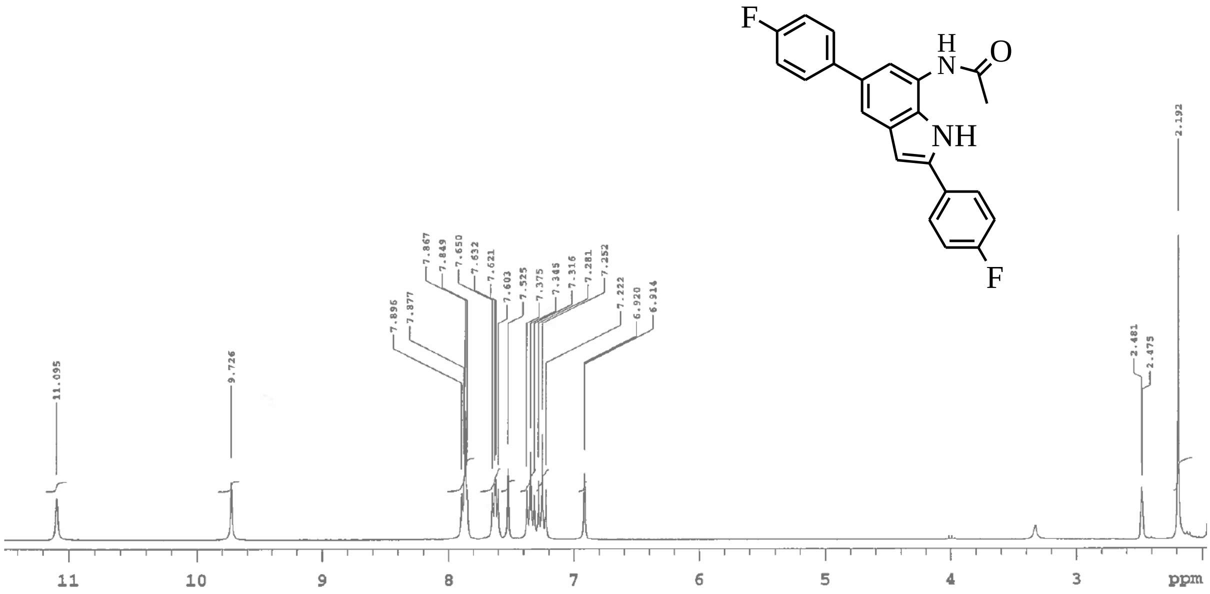

Figure S15. ¹H NMR Spectrum of Compound 3a in DMSO-*d₆* at 300 MHz

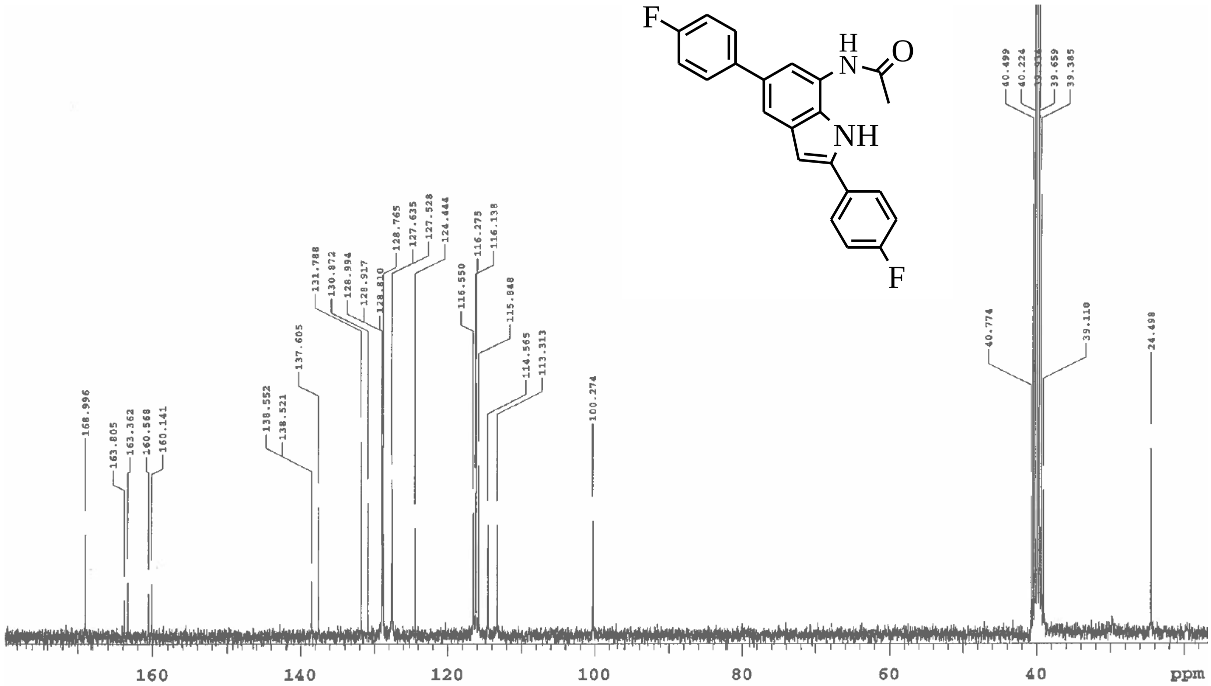

Figure S16. ¹³C NMR Spectrum of Compound 3a in DMSO-*d₆* at 75 MHz

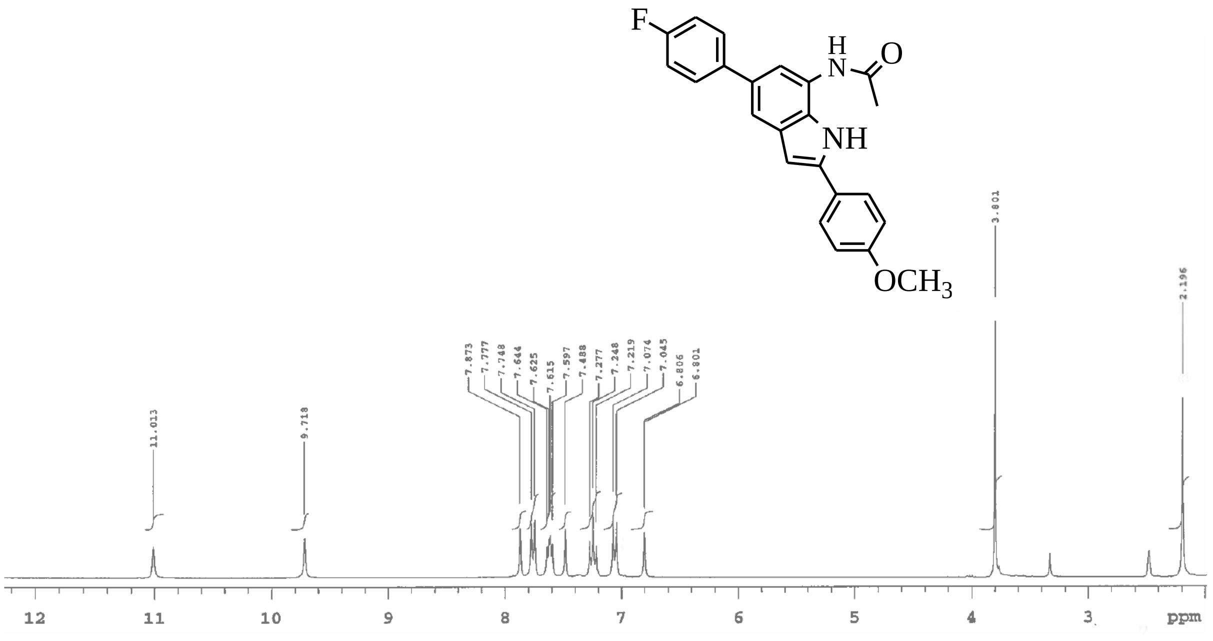

Figure S17. ^{1}H NMR Spectrum of Compound 3b in DMSO-*d₆* at 300 MHz

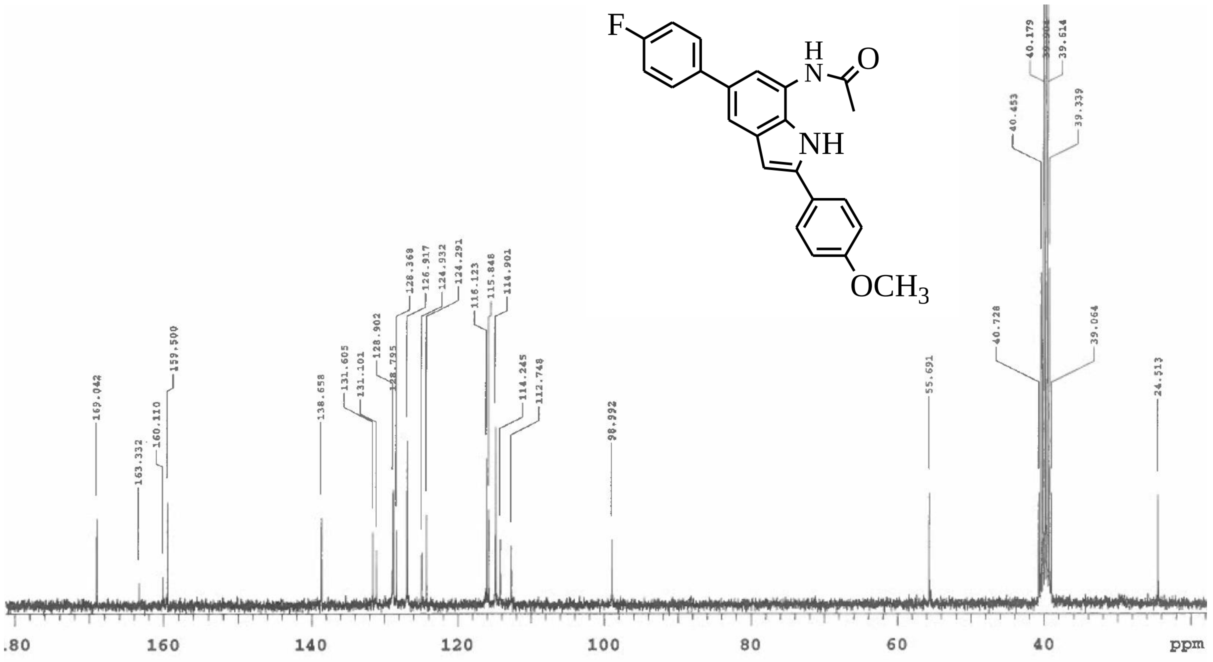

Figure S18. ^{13}C NMR Spectrum of Compound 3b in DMSO-*d₆* at 75 MHz

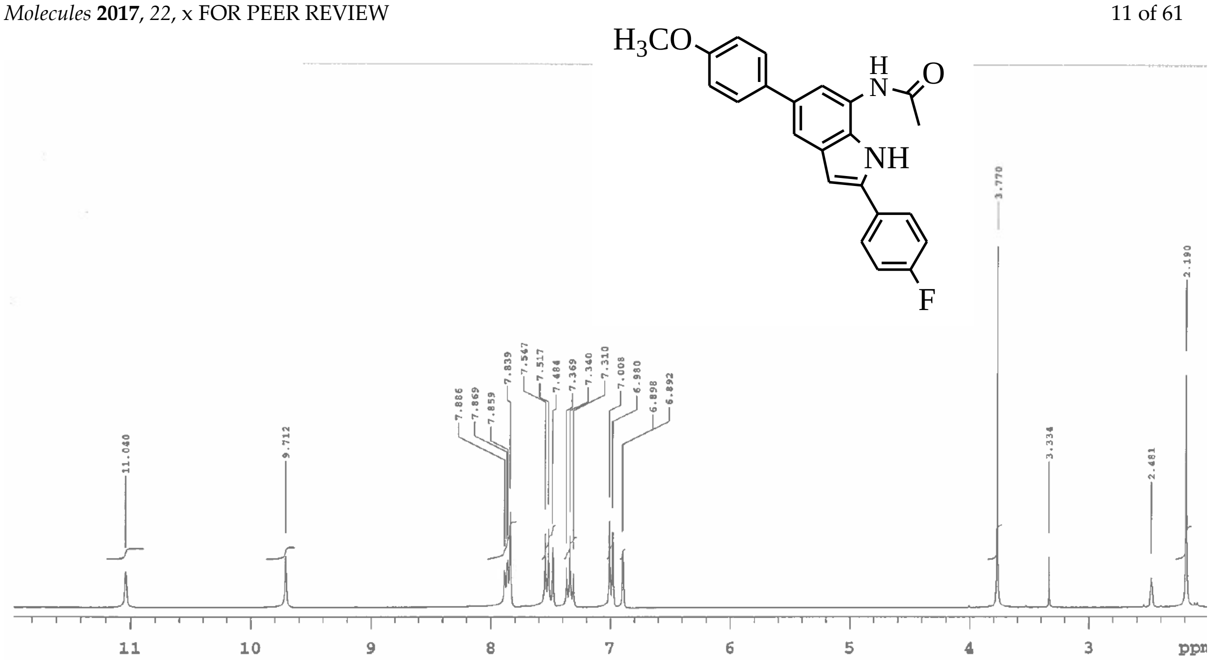

Figure S19. ^{1}H NMR Spectrum of Compound 3c in DMSO-*d*6 at 300 MHz

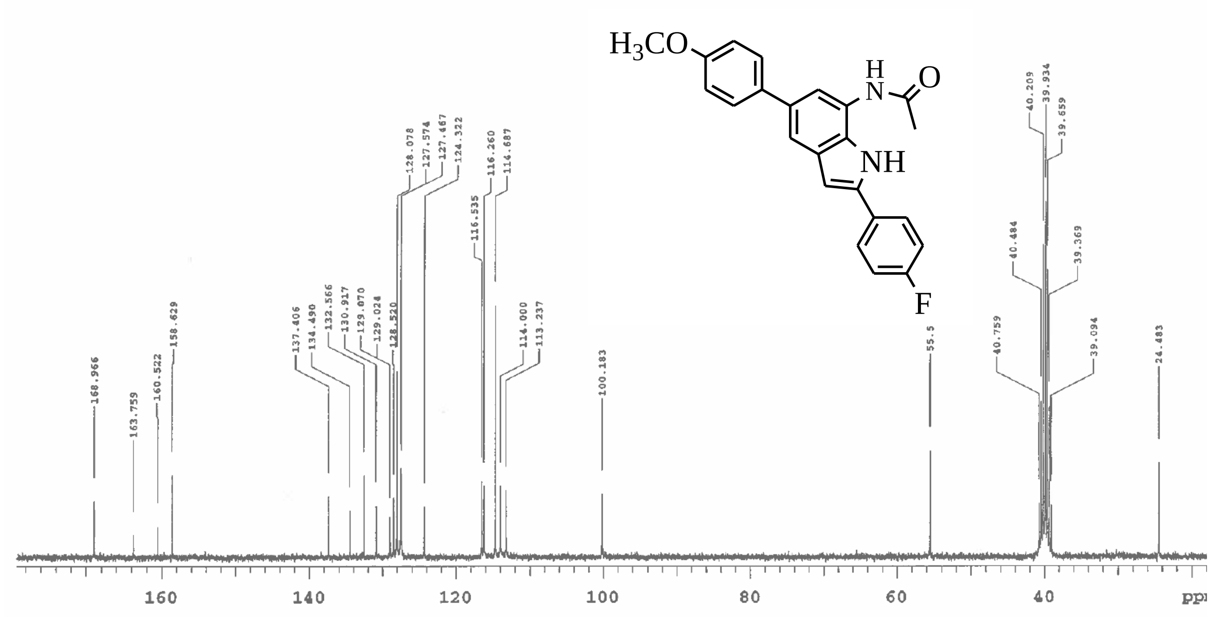

Figure S20. ^{13}C NMR Spectrum of Compound 3c in DMSO-*d*6 at 75 MHz

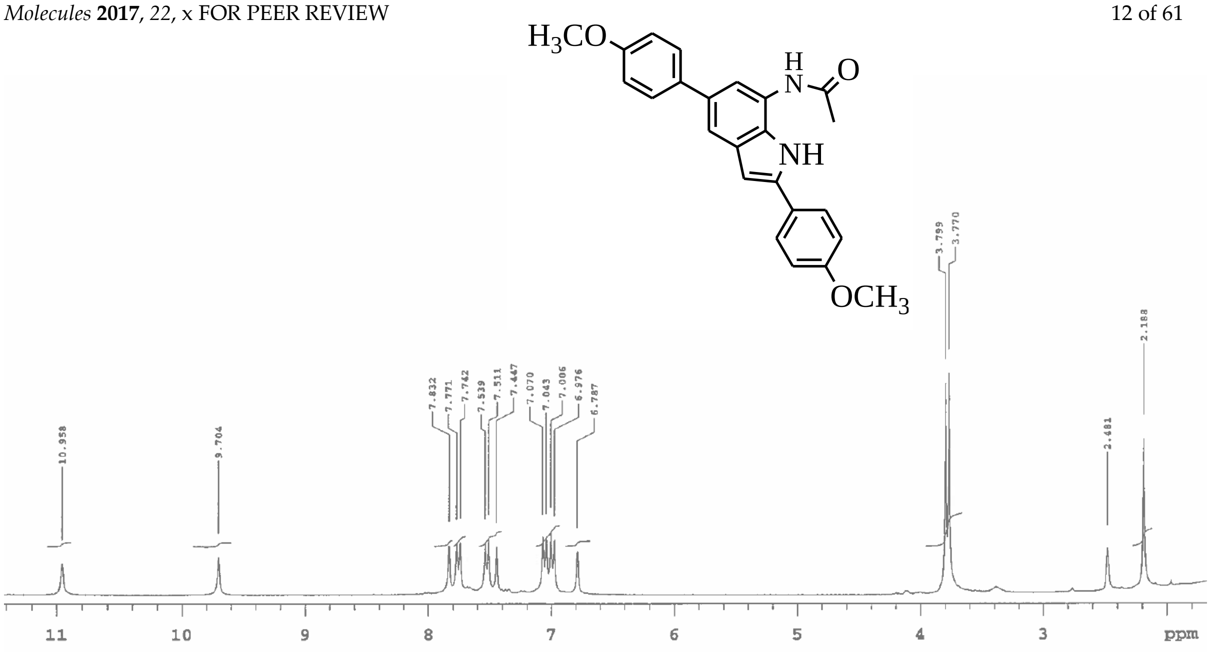

Figure S21. ^{1}H NMR Spectrum of Compound 3d in DMSO-*d*6 at 300 MHz

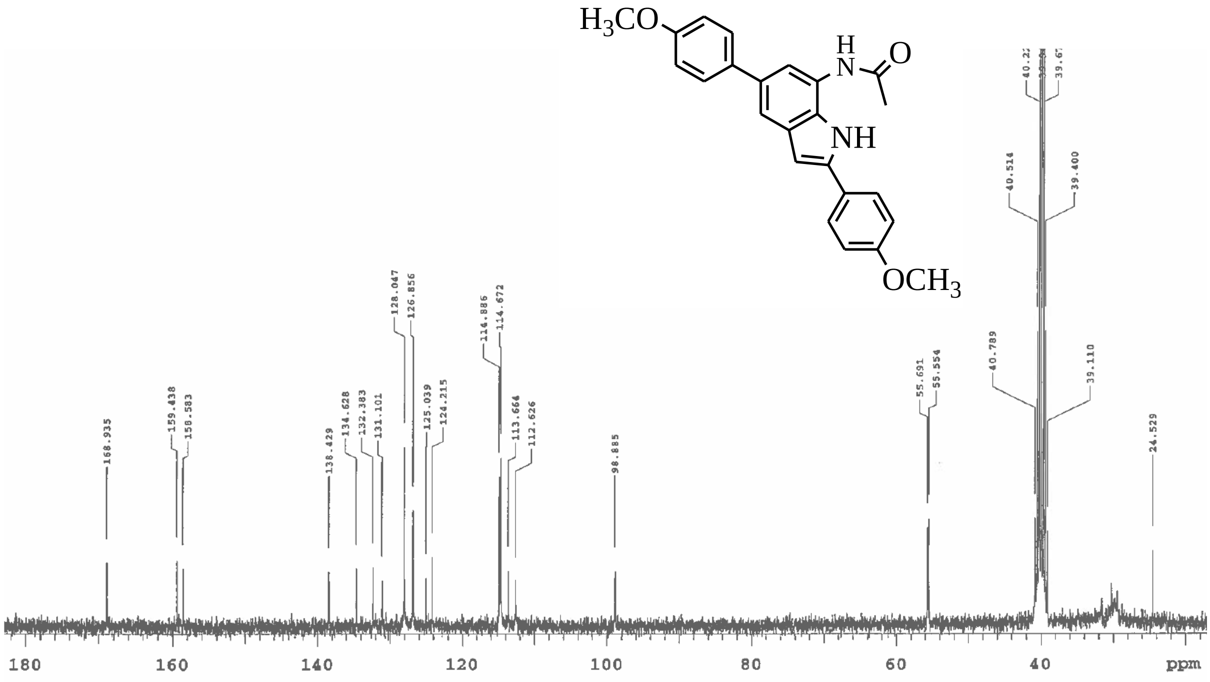

Figure S22. ^{13}C NMR Spectrum of Compound 3d in DMSO-*d*6 at 75 MHz

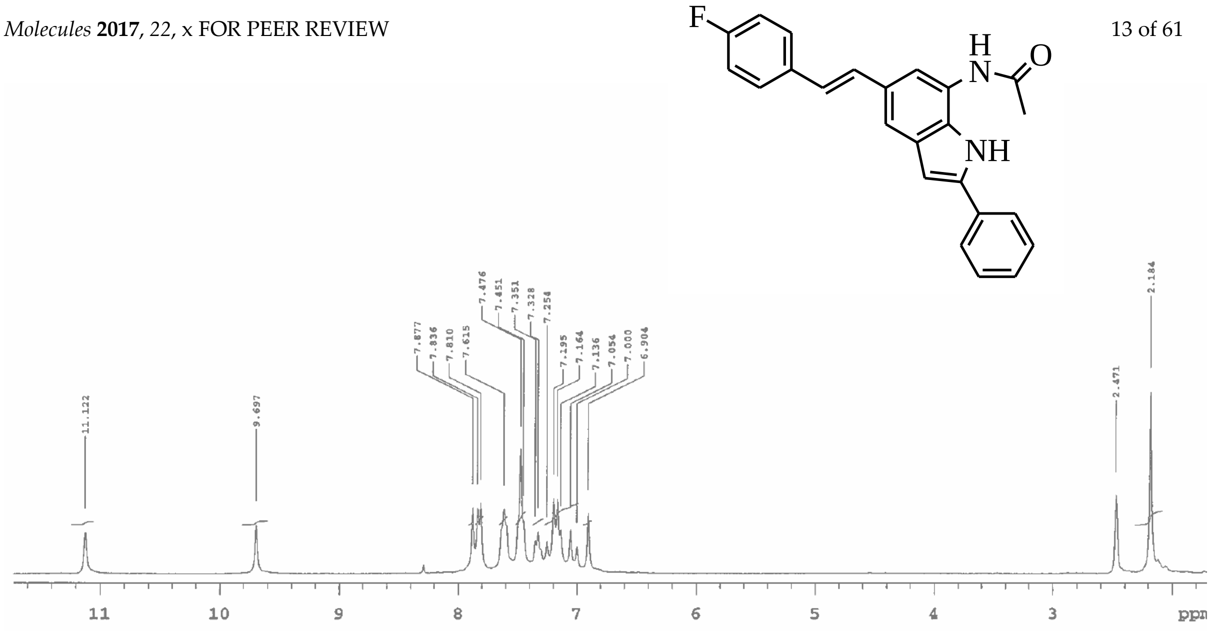

Figure S23. ^{1}H NMR Spectrum of Compound 3e in DMSO-*d*6 at 300 MHz

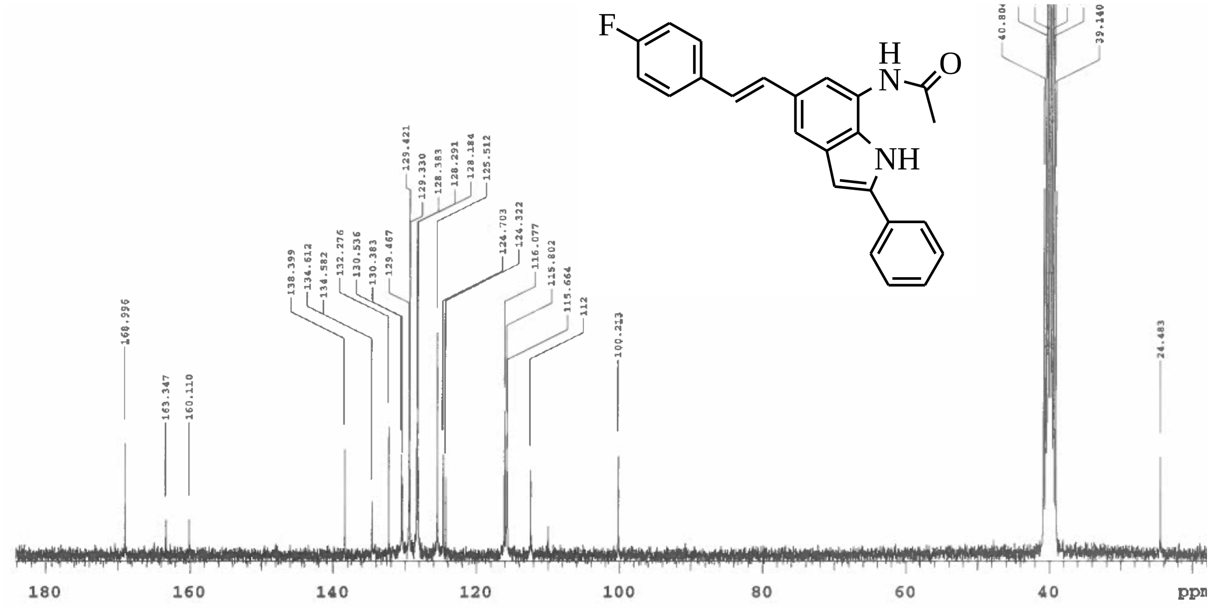

Figure S24. ^{13}C NMR Spectrum of Compound 3e in DMSO-*d*6 at 75 MHz

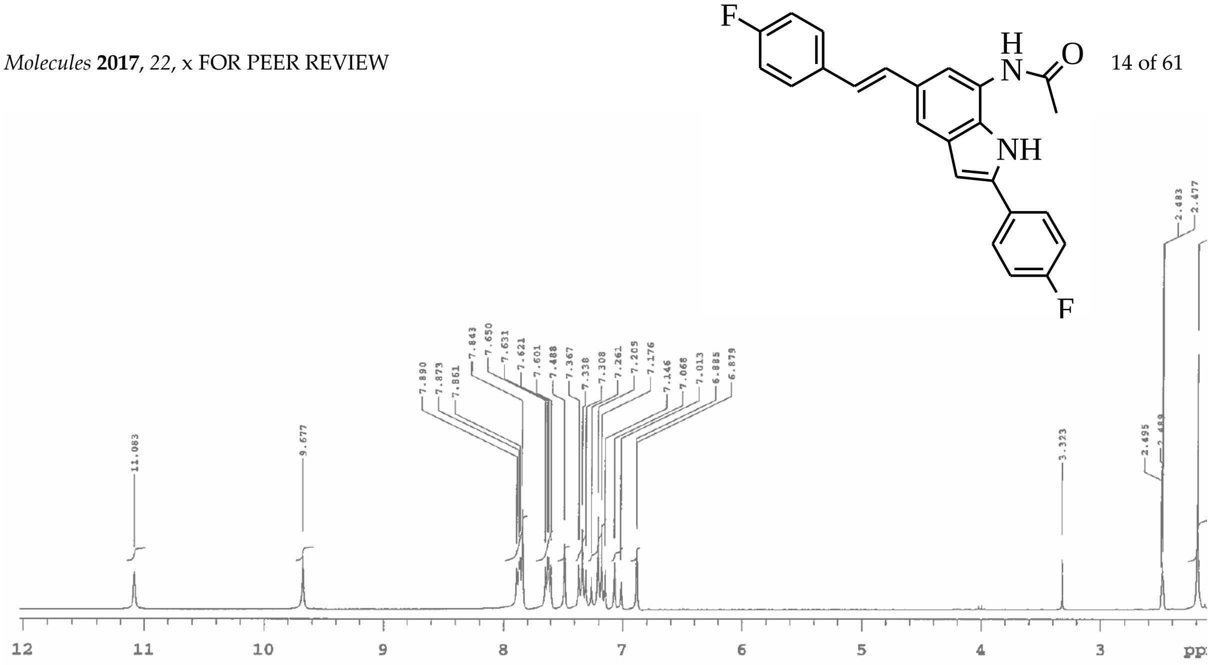

Figure S25. ^{1}H NMR Spectrum of Compound 3f in DMSO-*d_6* at 300 MHz

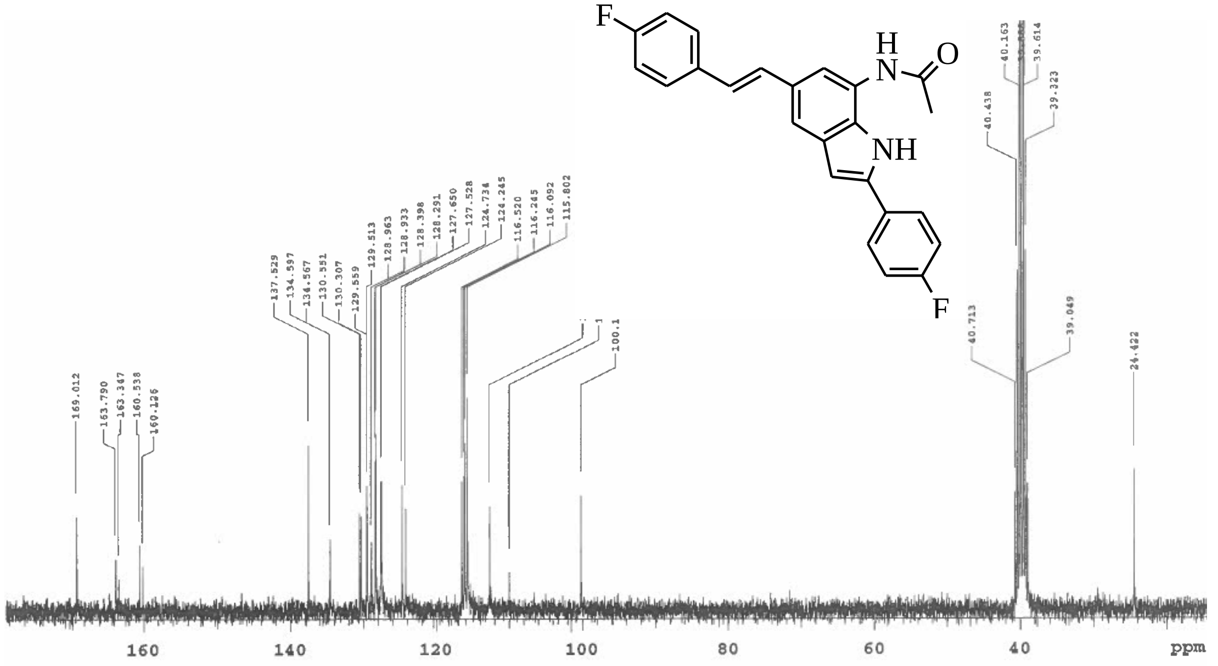

Figure S26. ^{13}C NMR Spectrum of Compound 3f in DMSO-*d_6* at 75 MHz

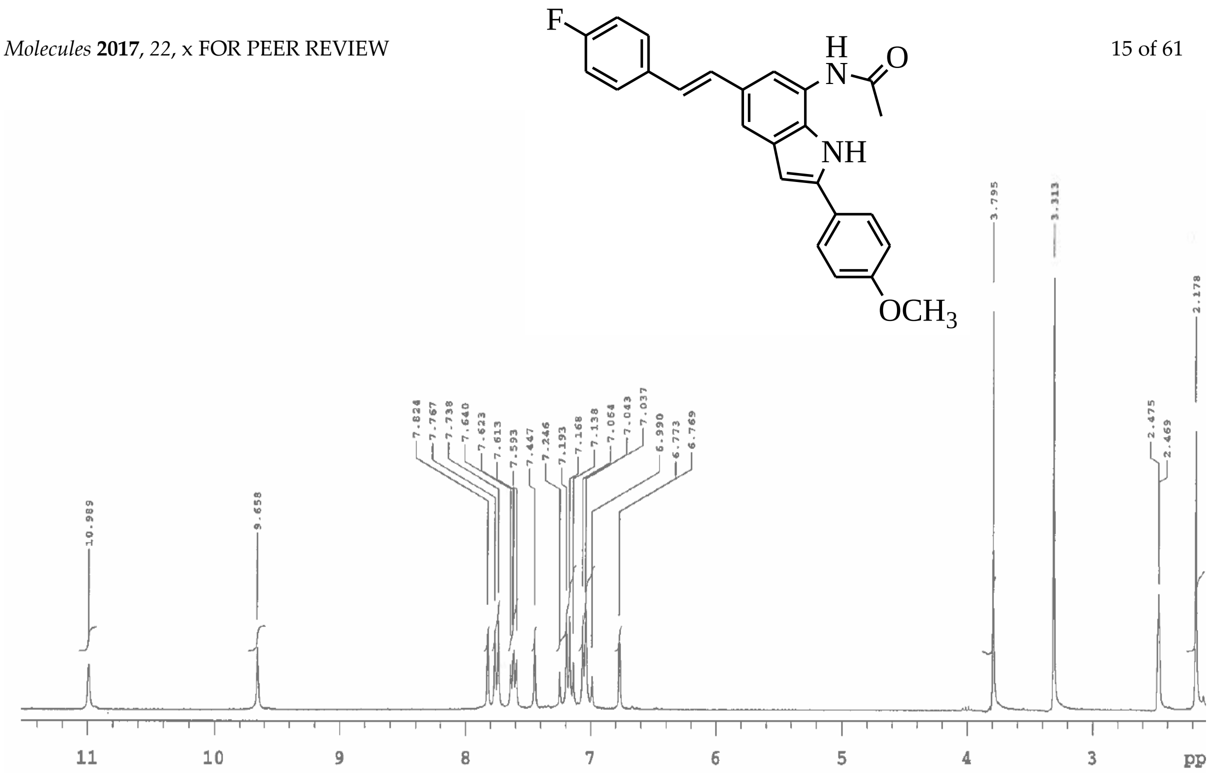

Figure S27. **^{1}H NMR Spectrum of Compound 3g in DMSO-*d6* at 300 MHz**

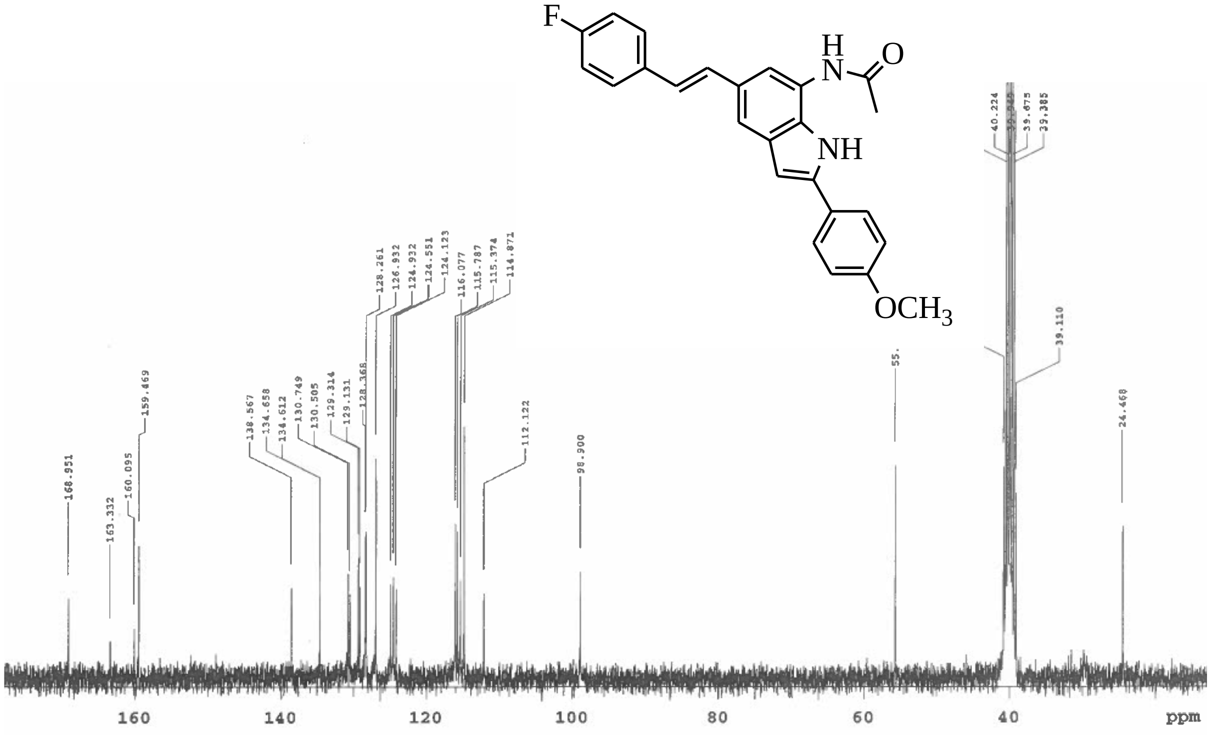

Figure S28. **^{13}C NMR Spectrum of Compound 3g in DMSO-*d6* at 75 MHz**

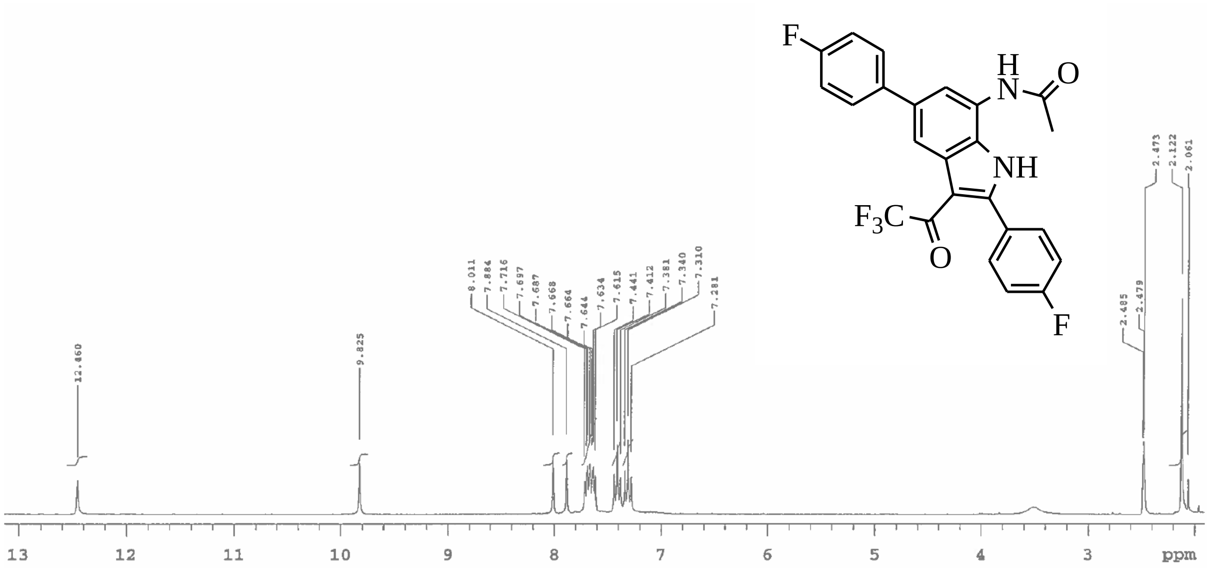

Figure S29. ^{1}H NMR Spectrum of Compound 4a in DMSO-*d*6 at 300 MHz

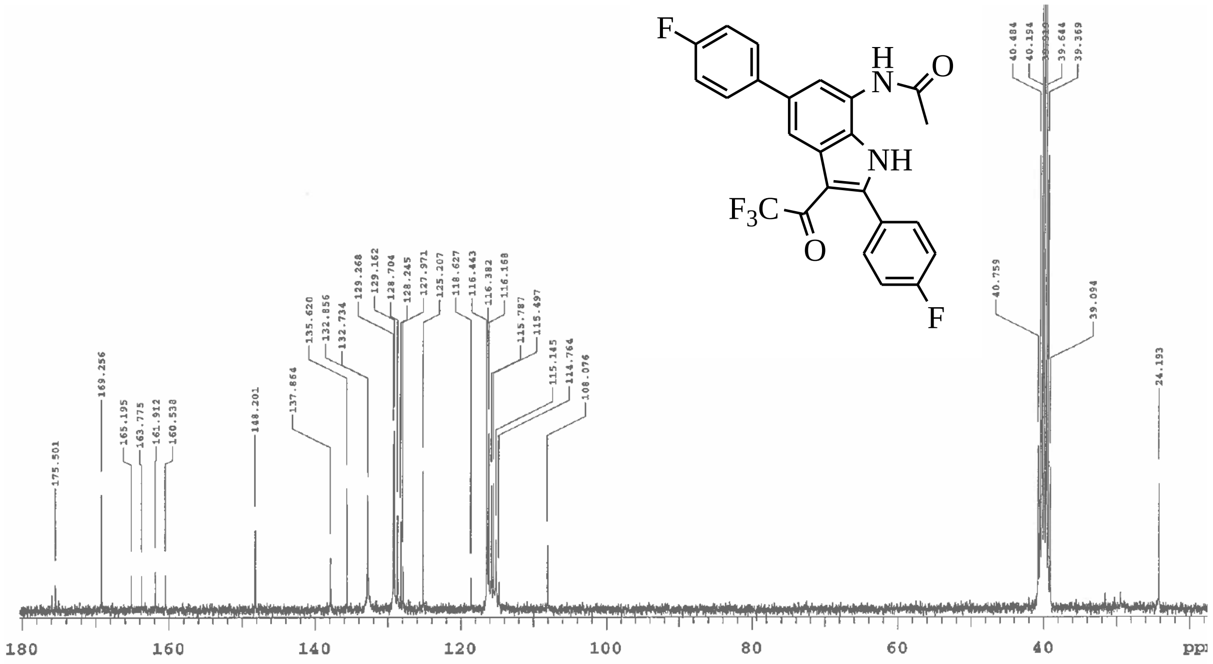

Figure S30. ^{13}C NMR Spectrum of Compound 4a in DMSO-*d*6 at 75 MHz

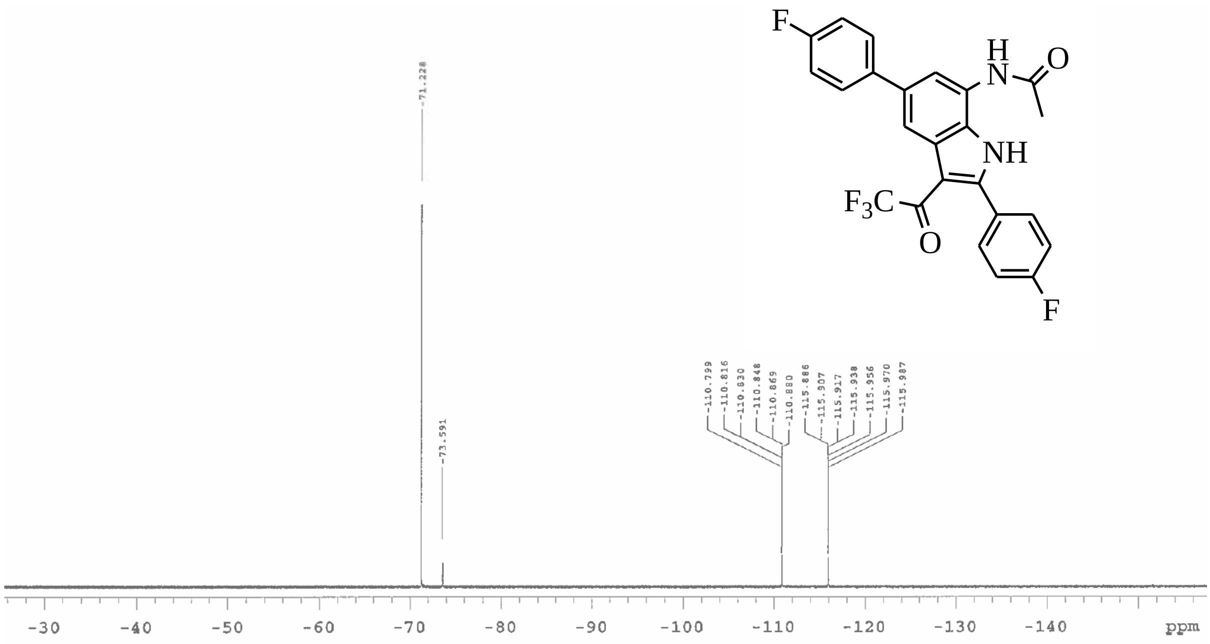

Figure S31. ^{19}**F NMR Spectrum of Compound 4a in DMSO-*d*$_6$ at 282 MHz**

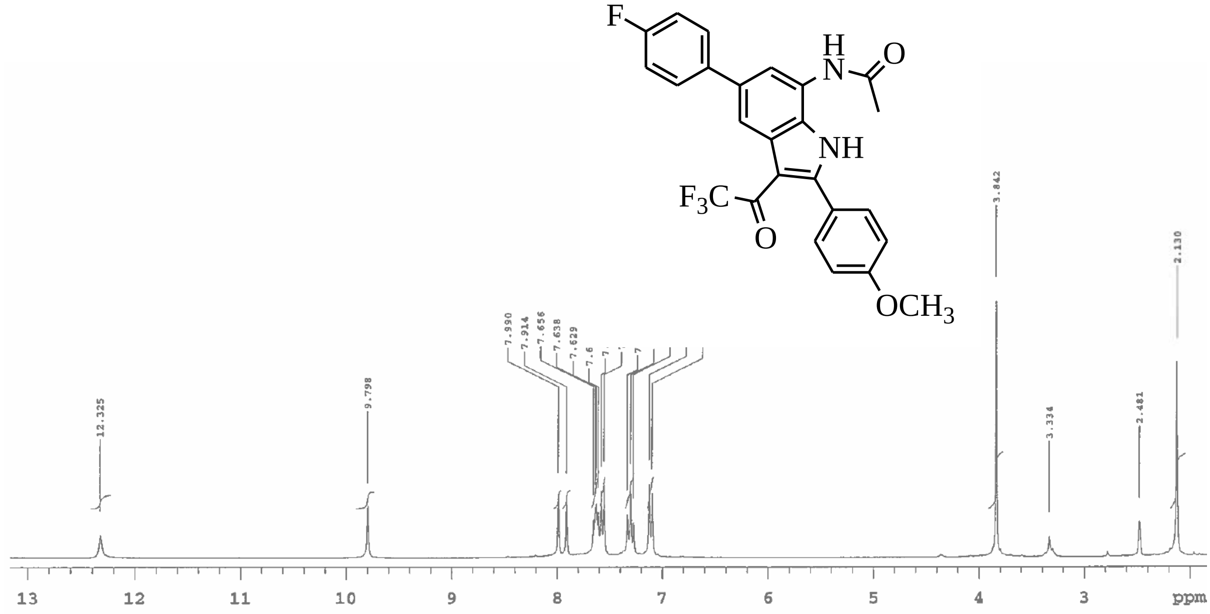

Figure S32. ^{1}**H NMR Spectrum of Compound 4b in DMSO-*d*$_6$ at 300 MHz**

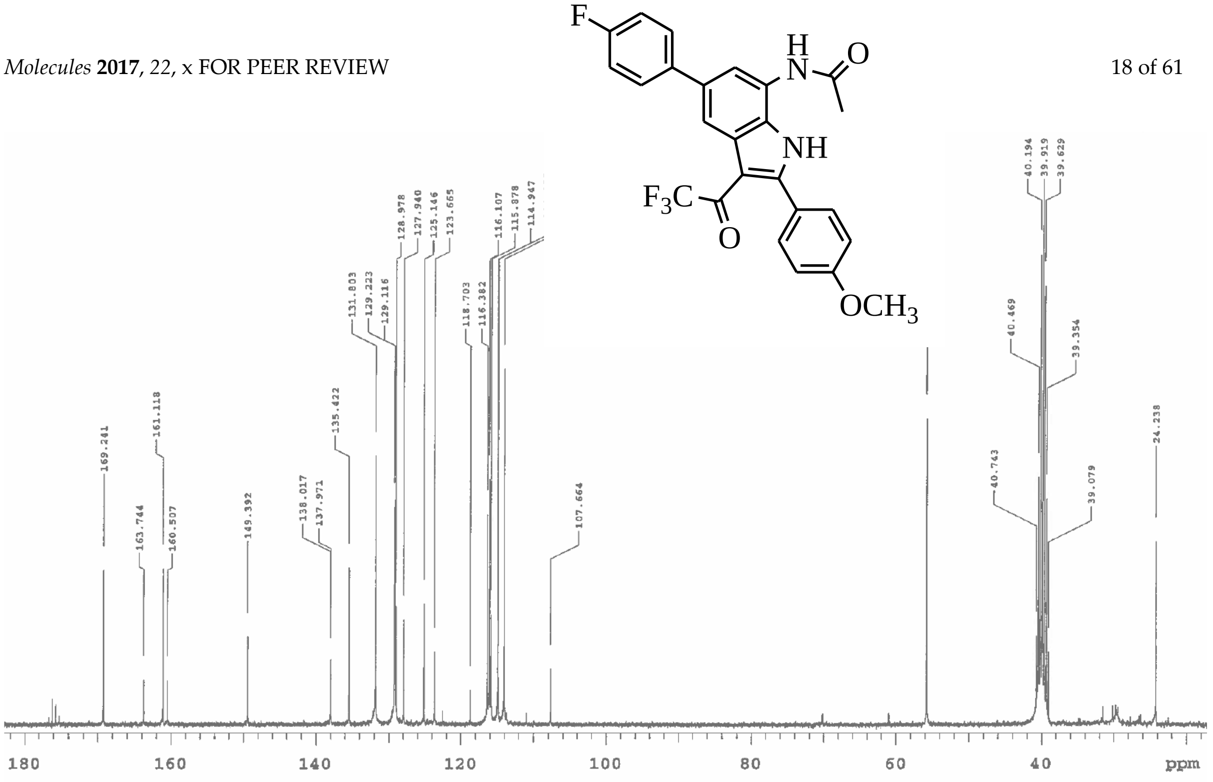

135

136 **Figure S33. ^{13}C NMR Spectrum of Compound 4b in DMSO-*d*$_6$ at 75 MHz**

137

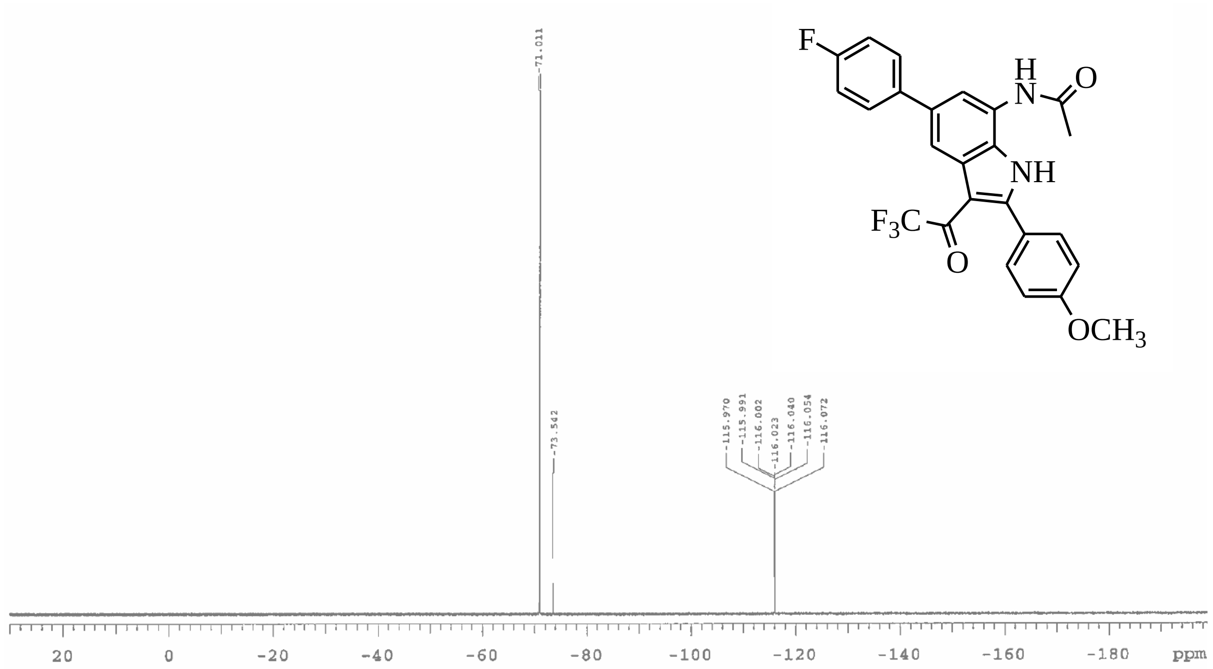

138

139 **Figure S34. ^{19}F NMR Spectrum of Compound 4b in DMSO-*d*$_6$ at 282 MHz**

140

141

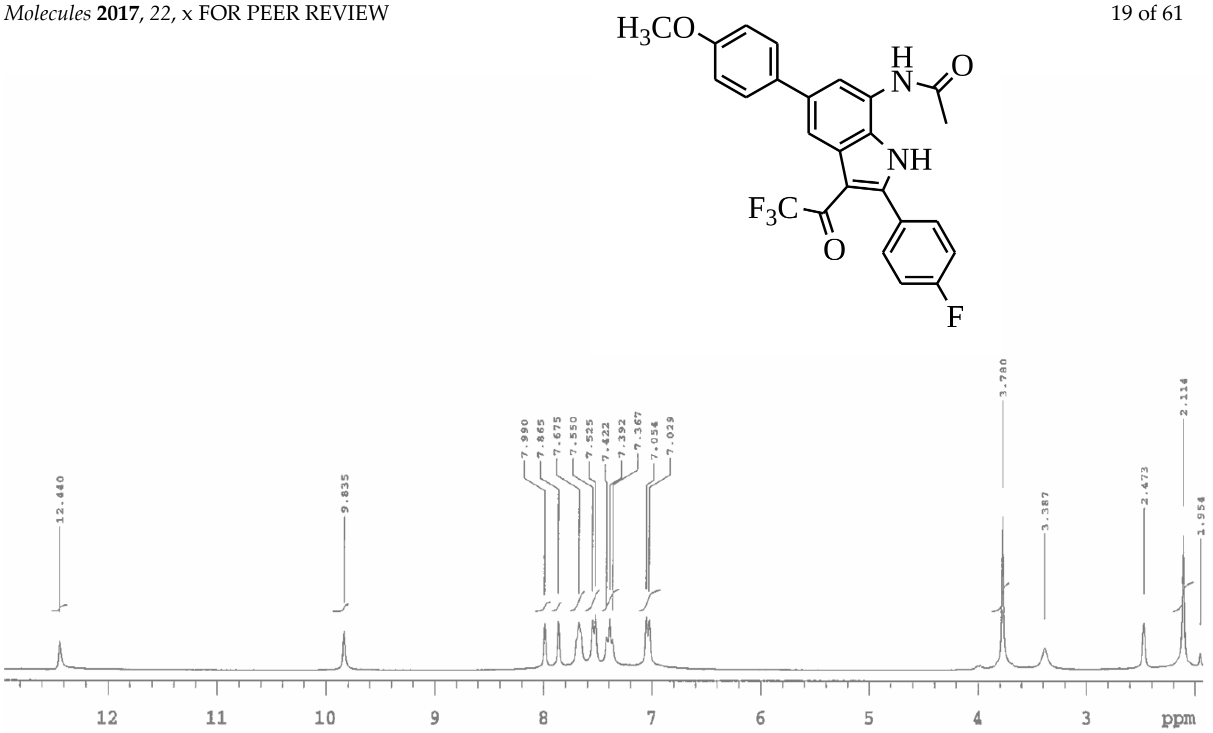

Figure S35. **^{1}H NMR Spectrum of Compound 4c in DMSO-*d*6 at 300 MHz**

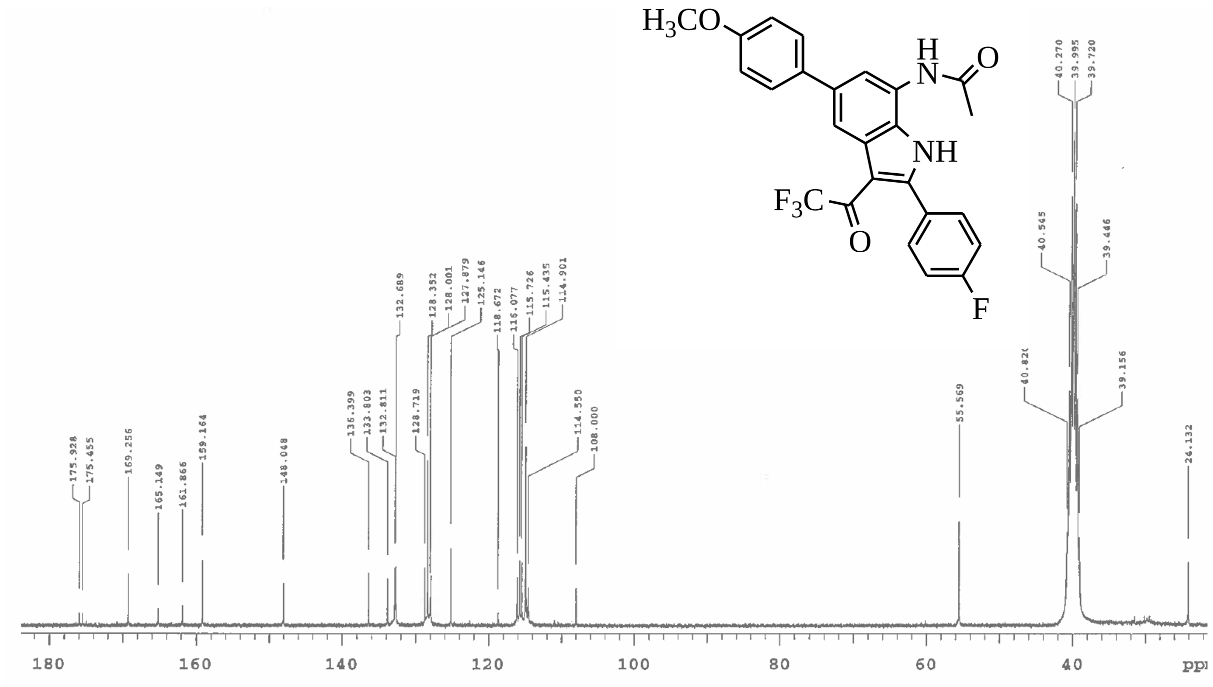

Figure S36. **^{13}C NMR Spectrum of Compound 4c in DMSO-*d*6 at 75 MHz**

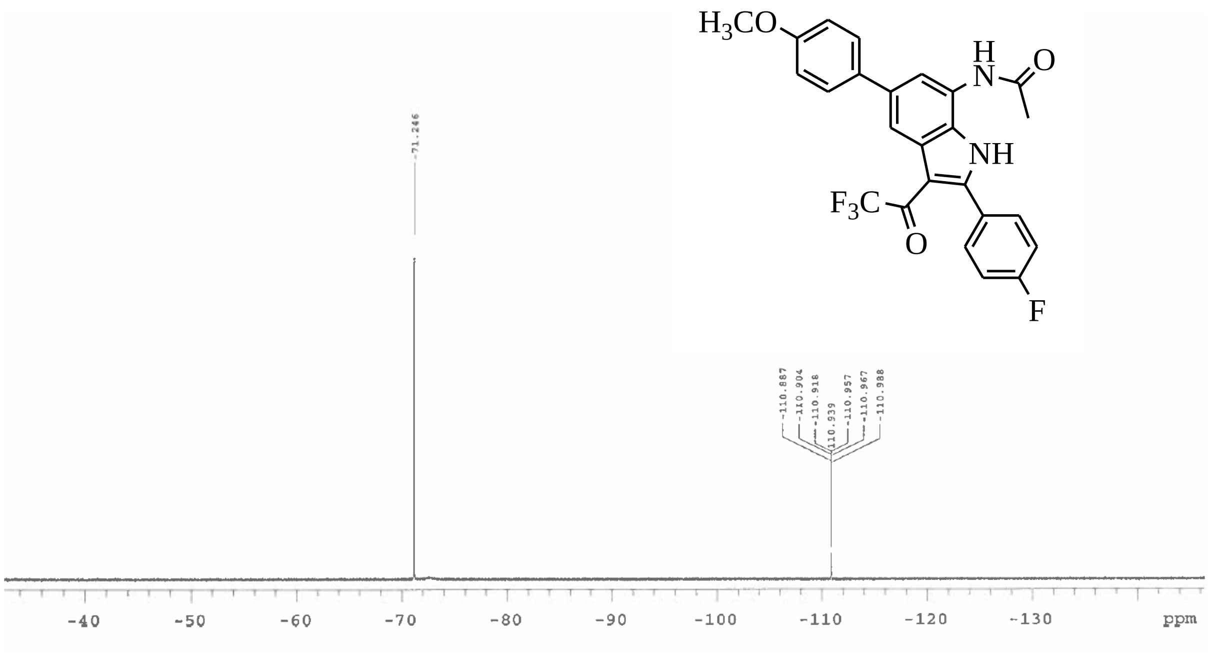

Figure S37. ^{19}**F NMR Spectrum of Compound 4c in DMSO-**d_6 **at 282 MHz**

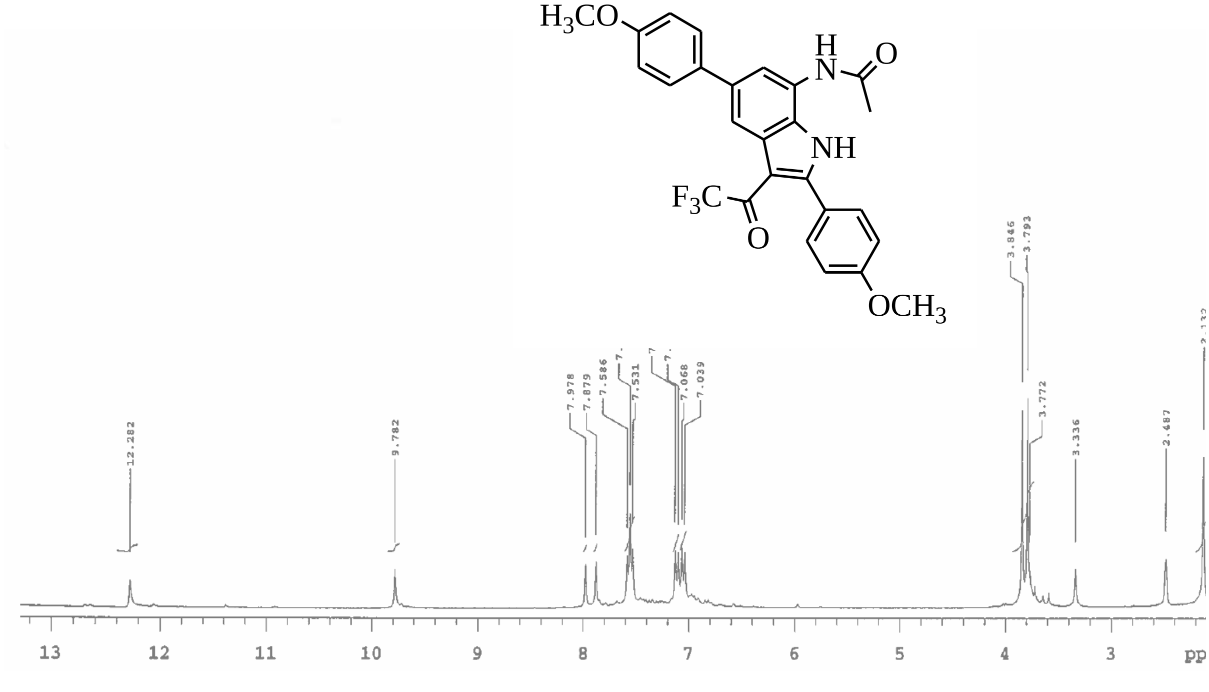

Figure S38. ^{1}**H NMR Spectrum of Compound 4d in DMSO-**d_6 **at 300 MHz**

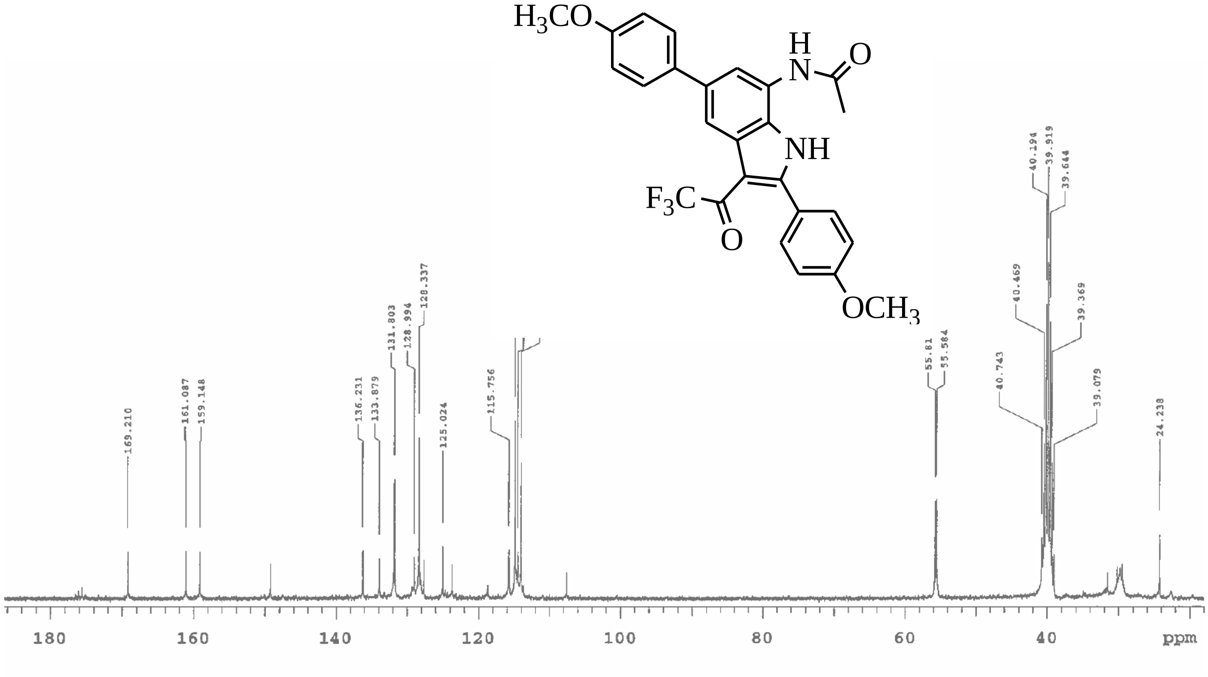

Figure S39. ^{13}C NMR Spectrum of Compound 4d in DMSO-*d*6 at 75 MHz

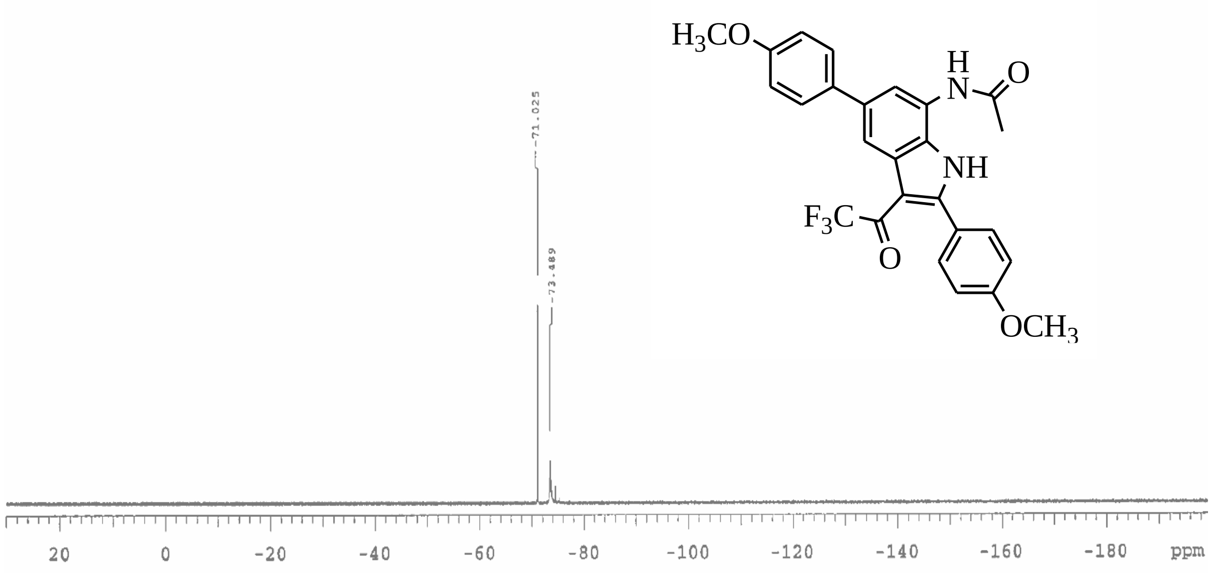

Figure S40. ^{19}F NMR Spectrum of Compound 4d in DMSO-*d*6 at 282 MHz

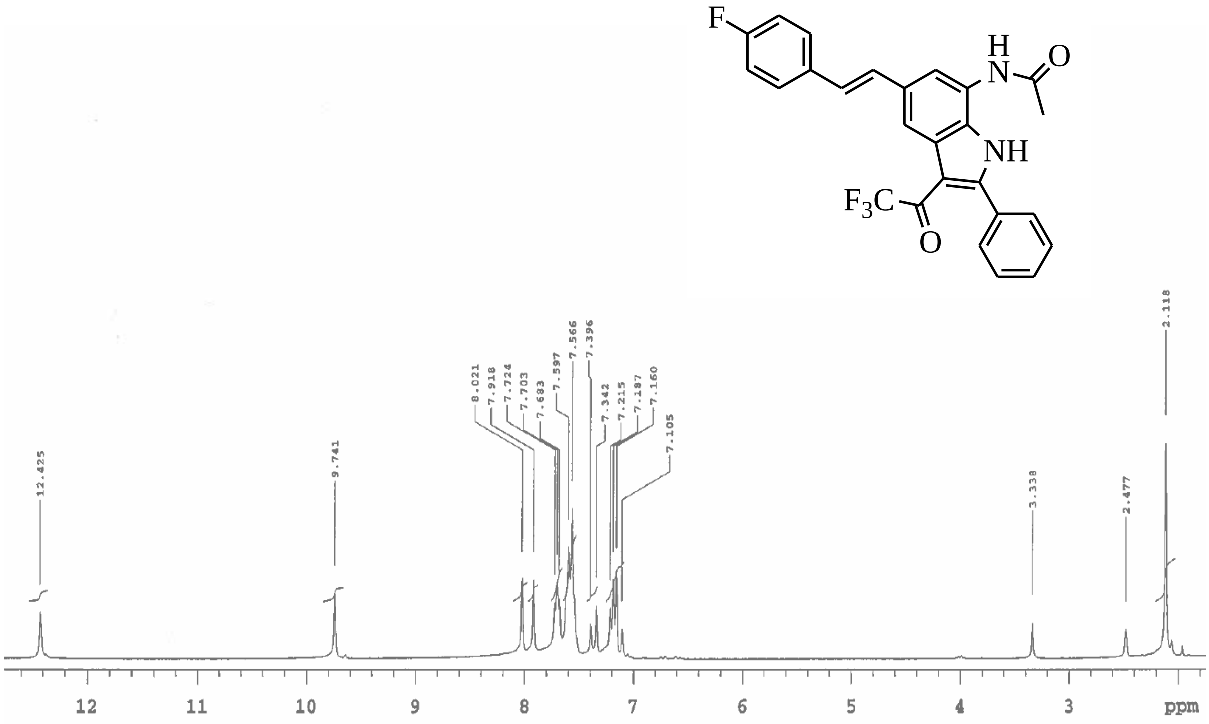

Figure S41. ^{1}H NMR Spectrum of Compound 4e in DMSO-*d*$_6$ at 300 MHz

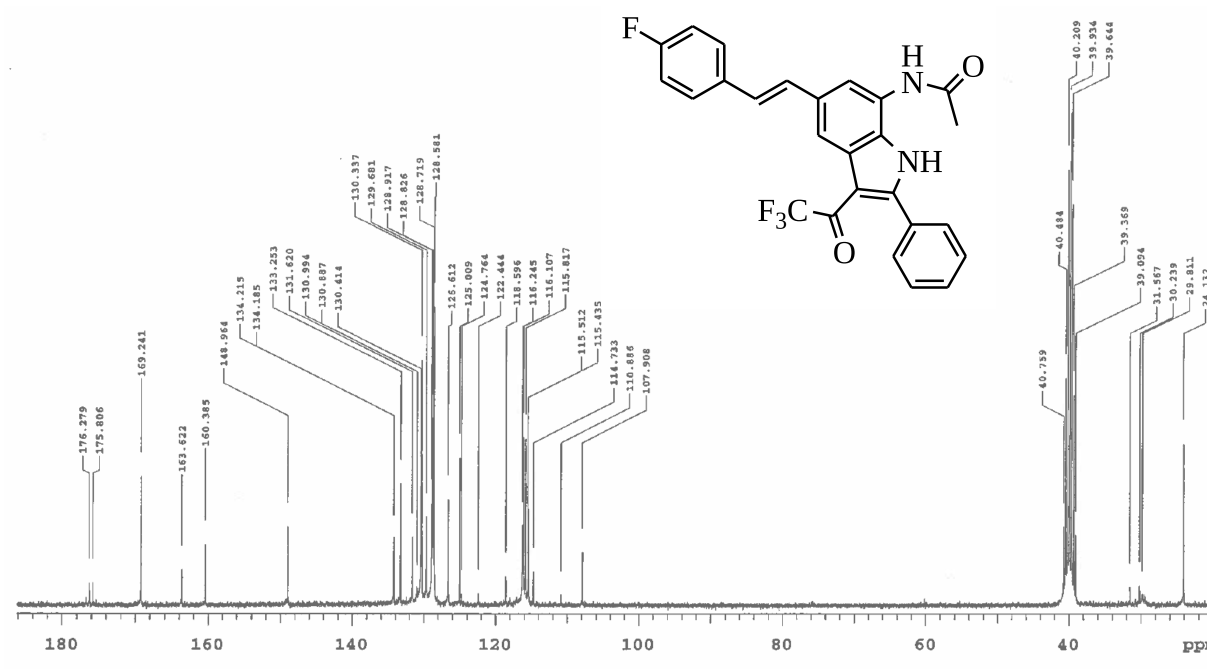

Figure S42. ^{13}C NMR Spectrum of Compound 4e in DMSO-*d*$_6$ at 75 MHz

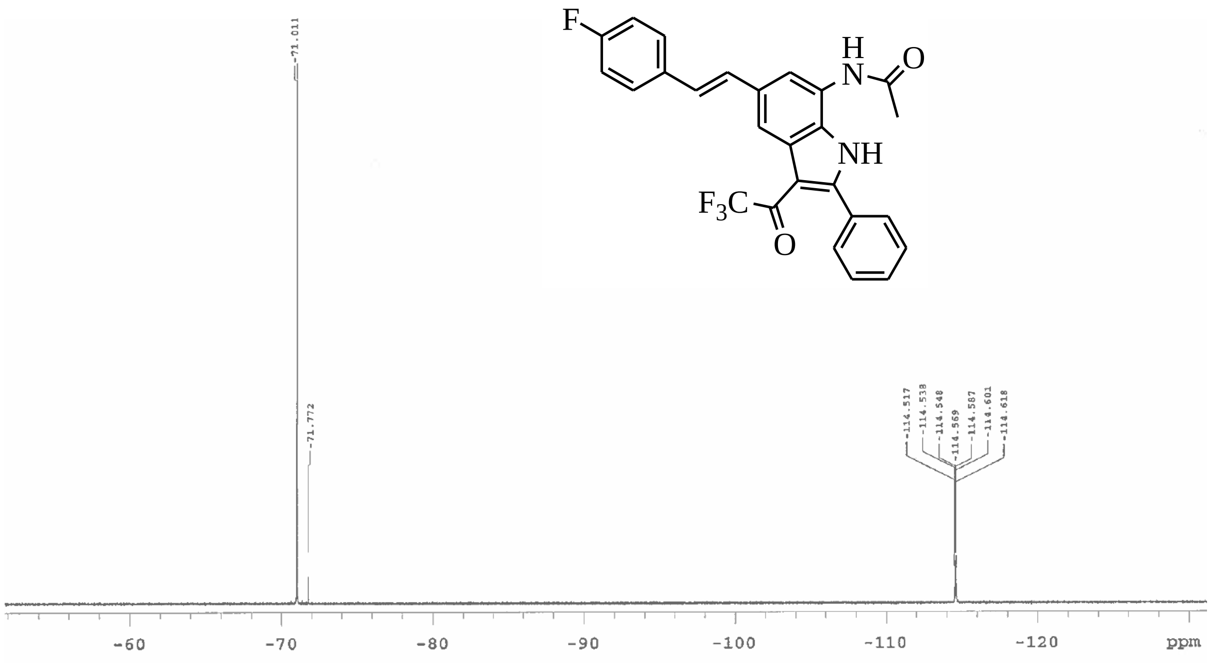

Figure S43. ^{19}F NMR Spectrum of Compound 4e in DMSO-*d*$_6$ at 282 MHz

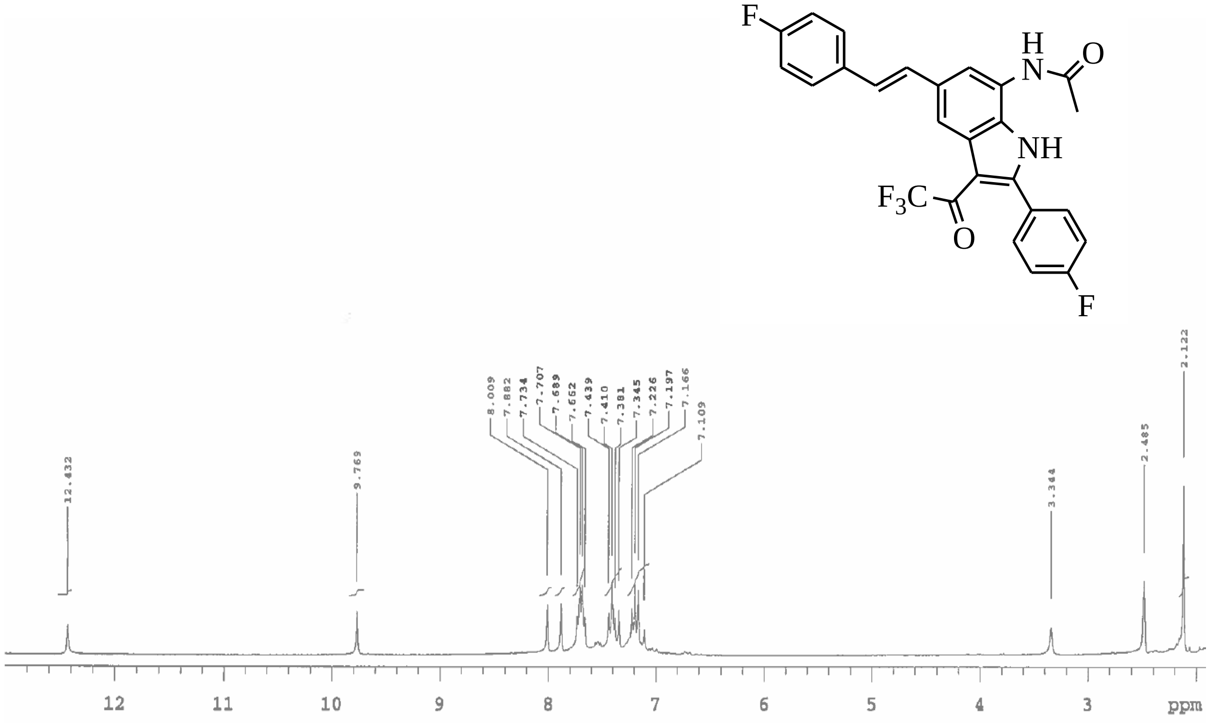

Figure S44. ^{1}H NMR Spectrum of Compound 4f in DMSO-*d*$_6$ at 300 MHz

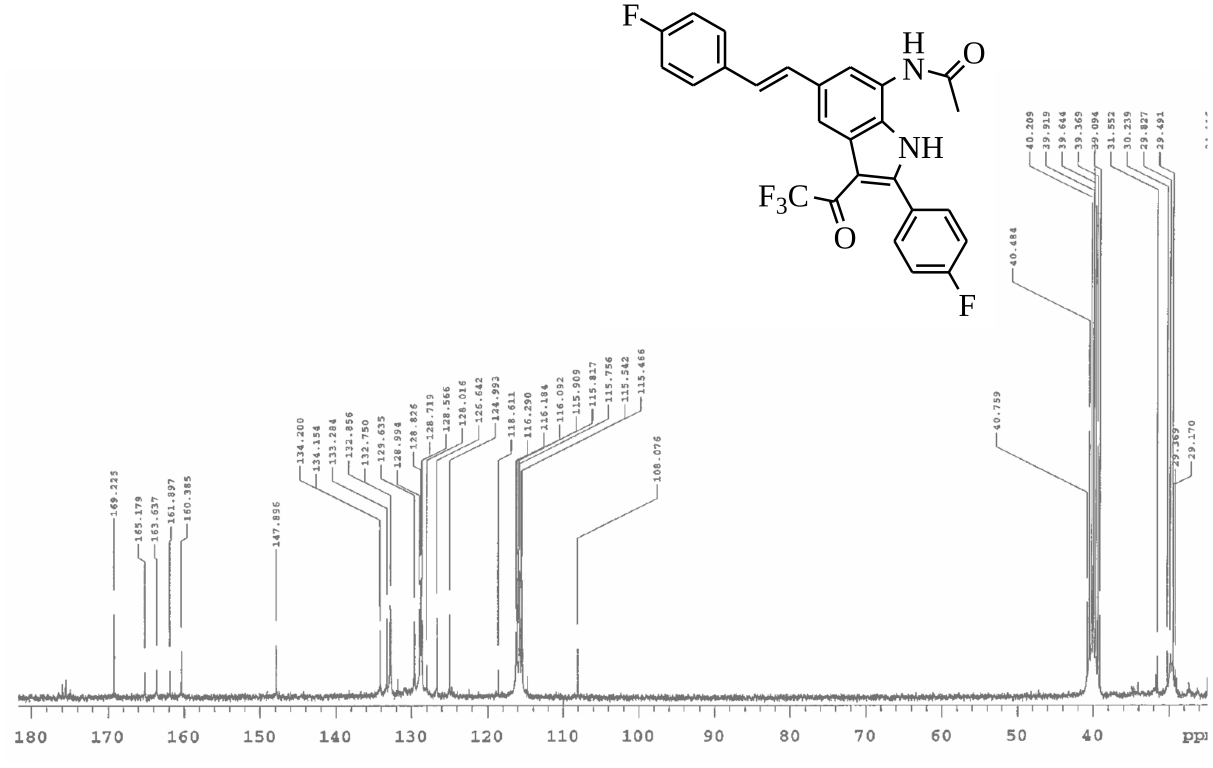

Figure S45. **^{13}C NMR Spectrum of Compound 4f in DMSO-*d*$_6$ at 75 MHz**

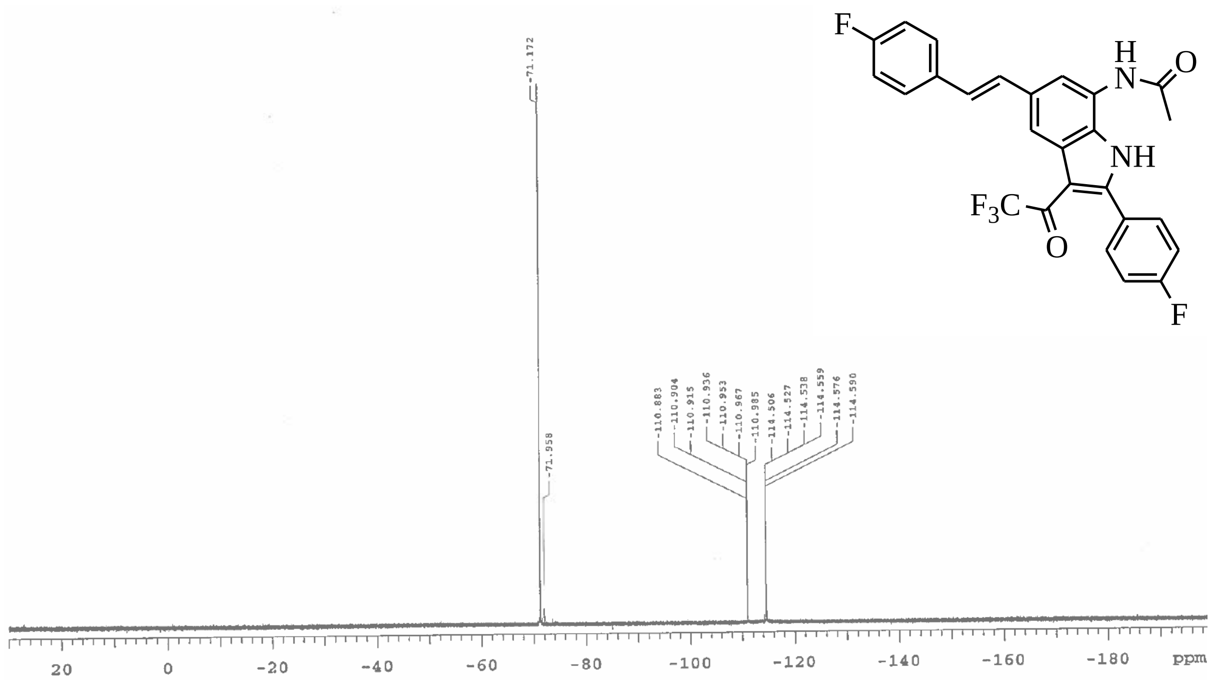

Figure S46. **^{19}F NMR Spectrum of Compound 4f in DMSO-*d*$_6$ at 282 MHz**

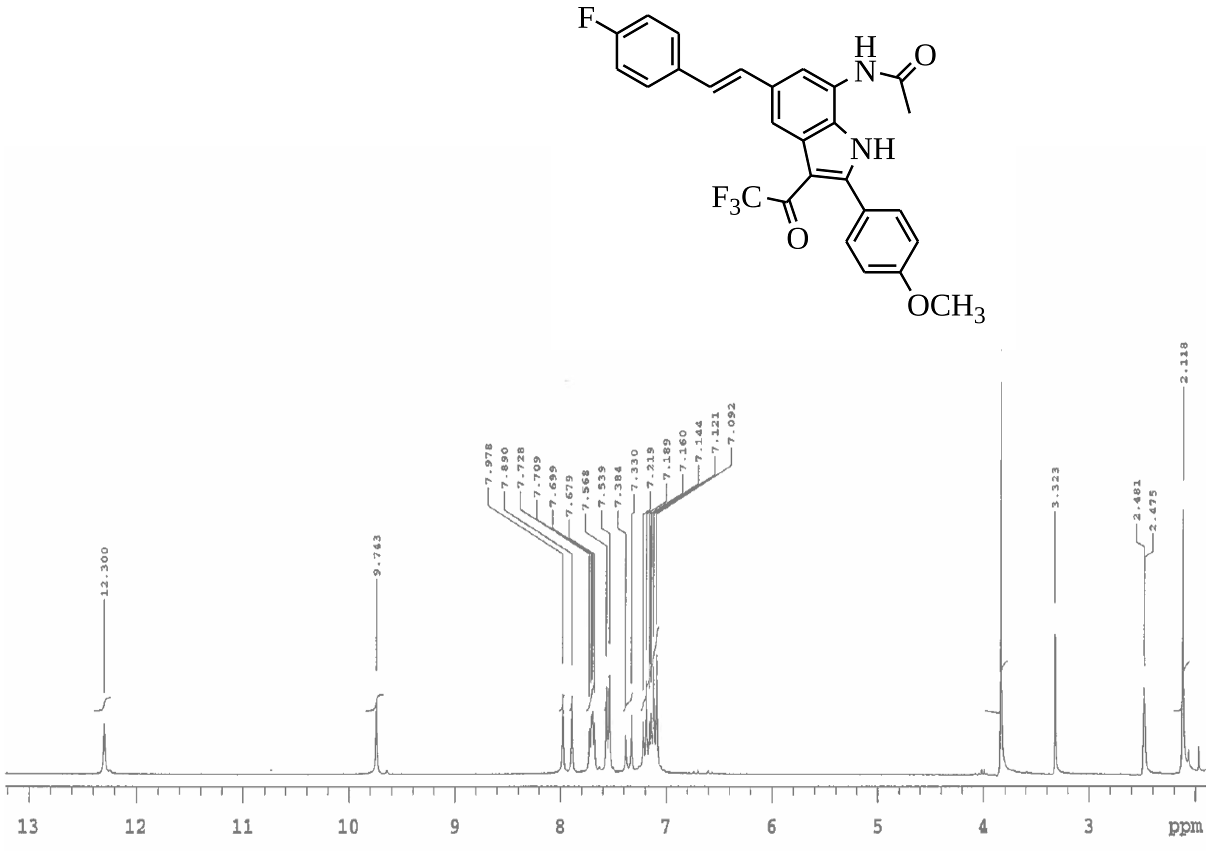

Figure S47. ^{1}H NMR Spectrum of Compound 4g in DMSO-*d₆* at 300 MHz

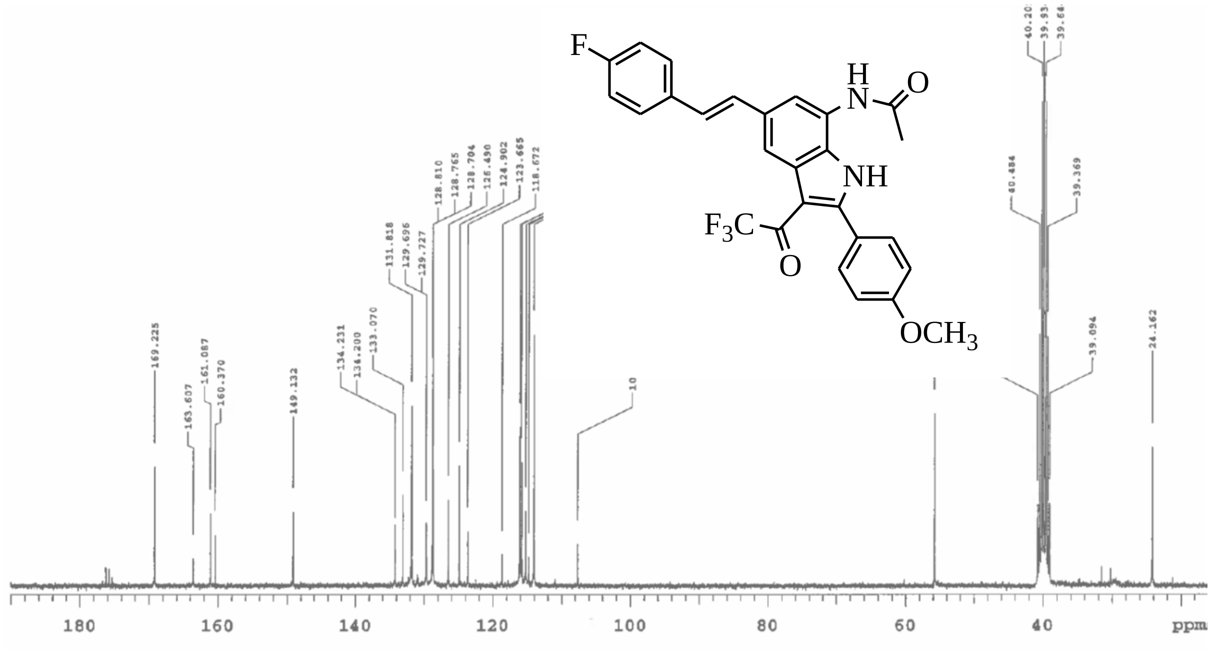

Figure S48. ^{13}C NMR Spectrum of Compound 4g in DMSO-*d₆* at 75 MHz

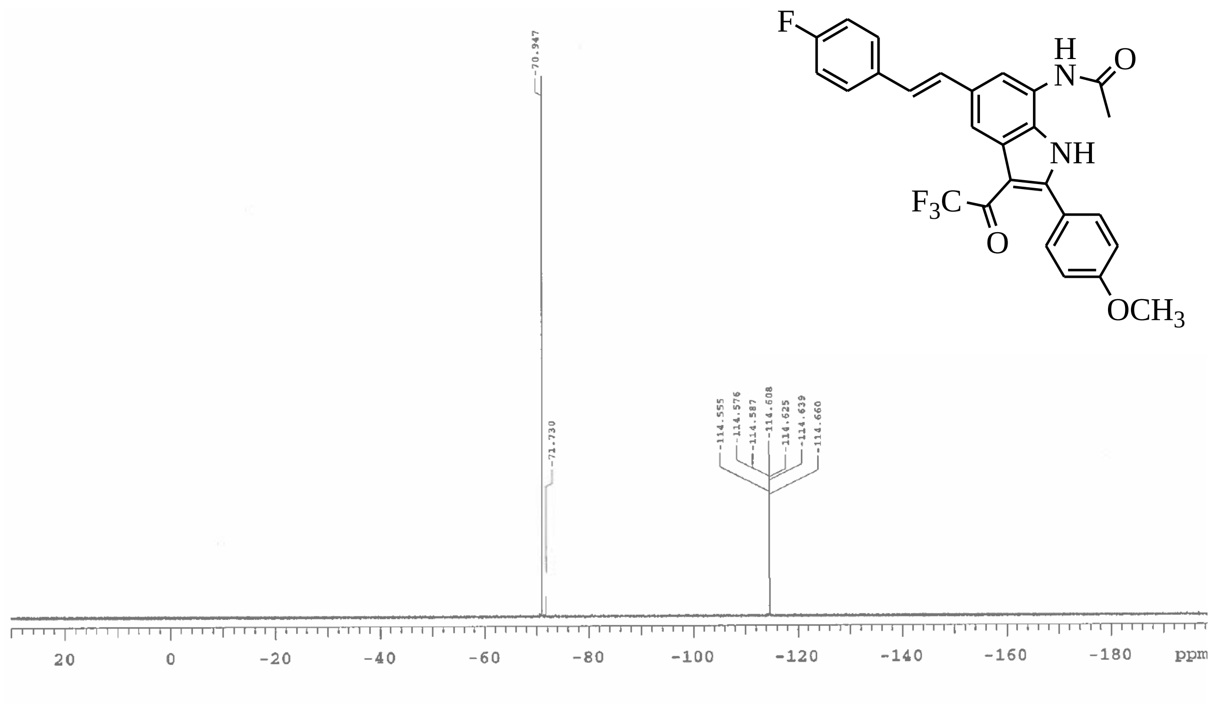

Figure S49. ^{19}F NMR Spectrum of Compound 4g in DMSO-*d*$_6$ at 282 MHz

Supplementary 2: % cell viability and LC_{50} values of chloroquine and compounds 3 and 4

Table 1. Log solvent percentage vs. % Parasite survival data used to plot dose-response curve for DMSO.

Log Solv. Perc (%)[a]	DMSO	
	%PS	SD
**0.00**	91.41	8.66
**-0.48**	101.62	5.42
**-0.96**	107.17	1.17
**-1.43**	100.72	5.58
**-1.92**	99.56	7.82
**-2.39**	106.94	5.85
**-2.85**	102.51	1.52
**-3.33**	121.92	5.28
**-3.82**	103.65	6.05
**-4.29**	109.18	6.16

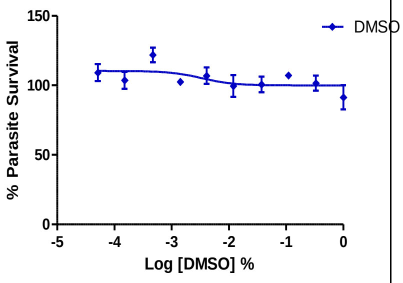

[a]Data are expressed as means of triplicate values. SD = standard deviation; %PS = Percentage Parasite Survival.

Table 2. Log concentration vs. % Parasite survival data used to plot dose-response curve for chloroquine

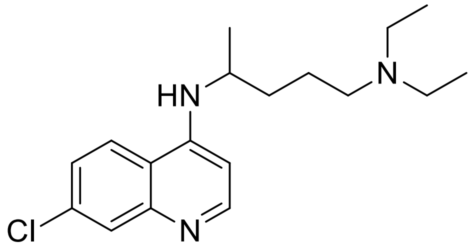

Chloroquine

Figure S50. Chemical structure of chloroquine

.Log Conc (nM)[a]	CQ	
	%PS	SD
**3.000**	0.11	0.20
**2.520**	0.00	0.00
**2.040**	1.21	2.10
**1.560**	0.00	0.00
**1.090**	32.89	2.13
**0.614**	60.87	2.79
**0.137**	94.99	2.90
**-0.340**	95.42	6.61
**-0.818**	98.51	6.29
**-1.294**	108.04	8.58

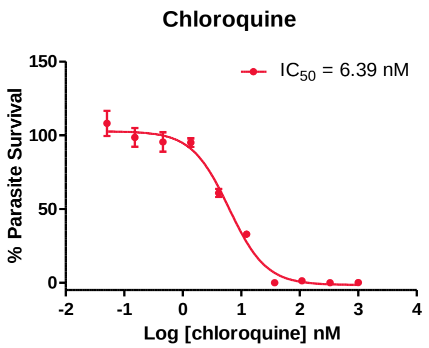

[a]Data are expressed as means of triplicate values. SD = standard deviation; %PS = Percentage Parasite Survival.

Table 3. Log concentration vs. % Parasite survival data used to plot dose-response curve for **3a**.

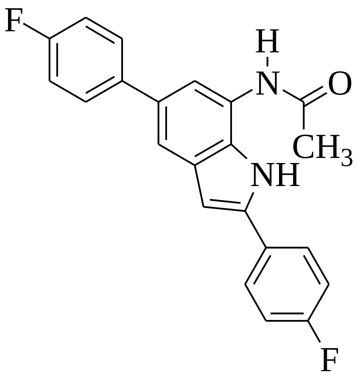

3a

Figure S51. Chemical structure of **3a**

Log Conc (µM)[a]	Mak010	
	%PS	SD
**2.00**	0.00	0.00
**1.52**	0.00	0.00
**1.04**	0.00	0.00
**0.57**	15.64	1.59
**0.09**	48.11	5.47
**-0.39**	74.07	1.57
**-0.86**	74.96	7.75
**-1.34**	89.87	9.82
**-1.82**	95.34	8.94
**-2.29**	92.04	6.29

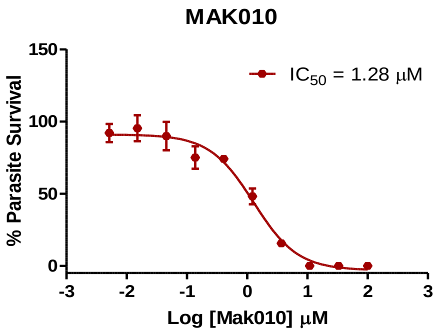

[a]Data are expressed as means of triplicate values. SD = standard deviation; %PS = Percentage Parasite Survival.

Table 4. Log concentration vs. % Parasite survival data used to plot dose-response curve for **3b**.

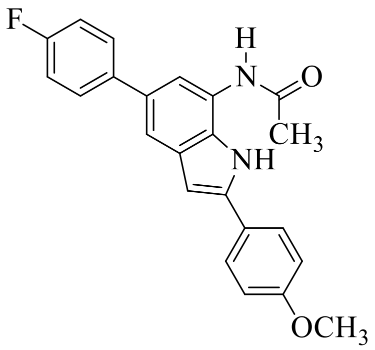

3b

Figure S52. Chemical structure of **3b**

Log Conc (µM)[a]	Mak011	
	%PS	SD
**2.00**	0.00	0.00
**1.52**	0.00	0.00
**1.04**	0.00	0.00
**0.57**	71.29	4.66
**0.09**	80.98	10.32
**-0.39**	86.71	11.94
**-0.86**	94.49	7.96
**-1.34**	93.10	9.89
**-1.82**	99.18	12.64
**-2.29**	98.70	4.40

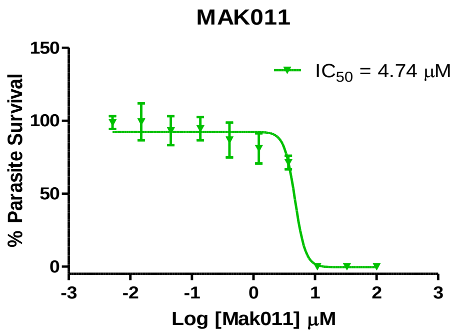

[a]Data are expressed as means of triplicate values. SD = standard deviation; %PS = Percentage Parasite Survival.

Table 5. Log concentration vs. % Parasite survival data used to plot dose-response curve for **3c**.

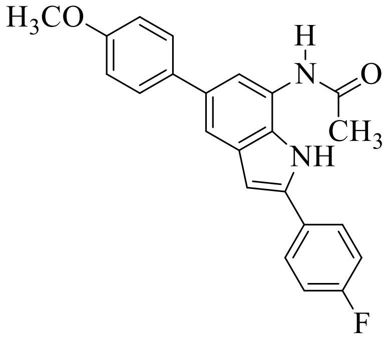

3c

Figure S53. Chemical structure of **3c**

Log Conc (nM)[a]	Mak012	
	%PS	SD
**2.00**	0.00	0.00
**1.52**	0.00	0.00
**1.04**	19.52	1.14
**0.57**	77.74	3.97
**0.09**	89.50	14.79
**-0.39**	93.80	3.17
**-0.86**	91.69	2.31
**-1.34**	90.90	3.20
**-1.82**	93.13	2.22
**-2.29**	102.94	4.69

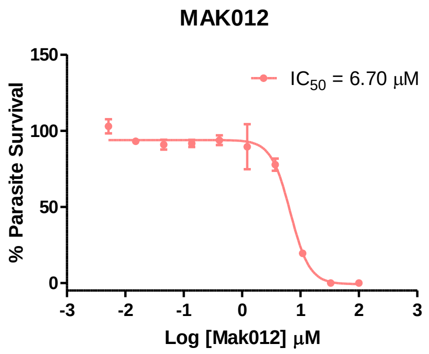

[a]Data are expressed as means of triplicate values. SD = standard deviation; %PS = Percentage Parasite Survival.

Table 6. Log concentration vs. % Parasite survival data used to plot dose-response curve for **3d**.

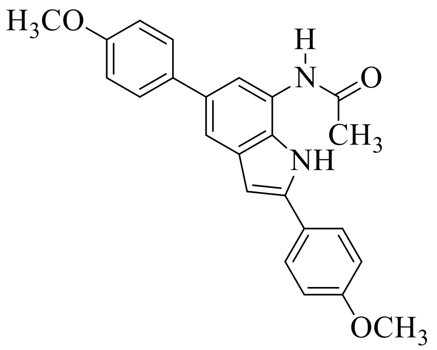

3d

Figure S54. Chemical structure of **3d**

Log Conc (nM)[a]	Mak013	
	%PS	SD
**2.00**	1.29	2.24
**1.52**	6.22	0.66
**1.04**	39.93	3.90
**0.57**	104.47	2.71
**0.09**	103.42	6.94
**-0.39**	107.59	2.04
**-0.86**	103.72	8.42
**-1.34**	109.86	2.87
**-1.82**	105.85	3.24
**-2.29**	105.83	3.62

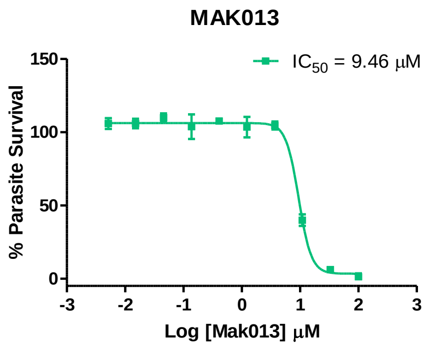

[a]Data are expressed as means of triplicate values. SD = standard deviation; %PS = Percentage Parasite Survival.

Table 7. Log concentration vs. % Parasite survival data used to plot dose-response curve for **3e**.

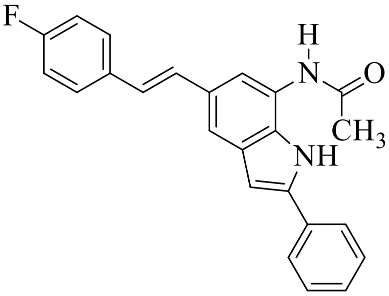

3e

Figure S55. Chemical structure of **3e**

Log Conc (nM)[a]	Mak014	
	%PS	SD
**2.00**	0.00	0.00
**1.52**	0.00	0.00
**1.04**	21.22	7.20
**0.57**	58.26	3.47
**0.09**	79.97	8.12
**-0.39**	100.22	7.27
**-0.86**	108.57	4.25
**-1.34**	98.49	6.72
**-1.82**	115.28	0.79
**-2.29**	109.42	5.80

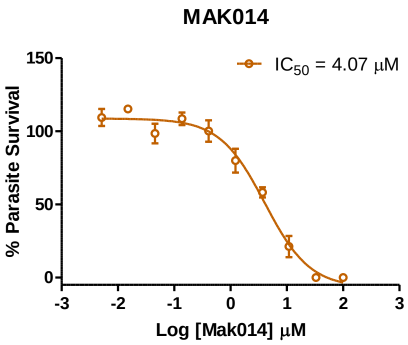

[a]Data are expressed as means of triplicate values. SD = standard deviation; %PS = Percentage Parasite Survival.

Table 8. Log concentration vs. % Parasite survival data used to plot dose-response curves for **3f**.

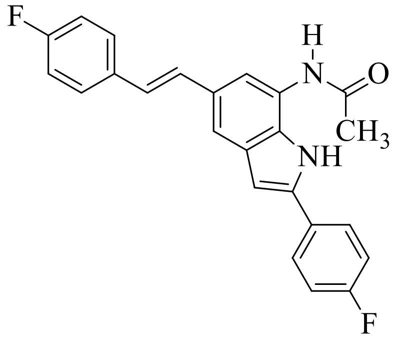

3f

Figure S56. Chemical structure of **3f**

Log Conc (nM)[a]	Mak015	
	%PS	SD
**2.00**	0.00	0.00
**1.52**	0.00	0.00
**1.04**	0.00	0.00
**0.57**	21.48	2.58
**0.09**	66.49	3.66
**-0.39**	100.45	3.36
**-0.86**	102.03	3.69
**-1.34**	96.58	3.50
**-1.82**	107.12	5.16
**-2.29**	100.44	13.95

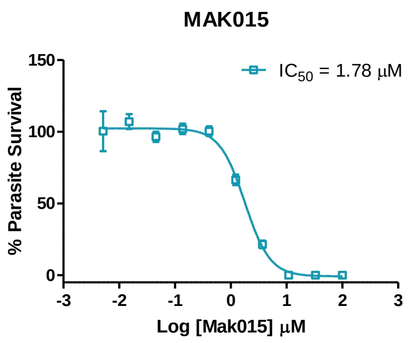

[a]Data are expressed as means of triplicate values. SD = standard deviation; %PS = Percentage Parasite Survival.

Table 9. Log concentration vs. % Parasite survival data used to plot dose-response curve for **3g**.

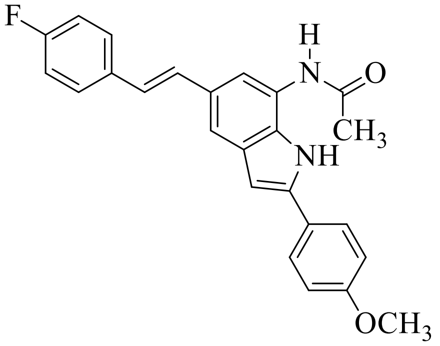

3g

Figure S57. Chemical structure of **3g**

**Log Conc (nM)**[a]	**Mak016**	
	**%PS**	**SD**
**2.00**	0.00	0.00
**1.52**	0.00	0.00
**1.04**	60.43	3.21
**0.57**	89.42	2.84
**0.09**	80.58	5.76
**-0.39**	91.40	4.44
**-0.86**	85.19	2.22
**-1.34**	87.26	7.30
**-1.82**	97.74	3.83
**-2.29**	95.92	0.24

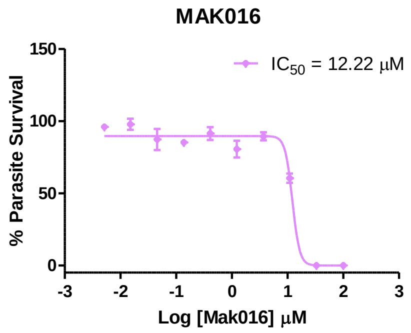

[a]Data are expressed as means of triplicate values. SD = standard deviation; %PS = Percentage Parasite Survival.

Table 10. Log concentration vs. % Parasite survival data used to plot dose-response curve for **4a**.

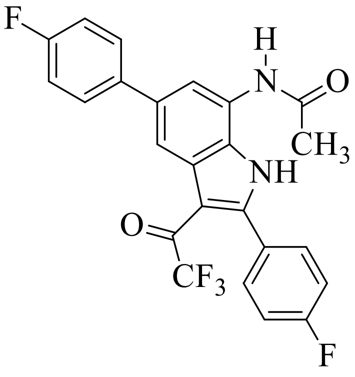

4a

Figure S58. Chemical structure of **4a**

Log Conc (nM)[a]	Mak017	
	%PS	SD
**2.00**	0.00	0.00
**1.52**	0.00	0.00
**1.04**	0.00	0.00
**0.57**	11.72	2.10
**0.09**	65.14	2.55
**-0.39**	72.07	2.54
**-0.86**	77.87	1.99
**-1.34**	87.25	6.26
**-1.82**	93.96	1.95
**-2.29**	93.73	8.60

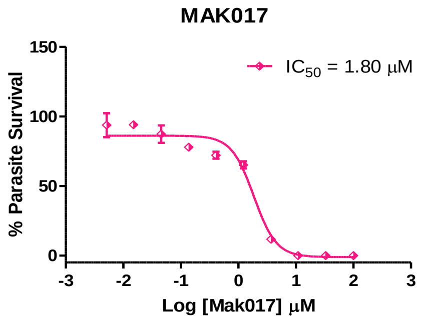

[a]Data are expressed as means of triplicate values. SD = standard deviation; %PS = Percentage Parasite Survival.

Table 11. Log concentration vs. % Parasite survival data used to plot dose-response curve for **4b**.

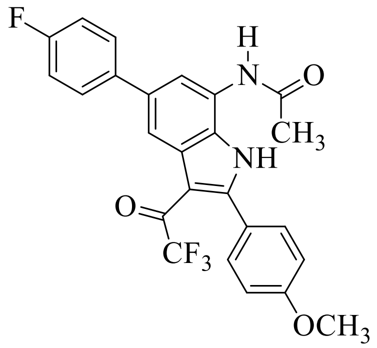

4b

Figure S59. Chemical structure of **4b**

Log Conc (nM)[a]	Mak018	
	%PS	SD
**2.00**	0.00	0.00
**1.52**	0.00	0.00
**1.04**	0.00	0.00
**0.57**	56.59	6.18
**0.09**	91.05	8.86
**-0.39**	92.10	10.32
**-0.86**	95.97	12.54
**-1.34**	94.36	15.51
**-1.82**	94.82	17.88
**-2.29**	95.33	0.31

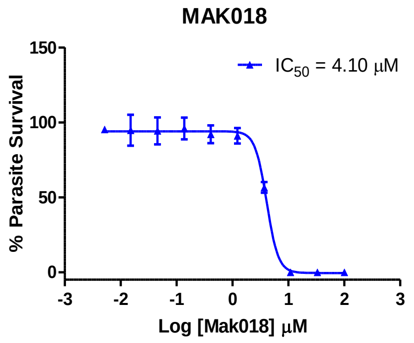

[a]Data are expressed as means of triplicate values. SD = standard deviation; %PS = Percentage Parasite Survival.

Table 12. Log concentration vs. % Parasite survival data used to plot dose-response curves for **4c**.

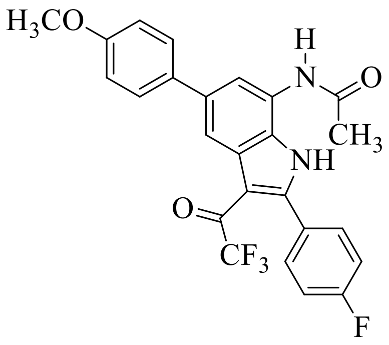

4c

Figure S60. Chemical structure of **4c**

Log Conc (nM)[a]	Mak019	
	%PS	SD
**2.00**	0.00	0.00
**1.52**	0.00	0.00
**1.04**	0.00	0.00
**0.57**	61.91	1.75
**0.09**	85.74	6.81
**-0.39**	103.78	13.62
**-0.86**	98.19	10.00
**-1.34**	102.00	8.27
**-1.82**	101.10	8.85
**-2.29**	94.61	3.19

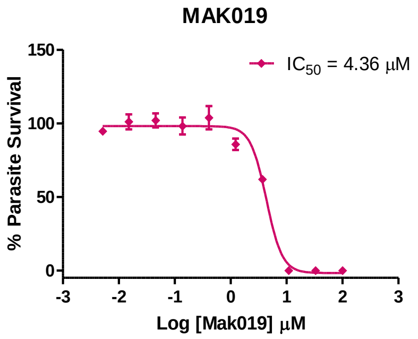

[a]Data are expressed as means of triplicate values. SD = standard deviation; %PS = Percentage Parasite Survival.

Table 13. Log concentration vs. % Parasite survival data used to plot dose-response curve for **4d**.

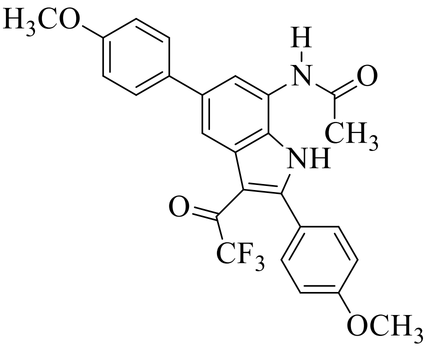

4d

Figure S61. Chemical structure of **4d**

Log Conc (nM)[a]	Mak020	
	%PS	SD
**2.00**	0.00	0.00
**1.52**	0.00	0.00
**1.04**	28.38	8.73
**0.57**	79.58	3.47
**0.09**	81.36	6.46
**-0.39**	96.45	18.29
**-0.86**	82.93	7.17
**-1.34**	86.61	4.56
**-1.82**	85.55	1.87
**-2.29**	85.53	4.67

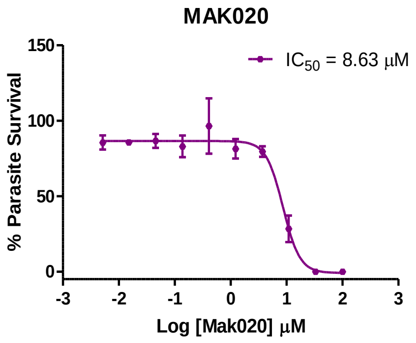

[a]Data are expressed as means of triplicate values. SD = standard deviation; %PS = Percentage Parasite Survival.

Table 14. Log concentration vs. % Parasite survival data used to plot dose-response curve for **4e**.

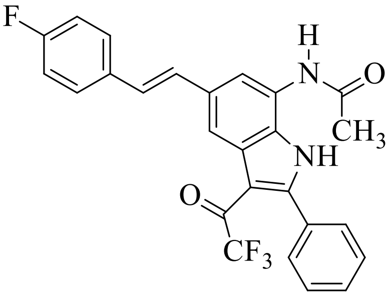

4e

Figure S62. Chemical structure of **4e**

Log Conc (nM)[a]	Mak021	
	%PS	SD
**2.00**	0.00	0.00
**1.52**	0.00	0.00
**1.04**	0.00	0.00
**0.57**	30.89	6.27
**0.09**	103.39	4.80
**-0.39**	103.65	7.91
**-0.86**	110.72	19.57
**-1.34**	108.71	4.13
**-1.82**	97.23	4.24
**-2.29**	98.80	10.71

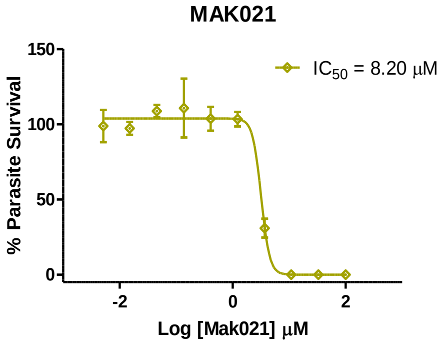

[a]Data are expressed as means of triplicate values. SD = standard deviation; %PS = Percentage Parasite Survival.

Table 15. Log concentration vs. % Parasite survival data used to plot dose-response curve for **4f**.

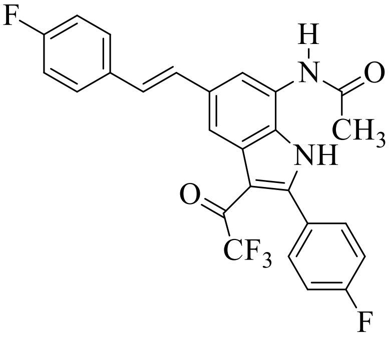

4f

Figure S63. Chemical structure of **4f**

Log Conc (nM)[a]	Mak022	
	%PS	SD
**2.00**	0.00	0.00
**1.52**	0.00	0.00
**1.04**	0.00	0.00
**0.57**	63.56	8.43
**0.09**	97.76	21.21
**-0.39**	83.88	4.74
**-0.86**	87.18	6.48
**-1.34**	98.80	4.11
**-1.82**	94.74	9.07
**-2.29**	94.97	8.18

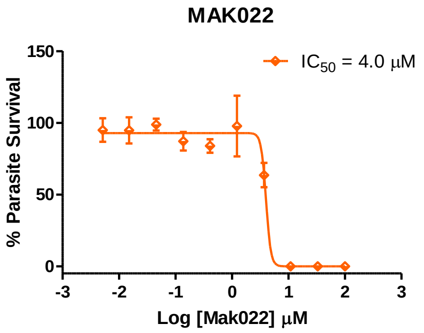

[a]Data are expressed as means of triplicate values. SD = standard deviation; %PS = Percentage Parasite Survival.

Table 16. Log concentration vs. % Parasite survival data used to plot dose-response curve for **4g**.

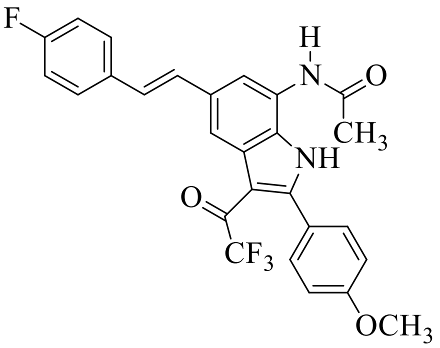

4g

Figure S64. Chemical structure of **4g**

Log Conc (nM)[a]	Mak023	
	%PS	SD
**2.00**	0.00	0.00
**1.52**	0.00	0.00
**1.04**	0.00	0.00
**0.57**	20.17	6.97
**0.09**	97.06	5.47
**-0.39**	98.95	7.36
**-0.86**	102.44	3.56
**-1.34**	93.46	3.39
**-1.82**	101.99	4.62
**-2.29**	98.20	11.43

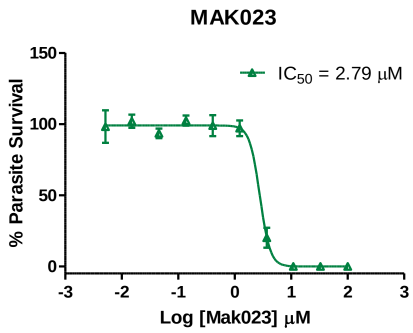

[a]Data are expressed as means of triplicate values. SD = standard deviation; %PS = Percentage Parasite Survival.

Supplementary 3: % cell viability of Vero cells exposed Doxorubicin chloride, 3a, 3f, 4a and 4g

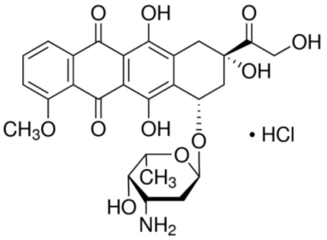

Doxorubicin hydrochloride

Table 17: Percentage cell viability of Vero cells exposed to different concentrations of Doxorubicin hydrochloride

Conc. (µM)	%Viability	SD
100	44.05	3.44
10	63.48	0.93
1	75.68	4.77
0.1	87.74	3.35
0.01	95.00	4.86
0.001	100.00	4.01

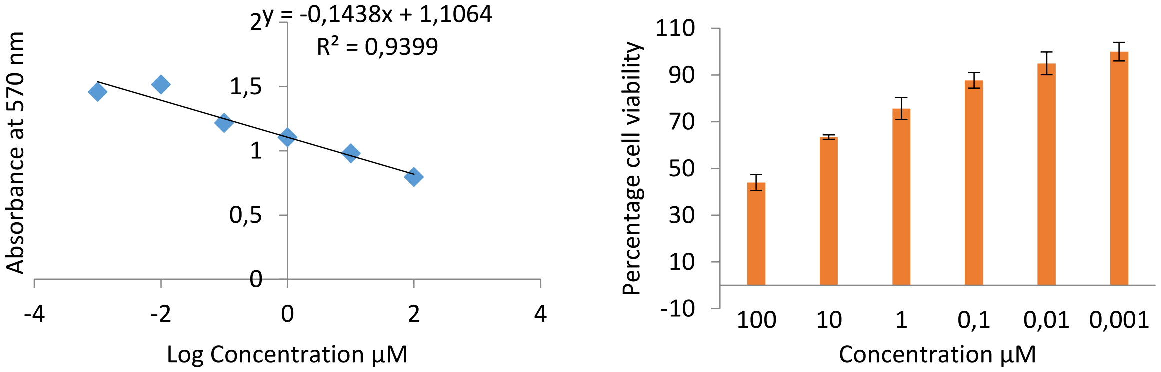

Figure S65. Linear regression plots and percentage cell viability graphs Vero cells exposed to different concentrations of doxorubicin hydrochloride

Table 18: Percentage cell viability of Vero cells exposed to different concentrations of **3a**

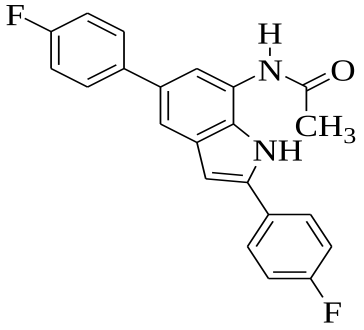

3a

Conc. (µg/ml)	%Viability	SD
100	26.13	0.48
10	51.69	2.71
1	86.54	2.98
0.1	100.00	1.44

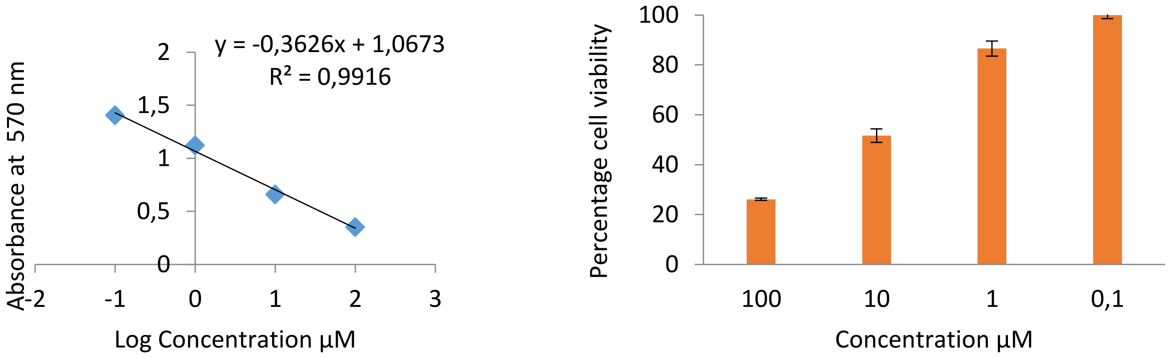

Figure S66. Linear regression plots and percentage cell viability graphs of Vero cells exposed to different concentrations of **3a**

Table 19: Percentage cell viability of Vero cells exposed to different concentrations of **3f**

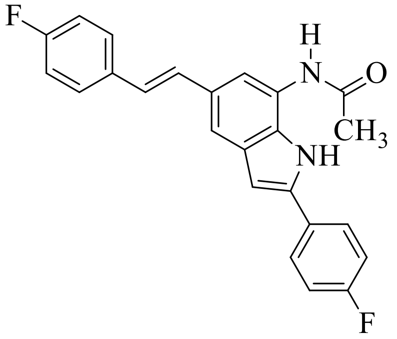

3f

Conc. (µg/ml)	%Viability	SD
100	13.76	7.23
10	94.81	1.81
1	98.5	0.71
0.1	99.00	1.41

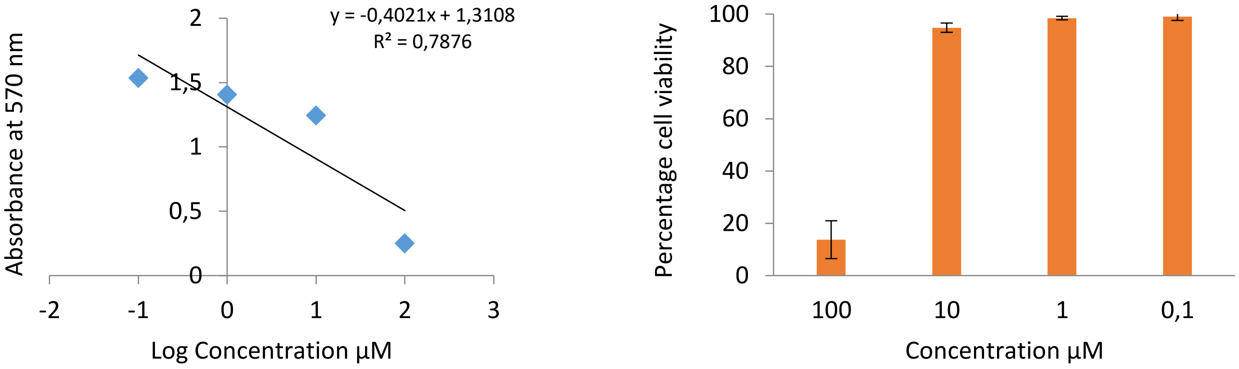

Figure S67. Linear regression plots and percentage cell viability graphs of Vero cells exposed to different concentrations of **3f**

Table 20: Percentage cell viability of Vero cells exposed to different concentrations of **4a**

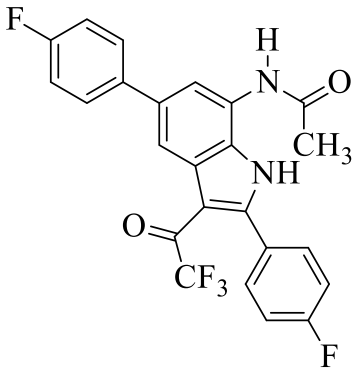

4a

Conc. (µg/ml)	%Viability	SD
100	5.45	0.16
10	96.5	2.12
1	98.50	0.71
0.1	99.5	0.71

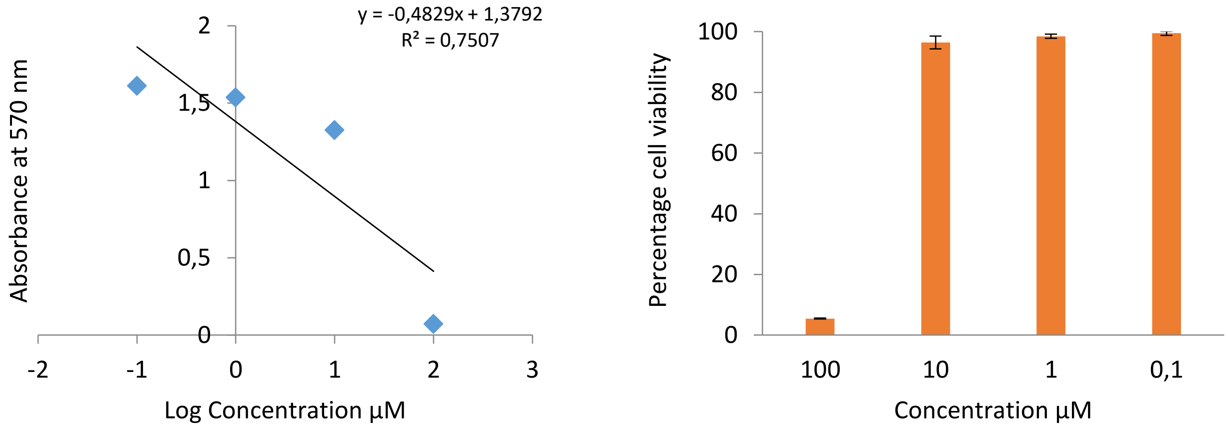

Figure S68. Linear regression plots and percentage cell viability graphs of Vero cells exposed to different concentrations of **4a**

Table 21: Percentage cell viability of Vero cells exposed to different concentrations of **4g**

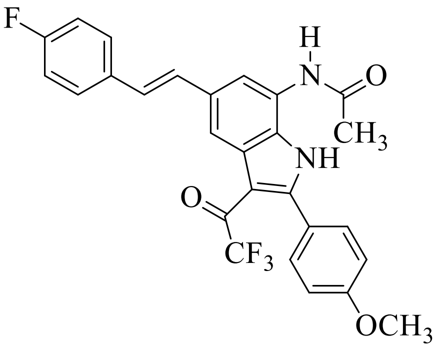

4g

Conc. (µg/ml)	%Viability	SD
100	35.56	0.37
10	98.5	0.71
1	99.00	0.71
0.1	99.50	1.41

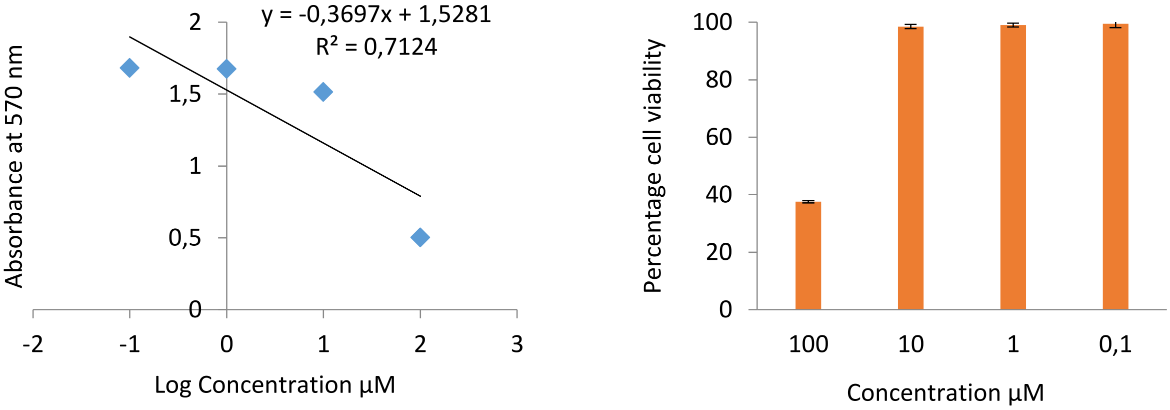

Figure S69. Linear regression plots and percentage cell viability graphs of Vero cells exposed to different concentrations of **4g**

Supplementary 4: Crystal data and structure refinement, bond lengths and torsion angles of 4g

Table 22. Crystal data and structure refinement for 4g

Identification code	16a_mak001_p	
Empirical formula	C27 H20 F4 N2 O3	
Formula weight	496.45	
Temperature	173(2) K	
Wavelength	0.71073 Å	
Crystal system	Triclinic	
Space group	P-1	
Unit cell dimensions	a = 11.1351(10) Å	α= 68.965(3)°.
	b = 14.9334(13) Å	β= 72.856(3)°.
	c = 18.7080(16) Å	γ = 68.716(3)°.
Volume	2656.7(4) $Å^3$	
Z	4	
Density (calculated)	1.241 Mg/m^3	
Absorption coefficient	0.100 mm^{-1}	
F(000)	1024	
Crystal size	0.40 x 0.13 x 0.04 mm^3	
Theta range for data collection	3.06 to 25.50°.	
Index ranges	-11<=h<=13, -17<=k<=18, -22<=l<=22	
Reflections collected	26244	
Independent reflections	9846 [R(int) = 0.0581]	
Completeness to theta = 25.50°	99.5 %	
Absorption correction	Semi-empirical from equivalents	
Max. and min. transmission	0.9960 and 0.9610	
Refinement method	Full-matrix least-squares on F^2	
Data / restraints / parameters	9846 / 0 / 649	
Goodness-of-fit on F^2	1.077	
Final R indices [I>2sigma(I)]	R1 = 0.0788, wR2 = 0.2115	
R indices (all data)	R1 = 0.1203, wR2 = 0.2286	
Largest diff. peak and hole	0.432 and -0.390 e.$Å^{-3}$	

Table 23. Bond lengths [Å] and angles [°] for 4g

C(1)-C(2)	1.355(6)
C(1)-C(6)	1.356(6)
C(1)-F(1)	1.359(4)
C(2)-C(3)	1.406(6)
C(2)-H(2A)	0.9500
C(3)-C(4)	1.359(6)
C(3)-H(3A)	0.9500
C(4)-C(5)	1.404(6)
C(4)-C(7)	1.468(5)
C(5)-C(6)	1.369(5)
C(5)-H(5)	0.9500
C(6)-H(6)	0.9500
C(7)-C(8)	1.286(6)
C(7)-H(7)	0.9500
C(8)-C(9)	1.482(5)
C(8)-H(8)	0.9500
C(9)-C(10)	1.390(5)
C(9)-C(14)	1.411(5)
C(10)-C(11)	1.402(5)
C(10)-H(10)	0.9500
C(11)-C(12)	1.397(5)
C(11)-C(15)	1.445(5)
C(12)-N(1)	1.387(4)
C(12)-C(13)	1.394(5)
C(13)-C(14)	1.367(5)
C(13)-N(2)	1.409(5)
C(14)-H(14)	0.9500
C(15)-C(16)	1.407(5)
C(15)-C(17)	1.457(5)
C(16)-N(1)	1.373(4)
C(16)-C(19)	1.464(5)
C(17)-O(1)	1.208(4)
C(17)-C(18)	1.534(5)
C(18)-F(4)	1.317(4)
C(18)-F(2)	1.336(4)
C(18)-F(3)	1.355(4)

C(19)-C(20)	1.402(5)
C(19)-C(24)	1.403(5)
C(20)-C(21)	1.375(5)
C(20)-H(20)	0.9500
C(21)-C(22)	1.397(5)
C(21)-H(21)	0.9500
C(22)-O(3)	1.358(4)
C(22)-C(23)	1.380(5)
C(23)-C(24)	1.392(5)
C(23)-H(23)	0.9500
C(24)-H(24)	0.9500
C(25)-O(3)	1.431(4)
C(25)-H(25A)	0.9800
C(25)-H(25B)	0.9800
C(25)-H(25C)	0.9800
C(26)-O(2)	1.208(5)
C(26)-N(2)	1.366(5)
C(26)-C(27)	1.531(6)
N(1)-H(1)	0.8800
N(2)-H(2)	0.8800
C(27)-H(27A)	0.9800
C(27)-H(27B)	0.9800
C(27)-H(27C)	0.9800
C(28)-C(33)	1.360(6)
C(28)-C(29)	1.361(5)
C(28)-F(5)	1.365(4)
C(29)-C(30)	1.374(5)
C(29)-H(29)	0.9500
C(30)-C(31)	1.395(5)
C(30)-H(30)	0.9500
C(31)-C(32)	1.403(5)
C(31)-C(34)	1.470(5)
C(32)-C(33)	1.388(5)
C(32)-H(32)	0.9500
C(33)-H(33)	0.9500
C(34)-C(35)	1.338(5)
C(34)-H(34)	0.9500
C(35)-C(36)	1.468(5)
C(35)-H(35)	0.9500

C(36)-C(37)	1.394(5)
C(36)-C(41)	1.419(5)
C(37)-C(38)	1.390(5)
C(37)-H(37)	0.9500
C(38)-C(39)	1.403(5)
C(38)-C(42)	1.455(5)
C(39)-N(3)	1.384(4)
C(39)-C(40)	1.394(5)
C(40)-C(41)	1.381(5)
C(40)-N(4)	1.416(4)
C(41)-H(41)	0.9500
C(42)-C(43)	1.396(5)
C(42)-C(44)	1.445(5)
C(43)-N(3)	1.358(5)
C(43)-C(48)	1.466(5)
C(44)-O(4)	1.230(4)
C(44)-C(45)	1.517(6)
C(45)-F(6)	1.337(5)
C(45)-F(7)	1.345(5)
C(45)-F(8)	1.347(5)
C(48)-C(53)	1.377(5)
C(48)-C(49)	1.402(5)
C(49)-C(50)	1.363(5)
C(49)-H(49)	0.9500
C(50)-C(51)	1.394(5)
C(50)-H(50)	0.9500
C(51)-O(6)	1.364(4)
C(51)-C(52)	1.379(5)
C(52)-C(53)	1.381(5)
C(52)-H(52)	0.9500
C(53)-H(53)	0.9500
C(54)-O(6)	1.410(5)
C(54)-H(54A)	0.9800
C(54)-H(54B)	0.9800
C(54)-H(54C)	0.9800
C(55)-O(5)	1.205(4)
C(55)-N(4)	1.366(5)
C(55)-C(56)	1.509(5)
C(56)-H(56A)	0.9800

C(56)-H(56B)	0.9800
C(56)-H(56C)	0.9800
N(3)-H(3)	0.8800
N(4)-H(4)	0.8800
C(2)-C(1)-C(6)	122.3(4)
C(2)-C(1)-F(1)	117.9(4)
C(6)-C(1)-F(1)	119.8(4)
C(1)-C(2)-C(3)	118.0(4)
C(1)-C(2)-H(2A)	121.0
C(3)-C(2)-H(2A)	121.0
C(4)-C(3)-C(2)	122.2(4)
C(4)-C(3)-H(3A)	118.9
C(2)-C(3)-H(3A)	118.9
C(3)-C(4)-C(5)	116.7(4)
C(3)-C(4)-C(7)	120.7(4)
C(5)-C(4)-C(7)	122.7(4)
C(6)-C(5)-C(4)	122.0(4)
C(6)-C(5)-H(5)	119.0
C(4)-C(5)-H(5)	119.0
C(1)-C(6)-C(5)	118.8(4)
C(1)-C(6)-H(6)	120.6
C(5)-C(6)-H(6)	120.6
C(8)-C(7)-C(4)	128.6(4)
C(8)-C(7)-H(7)	115.7
C(4)-C(7)-H(7)	115.7
C(7)-C(8)-C(9)	127.9(4)
C(7)-C(8)-H(8)	116.0
C(9)-C(8)-H(8)	116.0
C(10)-C(9)-C(14)	120.8(3)
C(10)-C(9)-C(8)	121.3(3)
C(14)-C(9)-C(8)	117.8(3)
C(9)-C(10)-C(11)	117.6(3)
C(9)-C(10)-H(10)	121.2
C(11)-C(10)-H(10)	121.2
C(12)-C(11)-C(10)	120.1(3)
C(12)-C(11)-C(15)	107.0(3)
C(10)-C(11)-C(15)	132.9(3)
N(1)-C(12)-C(13)	128.9(3)
N(1)-C(12)-C(11)	108.4(3)

C(13)-C(12)-C(11)	122.6(3)
C(14)-C(13)-C(12)	116.7(3)
C(14)-C(13)-N(2)	125.0(3)
C(12)-C(13)-N(2)	118.2(3)
C(13)-C(14)-C(9)	122.2(3)
C(13)-C(14)-H(14)	118.9
C(9)-C(14)-H(14)	118.9
C(16)-C(15)-C(11)	106.4(3)
C(16)-C(15)-C(17)	132.8(3)
C(11)-C(15)-C(17)	120.5(3)
N(1)-C(16)-C(15)	108.8(3)
N(1)-C(16)-C(19)	117.4(3)
C(15)-C(16)-C(19)	133.7(3)
O(1)-C(17)-C(15)	122.7(3)
O(1)-C(17)-C(18)	114.1(3)
C(15)-C(17)-C(18)	123.1(3)
F(4)-C(18)-F(2)	108.0(3)
F(4)-C(18)-F(3)	106.3(3)
F(2)-C(18)-F(3)	106.9(3)
F(4)-C(18)-C(17)	110.6(3)
F(2)-C(18)-C(17)	115.0(3)
F(3)-C(18)-C(17)	109.7(3)
C(20)-C(19)-C(24)	117.0(3)
C(20)-C(19)-C(16)	121.4(3)
C(24)-C(19)-C(16)	121.5(3)
C(21)-C(20)-C(19)	121.2(3)
C(21)-C(20)-H(20)	119.4
C(19)-C(20)-H(20)	119.4
C(20)-C(21)-C(22)	120.6(3)
C(20)-C(21)-H(21)	119.7
C(22)-C(21)-H(21)	119.7
O(3)-C(22)-C(23)	125.2(3)
O(3)-C(22)-C(21)	115.1(3)
C(23)-C(22)-C(21)	119.7(3)
C(22)-C(23)-C(24)	119.2(3)
C(22)-C(23)-H(23)	120.4
C(24)-C(23)-H(23)	120.4
C(23)-C(24)-C(19)	122.2(4)
C(23)-C(24)-H(24)	118.9

C(19)-C(24)-H(24)	118.9
O(3)-C(25)-H(25A)	109.5
O(3)-C(25)-H(25B)	109.5
H(25A)-C(25)-H(25B)	109.5
O(3)-C(25)-H(25C)	109.5
H(25A)-C(25)-H(25C)	109.5
H(25B)-C(25)-H(25C)	109.5
O(2)-C(26)-N(2)	124.1(4)
O(2)-C(26)-C(27)	123.2(4)
N(2)-C(26)-C(27)	112.7(4)
C(16)-N(1)-C(12)	109.3(3)
C(16)-N(1)-H(1)	125.3
C(12)-N(1)-H(1)	125.3
C(26)-N(2)-C(13)	127.6(3)
C(26)-N(2)-H(2)	116.2
C(13)-N(2)-H(2)	116.2
C(22)-O(3)-C(25)	117.5(3)
C(26)-C(27)-H(27A)	109.5
C(26)-C(27)-H(27B)	109.5
H(27A)-C(27)-H(27B)	109.5
C(26)-C(27)-H(27C)	109.5
H(27A)-C(27)-H(27C)	109.5
H(27B)-C(27)-H(27C)	109.5
C(33)-C(28)-C(29)	123.0(4)
C(33)-C(28)-F(5)	118.5(4)
C(29)-C(28)-F(5)	118.5(3)
C(28)-C(29)-C(30)	118.6(4)
C(28)-C(29)-H(29)	120.7
C(30)-C(29)-H(29)	120.7
C(29)-C(30)-C(31)	121.6(4)
C(29)-C(30)-H(30)	119.2
C(31)-C(30)-H(30)	119.2
C(30)-C(31)-C(32)	117.4(3)
C(30)-C(31)-C(34)	123.3(3)
C(32)-C(31)-C(34)	119.3(3)
C(33)-C(32)-C(31)	121.0(4)
C(33)-C(32)-H(32)	119.5
C(31)-C(32)-H(32)	119.5
C(28)-C(33)-C(32)	118.4(4)

C(28)-C(33)-H(33)	120.8
C(32)-C(33)-H(33)	120.8
C(35)-C(34)-C(31)	125.7(3)
C(35)-C(34)-H(34)	117.2
C(31)-C(34)-H(34)	117.2
C(34)-C(35)-C(36)	126.7(4)
C(34)-C(35)-H(35)	116.7
C(36)-C(35)-H(35)	116.7
C(37)-C(36)-C(41)	120.2(3)
C(37)-C(36)-C(35)	117.9(3)
C(41)-C(36)-C(35)	121.9(3)
C(38)-C(37)-C(36)	119.5(3)
C(38)-C(37)-H(37)	120.3
C(36)-C(37)-H(37)	120.3
C(37)-C(38)-C(39)	119.1(3)
C(37)-C(38)-C(42)	133.8(3)
C(39)-C(38)-C(42)	106.8(3)
N(3)-C(39)-C(40)	130.0(3)
N(3)-C(39)-C(38)	107.3(3)
C(40)-C(39)-C(38)	122.6(3)
C(41)-C(40)-C(39)	117.7(3)
C(41)-C(40)-N(4)	124.0(3)
C(39)-C(40)-N(4)	118.3(3)
C(40)-C(41)-C(36)	121.0(3)
C(40)-C(41)-H(41)	119.5
C(36)-C(41)-H(41)	119.5
C(43)-C(42)-C(44)	133.3(3)
C(43)-C(42)-C(38)	106.4(3)
C(44)-C(42)-C(38)	120.2(3)
N(3)-C(43)-C(42)	108.6(3)
N(3)-C(43)-C(48)	119.1(3)
C(42)-C(43)-C(48)	132.2(3)
O(4)-C(44)-C(42)	124.1(4)
O(4)-C(44)-C(45)	112.9(3)
C(42)-C(44)-C(45)	123.0(3)
F(6)-C(45)-F(7)	106.4(3)
F(6)-C(45)-F(8)	106.0(4)
F(7)-C(45)-F(8)	107.2(3)
F(6)-C(45)-C(44)	109.8(3)

F(7)-C(45)-C(44)	111.1(4)
F(8)-C(45)-C(44)	115.8(3)
C(53)-C(48)-C(49)	117.6(3)
C(53)-C(48)-C(43)	121.8(3)
C(49)-C(48)-C(43)	120.6(3)
C(50)-C(49)-C(48)	120.7(3)
C(50)-C(49)-H(49)	119.6
C(48)-C(49)-H(49)	119.6
C(49)-C(50)-C(51)	121.0(4)
C(49)-C(50)-H(50)	119.5
C(51)-C(50)-H(50)	119.5
O(6)-C(51)-C(52)	125.6(3)
O(6)-C(51)-C(50)	115.5(3)
C(52)-C(51)-C(50)	118.8(3)
C(51)-C(52)-C(53)	119.7(4)
C(51)-C(52)-H(52)	120.1
C(53)-C(52)-H(52)	120.1
C(48)-C(53)-C(52)	122.1(3)
C(48)-C(53)-H(53)	118.9
C(52)-C(53)-H(53)	118.9
O(6)-C(54)-H(54A)	109.5
O(6)-C(54)-H(54B)	109.5
H(54A)-C(54)-H(54B)	109.5
O(6)-C(54)-H(54C)	109.5
H(54A)-C(54)-H(54C)	109.5
H(54B)-C(54)-H(54C)	109.5
O(5)-C(55)-N(4)	124.2(3)
O(5)-C(55)-C(56)	122.2(3)
N(4)-C(55)-C(56)	113.5(3)
C(55)-C(56)-H(56A)	109.5
C(55)-C(56)-H(56B)	109.5
H(56A)-C(56)-H(56B)	109.5
C(55)-C(56)-H(56C)	109.5
H(56A)-C(56)-H(56C)	109.5
H(56B)-C(56)-H(56C)	109.5
C(43)-N(3)-C(39)	110.7(3)
C(43)-N(3)-H(3)	124.6
C(39)-N(3)-H(3)	124.6
C(55)-N(4)-C(40)	127.8(3)

C(55)-N(4)-H(4)	116.1
C(40)-N(4)-H(4)	116.1
C(51)-O(6)-C(54)	117.9(3)

Symmetry transformations used to generate equivalent atoms:

Table 24. Torsion angles [°] for 4g

C(6)-C(1)-C(2)-C(3)	-2.7(9)
F(1)-C(1)-C(2)-C(3)	-179.9(5)
C(1)-C(2)-C(3)-C(4)	-0.7(10)
C(2)-C(3)-C(4)-C(5)	3.4(9)
C(2)-C(3)-C(4)-C(7)	-176.7(6)
C(3)-C(4)-C(5)-C(6)	-3.0(7)
C(7)-C(4)-C(5)-C(6)	177.1(4)
C(2)-C(1)-C(6)-C(5)	3.1(8)
F(1)-C(1)-C(6)-C(5)	-179.8(4)
C(4)-C(5)-C(6)-C(1)	-0.1(7)
C(3)-C(4)-C(7)-C(8)	171.2(6)
C(5)-C(4)-C(7)-C(8)	-9.0(8)
C(4)-C(7)-C(8)-C(9)	-177.6(4)
C(7)-C(8)-C(9)-C(10)	3.4(7)
C(7)-C(8)-C(9)-C(14)	-177.6(4)
C(14)-C(9)-C(10)-C(11)	0.3(6)
C(8)-C(9)-C(10)-C(11)	179.3(4)
C(9)-C(10)-C(11)-C(12)	0.2(6)
C(9)-C(10)-C(11)-C(15)	-177.6(4)
C(10)-C(11)-C(12)-N(1)	178.6(3)
C(15)-C(11)-C(12)-N(1)	-3.2(4)
C(10)-C(11)-C(12)-C(13)	-0.2(6)
C(15)-C(11)-C(12)-C(13)	178.1(3)
N(1)-C(12)-C(13)-C(14)	-178.7(4)
C(11)-C(12)-C(13)-C(14)	-0.3(6)
N(1)-C(12)-C(13)-N(2)	0.6(6)
C(11)-C(12)-C(13)-N(2)	179.0(3)
C(12)-C(13)-C(14)-C(9)	0.7(6)
N(2)-C(13)-C(14)-C(9)	-178.5(4)
C(10)-C(9)-C(14)-C(13)	-0.7(6)
C(8)-C(9)-C(14)-C(13)	-179.8(4)
C(12)-C(11)-C(15)-C(16)	3.5(4)
C(10)-C(11)-C(15)-C(16)	-178.5(4)
C(12)-C(11)-C(15)-C(17)	-171.6(3)
C(10)-C(11)-C(15)-C(17)	6.3(7)
C(11)-C(15)-C(16)-N(1)	-2.6(4)
C(17)-C(15)-C(16)-N(1)	171.7(4)

C(11)-C(15)-C(16)-C(19)	173.8(4)
C(17)-C(15)-C(16)-C(19)	-11.9(7)
C(16)-C(15)-C(17)-O(1)	170.5(4)
C(11)-C(15)-C(17)-O(1)	-15.9(6)
C(16)-C(15)-C(17)-C(18)	-12.3(7)
C(11)-C(15)-C(17)-C(18)	161.3(3)
O(1)-C(17)-C(18)-F(4)	27.9(5)
C(15)-C(17)-C(18)-F(4)	-149.5(3)
O(1)-C(17)-C(18)-F(2)	150.4(4)
C(15)-C(17)-C(18)-F(2)	-27.0(5)
O(1)-C(17)-C(18)-F(3)	-89.1(4)
C(15)-C(17)-C(18)-F(3)	93.5(4)
N(1)-C(16)-C(19)-C(20)	133.3(4)
C(15)-C(16)-C(19)-C(20)	-42.9(6)
N(1)-C(16)-C(19)-C(24)	-42.5(5)
C(15)-C(16)-C(19)-C(24)	141.4(4)
C(24)-C(19)-C(20)-C(21)	-3.0(5)
C(16)-C(19)-C(20)-C(21)	-178.9(3)
C(19)-C(20)-C(21)-C(22)	1.4(6)
C(20)-C(21)-C(22)-O(3)	179.5(3)
C(20)-C(21)-C(22)-C(23)	0.7(6)
O(3)-C(22)-C(23)-C(24)	-179.7(3)
C(21)-C(22)-C(23)-C(24)	-1.0(6)
C(22)-C(23)-C(24)-C(19)	-0.7(6)
C(20)-C(19)-C(24)-C(23)	2.6(5)
C(16)-C(19)-C(24)-C(23)	178.5(3)
C(15)-C(16)-N(1)-C(12)	0.7(4)
C(19)-C(16)-N(1)-C(12)	-176.4(3)
C(13)-C(12)-N(1)-C(16)	-179.8(4)
C(11)-C(12)-N(1)-C(16)	1.6(4)
O(2)-C(26)-N(2)-C(13)	-1.5(7)
C(27)-C(26)-N(2)-C(13)	179.4(4)
C(14)-C(13)-N(2)-C(26)	2.6(7)
C(12)-C(13)-N(2)-C(26)	-176.6(4)
C(23)-C(22)-O(3)-C(25)	-2.9(5)
C(21)-C(22)-O(3)-C(25)	178.4(3)
C(33)-C(28)-C(29)-C(30)	1.2(6)
F(5)-C(28)-C(29)-C(30)	-179.8(4)
C(28)-C(29)-C(30)-C(31)	0.9(6)

C(29)-C(30)-C(31)-C(32)	-2.2(6)
C(29)-C(30)-C(31)-C(34)	176.0(4)
C(30)-C(31)-C(32)-C(33)	1.5(6)
C(34)-C(31)-C(32)-C(33)	-176.7(4)
C(29)-C(28)-C(33)-C(32)	-1.8(7)
F(5)-C(28)-C(33)-C(32)	179.1(4)
C(31)-C(32)-C(33)-C(28)	0.4(7)
C(30)-C(31)-C(34)-C(35)	-8.0(6)
C(32)-C(31)-C(34)-C(35)	170.2(4)
C(31)-C(34)-C(35)-C(36)	-176.5(4)
C(34)-C(35)-C(36)-C(37)	164.9(4)
C(34)-C(35)-C(36)-C(41)	-14.5(6)
C(41)-C(36)-C(37)-C(38)	0.0(6)
C(35)-C(36)-C(37)-C(38)	-179.4(3)
C(36)-C(37)-C(38)-C(39)	-1.2(6)
C(36)-C(37)-C(38)-C(42)	-174.3(4)
C(37)-C(38)-C(39)-N(3)	-176.5(3)
C(42)-C(38)-C(39)-N(3)	-1.6(4)
C(37)-C(38)-C(39)-C(40)	1.2(6)
C(42)-C(38)-C(39)-C(40)	176.1(3)
N(3)-C(39)-C(40)-C(41)	177.1(4)
C(38)-C(39)-C(40)-C(41)	0.0(6)
N(3)-C(39)-C(40)-N(4)	-3.4(6)
C(38)-C(39)-C(40)-N(4)	179.5(4)
C(39)-C(40)-C(41)-C(36)	-1.2(6)
N(4)-C(40)-C(41)-C(36)	179.3(4)
C(37)-C(36)-C(41)-C(40)	1.3(6)
C(35)-C(36)-C(41)-C(40)	-179.4(4)
C(37)-C(38)-C(42)-C(43)	175.8(4)
C(39)-C(38)-C(42)-C(43)	2.0(4)
C(37)-C(38)-C(42)-C(44)	-1.0(7)
C(39)-C(38)-C(42)-C(44)	-174.7(4)
C(44)-C(42)-C(43)-N(3)	174.4(4)
C(38)-C(42)-C(43)-N(3)	-1.7(4)
C(44)-C(42)-C(43)-C(48)	-7.8(8)
C(38)-C(42)-C(43)-C(48)	176.0(4)
C(43)-C(42)-C(44)-O(4)	161.5(4)
C(38)-C(42)-C(44)-O(4)	-22.8(6)
C(43)-C(42)-C(44)-C(45)	-21.3(7)

C(38)-C(42)-C(44)-C(45)	154.4(4)
O(4)-C(44)-C(45)-F(6)	39.4(5)
C(42)-C(44)-C(45)-F(6)	-138.0(4)
O(4)-C(44)-C(45)-F(7)	-77.9(5)
C(42)-C(44)-C(45)-F(7)	104.6(4)
O(4)-C(44)-C(45)-F(8)	159.4(4)
C(42)-C(44)-C(45)-F(8)	-18.1(6)
N(3)-C(43)-C(48)-C(53)	-45.7(5)
C(42)-C(43)-C(48)-C(53)	136.7(4)
N(3)-C(43)-C(48)-C(49)	134.4(4)
C(42)-C(43)-C(48)-C(49)	-43.1(6)
C(53)-C(48)-C(49)-C(50)	-0.8(5)
C(43)-C(48)-C(49)-C(50)	179.1(4)
C(48)-C(49)-C(50)-C(51)	-0.1(6)
C(49)-C(50)-C(51)-O(6)	-178.1(4)
C(49)-C(50)-C(51)-C(52)	1.4(6)
O(6)-C(51)-C(52)-C(53)	177.8(4)
C(50)-C(51)-C(52)-C(53)	-1.7(6)
C(49)-C(48)-C(53)-C(52)	0.5(6)
C(43)-C(48)-C(53)-C(52)	-179.4(4)
C(51)-C(52)-C(53)-C(48)	0.7(6)
C(42)-C(43)-N(3)-C(39)	0.7(4)
C(48)-C(43)-N(3)-C(39)	-177.3(3)
C(40)-C(39)-N(3)-C(43)	-176.9(4)
C(38)-C(39)-N(3)-C(43)	0.6(4)
O(5)-C(55)-N(4)-C(40)	-2.6(6)
C(56)-C(55)-N(4)-C(40)	178.0(4)
C(41)-C(40)-N(4)-C(55)	-4.7(6)
C(39)-C(40)-N(4)-C(55)	175.8(4)
C(52)-C(51)-O(6)-C(54)	6.4(7)
C(50)-C(51)-O(6)-C(54)	-174.1(4)

Symmetry transformations used to generate equivalent atoms:
